# GSVD Comparison of Patient-Matched Normal and Tumor aCGH Profiles Reveals Global Copy-Number Alterations Predicting Glioblastoma Multiforme Survival

**DOI:** 10.1371/journal.pone.0030098

**Published:** 2012-01-23

**Authors:** Cheng H. Lee, Benjamin O. Alpert, Preethi Sankaranarayanan, Orly Alter

**Affiliations:** 1 Scientific Computing and Imaging (SCI) Institute, University of Utah, Salt Lake City, Utah, United States of America; 2 Department of Bioengineering, University of Utah, Salt Lake City, Utah, United States of America; 3 Department of Human Genetics, University of Utah, Salt Lake City, Utah, United States of America; Johns Hopkins University, United States of America

## Abstract

Despite recent large-scale profiling efforts, the best prognostic predictor of glioblastoma multiforme (GBM) remains the patient's age at diagnosis. We describe a global pattern of tumor-exclusive co-occurring copy-number alterations (CNAs) that is correlated, possibly coordinated with GBM patients' survival and response to chemotherapy. The pattern is revealed by GSVD comparison of patient-matched but probe-independent GBM and normal aCGH datasets from The Cancer Genome Atlas (TCGA). We find that, first, the GSVD, formulated as a framework for comparatively modeling two composite datasets, removes from the pattern copy-number variations (CNVs) that occur in the normal human genome (e.g., female-specific X chromosome amplification) and experimental variations (e.g., in tissue batch, genomic center, hybridization date and scanner), without a-priori knowledge of these variations. Second, the pattern includes most known GBM-associated changes in chromosome numbers and focal CNAs, as well as several previously unreported CNAs in 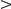
3% of the patients. These include the biochemically putative drug target, cell cycle-regulated serine/threonine kinase-encoding *TLK2*, the cyclin E1-encoding *CCNE1*, and the Rb-binding histone demethylase-encoding *KDM5A*. Third, the pattern provides a better prognostic predictor than the chromosome numbers or any one focal CNA that it identifies, suggesting that the GBM survival phenotype is an outcome of its global genotype. The pattern is independent of age, and combined with age, makes a better predictor than age alone. GSVD comparison of matched profiles of a larger set of TCGA patients, inclusive of the initial set, confirms the global pattern. GSVD classification of the GBM profiles of an independent set of patients validates the prognostic contribution of the pattern.

## Introduction

Glioblastoma multiforme (GBM), the most common brain tumor in adults, is characterized by poor prognosis [1]. GBM tumors exhibit a range of copy-number alterations (CNAs), many of which play roles in the cancer's pathogenesis [2]–[4]. Recent large-scale gene expression [5]–[7] and DNA methylation [8] profiling efforts identified GBM molecular subtypes, distinguished by small numbers of biomarkers. However, despite these efforts, GBM's best prognostic predictor remains the patient's age at diagnosis [9], [10].

To identify CNAs that might predict GBM patients' survival, we comparatively model patient-matched GBM and normal array CGH (aCGH) profiles from The Cancer Genome Atlas (TCGA) by using the generalized singular value decomposition (GSVD) [11]. Previously, we formulated the GSVD as a framework for comparatively modeling two composite datasets [12] (see also [13]), and illustrated its application in sequence-independent comparison of DNA microarray data from two organisms, where, as we showed, the mathematical variables and operations of the GSVD represent experimental or biological reality. The variables, subspaces of significant patterns that are uncovered in the simultaneous decomposition of the two datasets and are mathematically significant in either both (i.e., common to both) datasets or only one (i.e., exclusive to one) of the datasets, correlate with cellular programs that are either conserved in both or unique to only one of the organisms, respectively. The operation of reconstruction in the subspaces that are mathematically common to both datasets outlines the biological similarity in the regulation of the cellular programs that are conserved across the species. Reconstruction in the common and exclusive subspaces of either dataset outlines the differential regulation of the conserved relative to the unique programs in the corresponding organism.

We now find that also in probe-independent comparison of aCGH data from patient-matched tumor and normal samples, the mathematical variables of the GSVD, i.e., shared tumor and normal patterns of copy-number variation across the patients and the corresponding tumor- and normal-specific patterns of copy-number variation across the tumor and normal probes, represent experimental or biological reality. Patterns that are mathematically significant in both datasets represent copy-number variations (CNVs) in the normal human genome that are conserved in the tumor genome (e.g., female-specific X chromosome amplification). Patterns that are mathematically significant in the normal but not the tumor dataset represent experimental variations that exclusively affect the normal dataset. Similarly, some patterns that are mathematically significant in the tumor but not the normal dataset represent experimental variations that exclusively affect the tumor dataset.

One pattern, that is mathematically significant in the tumor but not the normal dataset, represents tumor-exclusive co-occurring CNAs, including most known GBM-associated changes in chromosome numbers and focal CNAs, as well as several previously unreported CNAs in 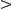
3% of the patients [14]. This pattern is correlated, possibly coordinated with GBM patients' survival and response to therapy. We find that the pattern provides a prognostic predictor that is better than the chromosome numbers or any one focal CNA that it identifies, suggesting that the GBM survival phenotype is an outcome of its global genotype. The pattern is independent of age, and combined with age, makes a better predictor than age alone.

We confirm our results with GSVD comparison of matched profiles of a larger set of TCGA patients, inclusive of the initial set. We validate the prognostic contribution of the pattern with GSVD classification of the GBM profiles of a set of patients that is independent of both the initial set and the inclusive confirmation set [15].

## Methods

To compare TCGA patient-matched GBM and normal (mostly blood) aCGH profiles (Dataset S1 and Mathematica Notebooks S1 and S2), Agilent Human aCGH 244A-measured 365 tumor and 360 normal profiles were selected, corresponding to the same 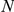 = 251 patients. Each profile lists 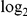 of the TCGA level 1 background-subtracted intensity in the sample relative to the Promega DNA reference, with signal to background 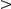
2.5 for both the sample and reference in more than 90% of the 223,603 autosomal probes on the microarray. The profiles are organized in one tumor and one normal dataset, of 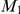 = 212,696 and 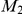 = 211,227 autosomal and X chromosome probes, each probe with valid data in at least 99% of either the tumor or normal arrays, respectively. Each profile is centered at its autosomal median copy number. The 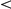
0.2% missing data entries in the tumor and normal datasets are estimated by using singular value decomposition (SVD) as described [12], [16]. Within each set, the medians of profiles of samples from the same patient are taken.

The structure of the patient-matched but probe-independent tumor and normal datasets 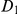 and 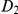, of 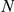 patients, i.e., 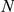-arrays 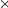 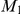-tumor and 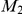-normal probes, is of an order higher than that of a single matrix. The patients, the tumor and normal probes as well as the tissue types, each represent a degree of freedom. Unfolded into a single matrix, some of the degrees of freedom are lost and much of the information in the datasets might also be lost.

**Figure 1:**
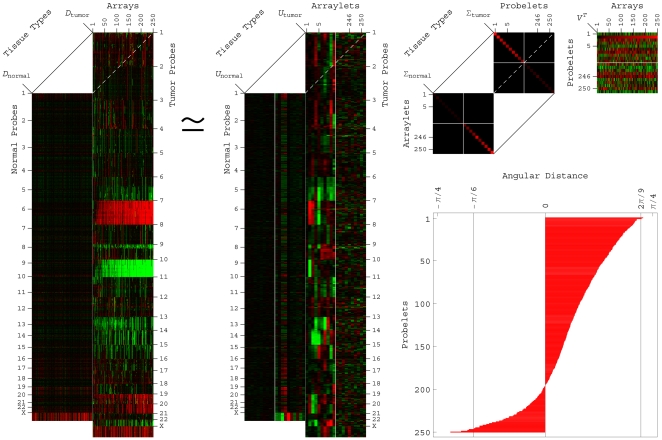
Generalized singular value decomposition (GSVD) of the TCGA patient-matched tumor and normal aCGH profiles. The structure of the patient-matched but probe-independent tumor and normal datasets 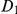 and 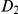, of the initial set of 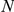 = 251 patients, i.e., 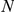-arrays 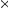 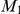 = 212,696-tumor probes and 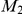 = 211,227-normal probes, is of an order higher than that of a single matrix. The patients, the tumor and normal probes as well as the tissue types, each represent a degree of freedom. Unfolded into a single matrix, some of the degrees of freedom are lost and much of the information in the datasets might also be lost. The GSVD simultaneously separates the paired datasets into paired weighted sums of 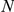 outer products of two patterns each: One pattern of copy-number variation across the patients, i.e., a “probelet” 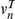, which is identical for both the tumor and normal datasets, combined with either the corresponding tumor-specific pattern of copy-number variation across the tumor probes, i.e., the “tumor arraylet” 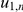, or the corresponding normal-specific pattern across the normal probes, i.e., the “normal arraylet” 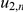 (Equation 1). This is depicted in a raster display, with relative copy-number gain (red), no change (black) and loss (green), explicitly showing only the first though the 10th and the 242nd through the 251st probelets and corresponding tumor and normal arraylets, which capture 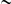
52% and 71% of the information in the tumor and normal dataset, respectively. The significance of the probelet 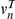 in the tumor dataset relative to its significance in the normal dataset is defined in terms of an “angular distance” that is proportional to the ratio of these weights (Equation 4). This is depicted in a bar chart display, showing that the first and second probelets are almost exclusive to the tumor dataset with angular distances 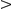
2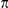/9, the 247th to 251st probelets are approximately exclusive to the normal dataset with angular distances 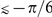, and the 246th probelet is relatively common to the normal and tumor datasets with an angular distance 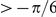. We find and confirm that the second most tumor-exclusive probelet, which is also the most significant probelet in the tumor dataset, significantly correlates with GBM prognosis. The corresponding tumor arraylet describes a global pattern of tumor-exclusive co-occurring CNAs, including most known GBM-associated changes in chromosome numbers and focal CNAs, as well as several previously unreported CNAs, including the biochemically putative drug target-encoding *TLK2* [22]–[25]. We find and validate that a negligible weight of the global pattern in a patient's GBM aCGH profile is indicative of a significantly longer GBM survival time. It was shown that the GSVD provides a mathematical framework for comparative modeling of DNA microarray data from two organisms [12], [39]. Recent experimental results [40] verify a computationally predicted genome-wide mode of regulation [41], [42], and demonstrate that GSVD modeling of DNA microarray data can be used to correctly predict previously unknown cellular mechanisms. This GSVD comparative modeling of aCGH data from patient-matched tumor and normal samples, therefore, draws a mathematical analogy between the prediction of cellular modes of regulation and the prognosis of cancers.

To compare the tumor and normal datasets, therefore, we use the GSVD, formulated to simultaneously separate the paired datasets into paired weighted sums of 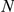 outer products of two patterns each: One pattern of copy-number variation across the patients, i.e., a “probelet” 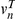, which is identical for both the tumor and normal datasets, combined with either the corresponding tumor-specific pattern of copy-number variation across the tumor probes, i.e., the “tumor arraylet” 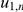, or the corresponding normal-specific pattern across the normal probes, i.e., the “normal arraylet” 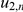 (Figure 1),
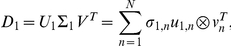


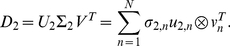
(1)The probelets are, in general, non-orthonormal, but are normalized, such that 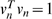. The tumor and normal arraylets are orthonormal, such that 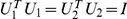.

The significance of the probelet 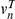 in either the tumor or normal dataset, in terms of the overall information that it captures in this dataset, is proportional to either of the weights 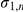 or 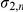, respectively (Figure S1 in Appendix S1),
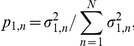


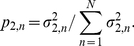
(2)The “generalized normalized Shannon entropy” of each dataset,
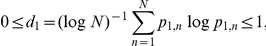


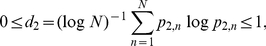
(3)measures the complexity of the data from the distribution of the overall information among the different probelets and corresponding arraylets. An entropy of zero corresponds to an ordered and redundant dataset in which all the information is captured by a single probelet and its corresponding arraylet. An entropy of one corresponds to a disordered and random dataset in which all probelets and arraylets are of equal significance. The significance of the probelet 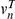 in the tumor dataset relative to its significance in the normal dataset is defined in terms of an “angular distance” 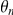 that is proportional to the ratio of these weights,
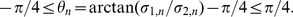
(4)An angular distance of 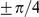 indicates a probelet that is exclusive to either the tumor or normal dataset, respectively, whereas an angular distance of zero indicates a probelet that is common to both the tumor and normal datasets. The probelets are arranged in decreasing order of their angular distances, i.e., their significance in the tumor dataset relative to the normal dataset.

**Figure 2:**
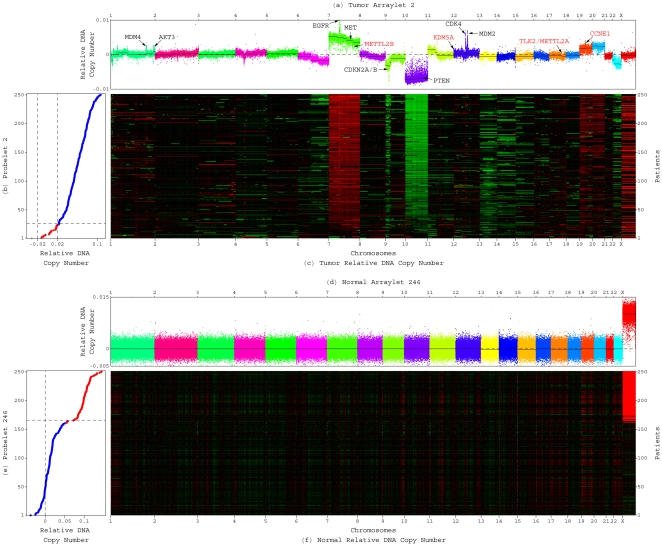
Significant probelets and corresponding tumor and normal arraylets uncovered by GSVD of the patient-matched GBM and normal aCGH profiles. (*a*) Plot of the second tumor arraylet describes a global pattern of tumor-exclusive co-occurring CNAs across the tumor probes. The probes are ordered, and their copy numbers are colored, according to each probe's chromosomal location. Segments (black lines) identified by circular binary segmentation (CBS) [20], [21] include most known GBM-associated focal CNAs (black), e.g., *EGFR* amplification. CNAs previously unrecognized in GBM (red) include an amplification of a segment containing the biochemically putative drug target-encoding *TLK2*. (*b*) Plot of the second most tumor-exclusive probelet, which is also the most significant probelet in the tumor dataset (Figure S1*a* in Appendix S1), describes the corresponding variation across the patients. The patients are ordered and classified according to each patient's relative copy number in this probelet. There are 227 patients (blue) with high (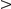
0.02) and 23 patients (red) with low, approximately zero, numbers in the second probelet. One patient (gray) remains unclassified with a large negative (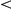
−0.02) number. This classification significantly correlates with GBM survival times (Figure 3*a* and Table S1 in Appendix S1). (*c*) Raster display of the tumor dataset, with relative gain (red), no change (black) and loss (green) of DNA copy numbers, shows the correspondence between the GBM profiles and the second probelet and tumor arraylet. Chromosome 7 gain and losses of chromosomes 9p and 10, which are dominant in the second tumor arraylet (Figure 2*a*), are negligible in the patients with low copy numbers in the second probelet, but distinct in the remaining patients (Figure 2*b*). This illustrates that the copy numbers listed in the second probelet correspond to the weights of the second tumor arraylet in the GBM profiles of the patients. (*d*) Plot of the 246th normal arraylet describes an X chromosome-exclusive amplification across the normal probes. (*e*) Plot of the 246th probelet, which is approximately common to both the normal and tumor datasets, and is the second most significant in the normal dataset (Figure S1*b* in Appendix S1), describes the corresponding copy-number amplification in the female (red) relative to the male (blue) patients. Classification of the patients by the 246th probelet agrees with the copy-number gender assignments (Table 1 and Figure S9 in Appendix S1), also for three patients with missing TCGA gender annotations and three additional patients with conflicting TCGA annotations and copy-number gender assignments. (*f*) Raster display of the normal dataset shows the correspondence between the normal profiles and the 246th probelet and normal arraylet. X chromosome amplification, which is dominant in the 246th normal arraylet (Figure 2*d*), is distinct in the female but nonexisting in the male patients (Figure 2*e*). Note also that although the tumor samples exhibit female-specific X chromosome amplification (Figure 2*c*), the second tumor arraylet (Figure 2*a*) exhibits an unsegmented X chromosome copy-number distribution, that is approximately centered at zero with a relatively small width.

We find that the two most tumor-exclusive mathematical patterns of copy-number variation across the patients, i.e., the first probelet (Figure S2 in Appendix S1) and the second probelet (Figure 2 *a–c*), with angular distances 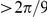, are also the two most significant probelets in the tumor dataset, with 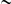
11% and 22% of the information in this dataset, respectively. Similarly, the five most normal-exclusive probelets, the 247th to 251st probelets (Figures S3, S4, S5, S6, S7 in Appendix S1), with angular distances 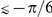, are among the seven most significant probelets in the normal dataset, capturing together 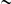
56% of the information in this dataset. The 246th probelet (Figure 2 *d–f*), which is the second most significant probelet in the normal dataset with 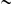
8% of the information, is relatively common to the normal and tumor datasets with an angular distance 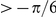.

To biologically or experimentally interpret these significant probelets, we correlate or anticorrelate each probelet with relative copy-number gain or loss across a group of patients according to the TCGA annotations of the group of 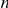 patients with largest or smallest relative copy numbers in this probelet among all 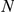 patients, respectively. The *P*-value of a given association is calculated assuming hypergeometric probability distribution of the 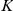 annotations among the 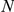 patients, and of the subset of 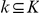 annotations among the subset of 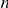 patients, as described [17], 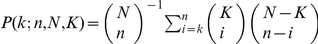 (Table 1). We visualize the copy-number distribution between the annotations that are associated with largest or smallest relative copy numbers in each probelet by using boxplots, and by calculating the corresponding Mann-Whitney-Wilcoxon *P*-value (Figures S8 and S9 in Appendix S1). To interpret the corresponding tumor and normal arraylets, we map the tumor and normal probes onto the National Center for Biotechnology Information (NCBI) human genome sequence build 36, by using the Agilent Technologies probe annotations posted at the University of California at Santa Cruz (UCSC) human genome browser [18], [19]. We segment each arraylet and assign each segment a *P*-value by using the circular binary segmentation (CBS) algorithm as described (Dataset S2) [20], [21]. We find that the significant probelets and corresponding tumor and normal arraylets, as well as their interpretations, are robust to variations in the preprocessing of the data, e.g., in the data selection cutoffs.

**Table 1:** Enrichment of the significant probelets in TCGA annotations.

		Relative DNA Copy Number Gain					Relative DNA Copy Number Loss				
Probelet	Phenotype	Annotation	*n*	*K*	*k*	*P*-value	Annotation	*n*	*K*	*k*	*P*-value
1	Tumor Sample Center	HMS	183	34	34	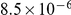	MSKCC	68	103	55	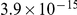
246	Patient Gender	Female	86	86	84	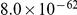	Male	165	165	163	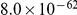
247	Normal Sample Scan Date	10.8.2009	51	6	6	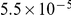	7.22.2009	38	11	10	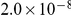
248	Normal Sample Batch/Scanner	HMS 8/2331	19	19	19	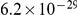	–	–	–	–	–
249	Normal Sample Batch/Scanner	–	–	–	–	–	HMS 8/2331	22	19	19	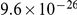
250	Normal Sample Scan Date	4.18.2007	26	9	9	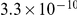	7.22.2009	25	11	9	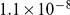
251	Normal Sample Center	HMS	139	46	46	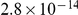	MSKCC	112	101	89	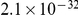

Probabilistic significance of the enrichment of the 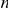 patients, with largest or smallest relative copy numbers in each significant probelet, in the respective TCGA annotations. The *P*-value of each enrichment is calculated assuming hypergeometric probability distribution of the 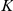 annotations among the 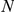 = 251 patients of the initial set, and of the subset of 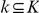 annotations among the subset of 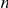 patients, as described [17], 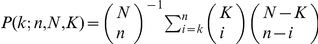.

## Results

We find that, first, the GSVD identifies significant experimental variations that exclusively affect either the tumor or the normal dataset, as well as CNVs that occur in the normal human genome and are common to both datasets, without a-priori knowledge of these variations. The mathematically most tumor-exclusive probelet, i.e., the first probelet (Figure S2 in Appendix S1), correlates with tumor-exclusive experimental variation in the genomic center where the GBM samples were hybridized at, with the *P*-values 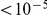 (Table 1 and Figure S8 in Appendix S1). Similarly, the five most normal-exclusive probelets, i.e., the 247th to 251st probelets (Figures S3, S4, S5, S6, S7 in Appendix S1), correlate with experimental variations among the normal samples in genomic center, DNA microarray hybridization or scan date as well as the tissue batch and hybridization scanner, with *P*-values 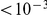. Consistently, the corresponding arraylets, i.e., the first tumor arraylet and the 247th to 251st normal arraylets, describe copy-number distributions which are approximately centered at zero with relatively large, chromosome-invariant widths.

The 246th probelet (Figure 2*e*), which is mathematically approximately common to both the normal and tumor datasets, describes copy-number amplification in the female relative to the male patients that is biologically common to both the normal and tumor datasets. Consistently, both the 246th normal arraylet (Figure 2*d*) and 246th tumor arraylet describe an X chromosome-exclusive amplification. The *P*-values are 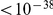 (Table 1 and Figure S9 in Appendix S1). To assign the patients gender, we calculate for each patient the standard deviation of the mean X chromosome number from the autosomal genomic mean in the patient's normal profile (Figure 2*f*). Patients with X chromosome amplification greater than twice the standard deviation are assigned the female gender. For three of the patients, this copy-number gender assignment conflicts with the TCGA gender annotation. For three additional patients, the TCGA gender annotation is missing. In all these cases, the classification of the patients by the 246th probelet agrees with the copy-number assignment.

Second, we find that the GSVD identifies a global pattern of tumor-exclusive co-occurring CNAs that includes most known GBM-associated changes in chromosome numbers and focal CNAs. This global pattern is described by the second tumor arraylet (Figure 2*a* and Dataset S3). The second most tumor-exclusive probelet (Figure 2*b*), which describes the corresponding copy-number variation across the patients, is the most significant probelet in the tumor dataset. Dominant in the global pattern, and frequently observed in GBM samples [2], is a co-occurrence of a gain of chromosome 7 and losses of chromosome 10 and the short arm of chromosome 9 (9p). To assign a chromosome gain or loss, we calculate for each tumor profile the standard deviation of the mean chromosome number from the autosomal genomic mean, excluding the outlying chromosomes 7, 9p and 10. The gain of chromosome 7 and the losses of chromosomes 10 and 9p are greater than twice the standard deviation in the global pattern as well as the tumor profiles of 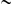
20%, 41% and 12% of the patients, respectively.

Focal CNAs that are known to play roles in the origination and development of GBM and are described by the global pattern include amplifications of segments containing the genes *MDM4* (1q32.1), *AKT3* (1q44), *EGFR* (7p11.2), *MET* (7q31.2), *CDK4* (12q14.1) and *MDM2* (12q15), and deletions of segments containing the genes *CDKN2A*/*B* (9p21.3) and *PTEN* (10q23.31), that occur in 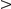
3% of the patients. To assign a CNA in a segment, we calculate for each tumor profile the mean segment copy number. Profiles with segment amplification or deletion greater than twice the standard deviation from the autosomal genomic mean, excluding the outlying chromosomes 7, 9p and 10, or greater than one standard deviation from the chromosomal mean, when this deviation is consistent with the deviation from the genomic mean, are assigned a segment gain or loss, respectively. The frequencies of amplification or deletion we observe for these segments are similar to the reported frequencies of the corresponding focal CNAs [4].

Novel CNAs, previously unrecognized in GBM, are also revealed by the global pattern [14]. These include an amplification of a segment that contains *TLK2* (17q23.2) in 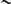
22% of the patients, with the corresponding CBS *P*-value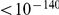. Copy-number amplification of *TLK2* has been correlated with overexpression in several other cancers [22], [23]. The human gene *TLK2*, with homologs in the plant *Arabidopsis thaliana* but not in the yeast *Saccharomyces cerevisiae*, encodes for a multicellular organisms-specific serine/threonine protein kinase, a biochemically putative drug target [24], which activity is directly dependent on ongoing DNA replication [25]. On the same segment with *TLK2*, we also find the gene *METTL2A*. Another amplified segment (CBS *P*-value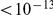) contains the homologous gene *METTL2B* (7q32.1). Overexpression of *METTL2A/B* was linked with prostate cancer metastasis [26], cAMP response element-binding (CREB) regulation in myeloid leukemia [27], and breast cancer patients' response to chemotherapy [28].

An amplification of a segment (CBS *P*-value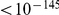) encompassing the cyclin E1-encoding *CCNE1* (19q12) is revealed in 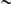
4% of the patients. Cyclin E1 regulates entry into the DNA synthesis phase of the cell division cycle. Copy number increases of *CCNE1* have been linked with multiple cancers [29], [30], but not GBM. Amplicon-dependent expression of *CCNE1*, together with the genes *POP4*, *PLEKHF1*, *C19orf12* and *C19orf2* that flank *CCNE1* on this segment, was linked with primary treatment failure in ovarian cancer, possibly due to rapid repopulation of the tumor after chemotherapy [31].

Another rare amplification in 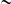
4% of the patients, of a segment (CBS *P*-value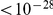) that overlaps with the 5′ end of *KDM5A* (12p13.33), is also revealed. The protein encoded by *KDM5A*, a retinoblastoma tumor suppressor (Rb)-binding lysine-specific histone demethylase [32], has been recently implicated in cancer drug tolerance [33]. The same amplified segment includes the solute carrier (SLC) sodium-neurotransmitter symporters *SLC6A12/13*, biochemically putative carriers of drugs that might overcome the blood-brain barrier [34]. On the same segment we also find *IQSEC3*, a mature neuron-specific guanine nucleotide exchange factor (GEF) for the ADP-ribosylation factor (ARF) *ARF1*, a key regulator of intracellular membrane traffic [35].

Note that although the tumor samples exhibit female-specific X chromosome amplification (Figure 2*c*), the second tumor arraylet exhibits an unsegmented X chromosome copy-number distribution, that is approximately centered at zero with a relatively small width. This illustrates the mathematical separation of the global pattern of tumor-exclusive co-occurring CNAs, that is described by the second tumor arraylet, from all other biological and experimental variations that compose either the tumor or the normal dataset, such as the gender variation that is common to both datasets, and is described by the 246th probelet and the corresponding 246th tumor and 246th normal arraylets.

**Figure 3:**
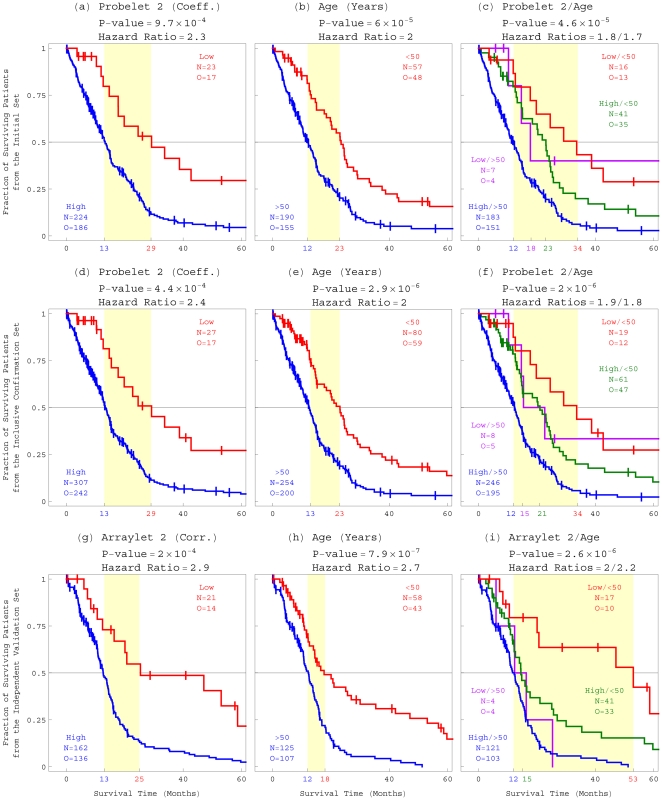
Survival analyses of the three sets of patients classified by GSVD, age at diagnosis or both. (*a*) Kaplan-Meier (KM) [36] curves for the 247 patients with TCGA annotations in the initial set of 251 patients, classified by copy numbers in the second probelet, which is computed by GSVD for the 251 patients, show a median survival time difference of 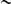
16 months, with the corresponding log-rank test *P*-value 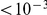. The univariate Cox [37] proportional hazard ratio is 2.3, with a *P*-value 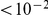 (Table S1), meaning that high relative copy numbers in the second probelet confer more than twice the hazard of low numbers. The *P*-values are calculated without adjusting for multiple comparisons [38]. (*b*) Survival analyses of the 247 patients classified by age, i.e., 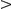
50 or 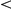
50 years old at diagnosis, show that the prognostic contribution of age, with a KM median survival time difference of 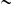
11 months and a univariate Cox hazard ratio of 2, is comparable to that of GSVD. (*c*) Survival analyses of the 247 patients classified by both GSVD and age, show similar multivariate Cox hazard ratios, of 1.8 and 1.7, that do not differ significantly from the corresponding univariate hazard ratios, of 2.3 and 2, respectively. This means that GSVD and age are independent prognostic predictors. With a KM median survival time difference of 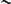
22 months, GSVD and age combined make a better predictor than age alone. (*d*) Survival analyses of the 334 patients with TCGA annotations and a GSVD classification in the inclusive confirmation set of 344 patients, classified by copy numbers in the second probelet, which is computed by GSVD for the 344 patients, show a KM median survival time difference of 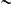
16 months and a univariate hazard ratio of 2.4, and confirm the survival analyses of the initial set of 251 patients. (*e*) Survival analyses of the 334 patients classified by age confirm that the prognostic contribution of age, with a KM median survival time difference of 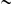
10 months and a univariate hazard ratio of 2, is comparable to that of GSVD. (*f*) Survival analyses of the 334 patients classified by both GSVD and age, show similar multivariate Cox hazard ratios, of 1.9 and 1.8, that do not differ significantly from the corresponding univariate hazard ratios, and a KM median survival time difference of 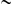
22 months, with the corresponding log-rank test *P*-value 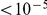. This confirms that the prognostic contribution of GSVD is independent of age, and that combined with age, GSVD makes a better predictor than age alone. (*g*) Survival analyses of the 183 patients with a GSVD classification in the independent validation set of 184 patients, classified by correlations of each patient's GBM profile with the second tumor arraylet, which is computed by GSVD for the 251 patients, show a KM median survival time difference of 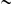
12 months and a univariate hazard ratio of 2.9, and validate the survival analyses of the initial set of 251 patients. (*h*) Survival analyses of the 183 patients classified by age validate that the prognostic contribution of age is comparable to that of GSVD. (*i*) Survival analyses of the 183 patients classified by both GSVD and age, show similar multivariate Cox hazard ratios, of 2 and 2.2, and a KM median survival time difference of 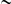
41 months, with the corresponding log-rank test *P*-value 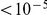. This validates that the prognostic contribution of GSVD is independent of age, and that combined with age, GSVD makes a better predictor than age alone, also for patients with measured GBM aCGH profiles in the absence of matched normal profiles.

Third, we find that the GSVD classifies the patients into two groups of significantly different prognoses. The classification is according to the copy numbers listed in the second probelet, which correspond to the weights of the second tumor arraylet in the GBM aCGH profiles of the patients. A group of 227 patients, 224 of which with TCGA annotations, displays high (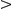
0.02) relative copy numbers in the second probelet, and a Kaplan-Meier (KM) [36] median survival time of 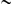
13 months (Figure 3*a*). A group of 23 patients, i.e., 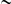
10% of the patients, displays low, approximately zero, relative copy numbers in the second probelet, and a KM median survival time of 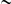
29 months, which is more than twice longer than that of the previous group. The corresponding log-rank test *P*-value is 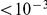. The univariate Cox [37] proportional hazard ratio is 2.3, with a *P*-value 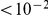 (Table S1 in Appendix S1), meaning that high relative copy numbers in the second probelet confer more than twice the hazard of low numbers. Note that the cutoff of 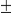
0.02 was selected to enable classification of as many of the patients as possible. Only one of the 251 patients has a negative copy number in the second probelet 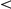
−0.02, and remains unclassified. This patient is also missing the TCGA annotations. Survival analysis of only the chemotherapy patients classified by GSVD gives similar results (Table S2 and Figure S10*a* in Appendix S1). The *P*-values are calculated without adjusting for multiple comparisons [38]. We observe, therefore, that a negligible weight of the global pattern in a patient's GBM aCGH profile is indicative of a significantly longer survival time, as well as an improved response to treatment among chemotherapy patients.

A mutation in the gene *IDH1* was recently linked with improved GBM prognosis [1], [6] and associated with a CpG island methylator phenotype [8]. We find, however, only seven patients (six chmeotherapy patients), i.e., 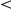
3%, with *IDH1* mutation. This is less than a third of the 23 patients in the long-term survival group defined by the global pattern. The corresponding survival analyses are, therefore, statistically insignificant (Figures S11 and S12 in Appendix S1).

Chromosome 10 loss, chromosome 7 gain and even loss of 9p, which are dominant in the global pattern, have been suggested as indicators of poorer GBM prognoses for over two decades [2], [3]. However, the KM survival curves for the groups of patients with either one of these chromosome number changes almost overlap the curves for the patients with no changes (Figure S13 in Appendix S1). The log-rank test *P*-values for all three classifications are 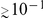, with the median survival time differences 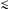
3 months. Similarly, in the KM survival analyses of the groups of patients with either a CNA or no CNA in either one of the 130 segments identified by the global pattern (Figure S14 in Appendix S1), log-rank test *P*-values 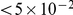 are calculated for only 12 of the classifications. Of these, only six correspond to a KM median survival time difference that is 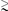
5 months, approximately a third of the 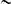
16 months difference observed for the GSVD classification.

One of these segments contains the genes *TLK2* and *METTL2A* and another segment contains the homologous gene *METTL2B*, previously unrecognized in GBM. The KM median survival times we calculate for the 56 patients with *TLK2/METTL2A* amplification and, separately, for the 19 patients with *METTL2B* amplifications are 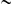
5 and 8 months longer than that for the remaining patients in each case. Similarly, the KM median survival times we calculate for the 43 chemotherapy patients with *TLK2/METTL2A* amplification and, separately, for the 15 chemotherapy patients with *METTL2B* amplification, are both 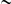
7 months longer than that for the remaining chemotherapy patients in each case (Figure S15 in Appendix S1). This suggests that drug-targeting the kinase that *TLK2* encodes and/or the methyltransferase-like proteins that *METTL2A/B* encode may affect not only the pathogenesis but also the prognosis of GBM as well as the patient's response to chemotherapy.

Taken together, we find that the global pattern provides a better prognostic predictor than the chromosome numbers or any one focal CNA that it identifies. This suggests that the GBM survival phenotype is an outcome of its global genotype.

Despite the recent genome-scale molecular profiling efforts, age at diagnosis remains the best prognostic predictor for GBM in clinical use. The KM median survival time difference between the patients 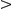
50 or 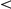
50 years old at diagnosis is 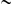
11 months, approximately two thirds of the 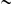
16 months difference observed for the global pattern, with the log-rank test *P*-value 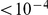 (Figure 3*b*). The univariate Cox proportional hazard ratio we calculate for age is 2, i.e., similar to that for the global pattern. Taken together, the prognostic contribution of the global pattern is comparable to that of age. Similarly we find that the prognostic contribution of the global pattern is comparable to that of chemotherapy (Figure S16*a* in Appendix S1).

To examine whether the weight of the global pattern in a patient's GBM aCGH profile is correlated with the patient's age at diagnosis, we classify the patients into four groups, with prognosis of longer-term survival according to both, only one or neither of the classifications (Figure 3*c*). The KM curves for these four groups are significantly different, with the log-rank test *P*-value 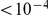. Within each age group, the subgroup of patients with low relative copy numbers in the second probelet consistently exhibits longer survival than the remaining patients. The median survival time of the 16 patients 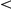
50 years old at diagnosis with low copy numbers in the second probelet is 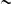
34 months, almost three times longer than the 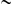
12 months median survival time of the patients 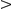
50 years old at diagnosis with high numbers in the second probelet. The multivariate Cox proportional hazard ratios for the global pattern and age are 1.8 and 1.7, respectively, with both corresponding *P*-values 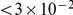. These ratios are similar, meaning that both a high weight of the global pattern in a patient's GBM aCGH profile and an age 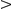
50 years old at diagnosis confer similar relative hazard. These ratios also do not differ significantly from the univariate ratios of 2.3 and 2 for the global pattern and age, respectively. Taken together, the prognostic contribution of the global pattern is not only comparable to that of age, but is also independent of age. Combined with age, the global pattern makes a better predictor than age alone. Similarly, we find that the global pattern is independent of chemotherapy (Figure S16*b* in Appendix S1).

To confirm the global pattern, we use GSVD to compare matched profiles of a larger, more recent, set of 344 TCGA patients, that is inclusive of the initial set of 251 patients [15]. Agilent Human aCGH 244A-measured 458 tumor and 459 normal profiles were selected, corresponding to the inclusive confirmation set of 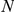 = 344 patients (Dataset S4). The profiles, centered at their autosomal median copy numbers, are organized in one tumor and one normal dataset, of 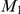 = 200,139 and 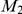 = 198,342 probes, respectively. Within each set, the medians of profiles of samples from the same patient are taken after estimating missing data by using SVD. We find that the significant probelets and corresponding tumor and normal arraylets, as well as their interpretations, are robust to the increase from 251 patients in the initial set to 344 patients in the inclusive confirmation set, and the accompanying decreases in tumor and normal probes, respectively.

The second tumor arraylet computed by GSVD for the 344 patients of the inclusive confirmation set correlates with that of the initial set, with the correlation 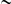
0.99. To classify the patients according to the copy numbers listed in the corresponding second probelet of the inclusive confirmation set, the classification cutoff 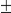
0.02 of the initial set of 251 patients is scaled by the norm of the copy numbers listed for these patients, resulting in the cutoff 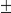
0.017. Only four of the 251 patients in the initial set, i.e., 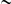
1.5%, with copy numbers that are near the classification cutoffs of both sets, change classification. Of the 344 patients, we find that 315 patients, 309 with TCGA annotations, display high (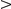
0.017) and 27, i.e., 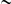
8%, display low, approximately zero, relative copy numbers in the second probelet. Only two patients, one missing TCGA annotations, remain unclassified with large negative (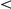
−0.017) copy numbers in the second probelet. Survival analyses of the inclusive confirmation set of 344 patients give qualitatively the same results as these of the initial set of 251 patients. These analyses confirm that a negligible weight of the global pattern, which is described by the second tumor arraylet, i.e., a low copy number in the second probelet, is indicative of a significantly longer survival time (Figure 3*d*). Survival analysis of only the chemotherapy patients in the inclusive confirmation set classified by GSVD gives similar results (Figure S10*b* in Appendix S1). These analyses confirm that the prognostic contribution of the global pattern is comparable to that of age (Figure 3*e*) and is independent of age (Figure 3*f*). Similarly, we confirm that the global pattern is independent of chemotherapy (Figures S16 *c* and *d* in Appendix S1).

To validate the prognostic contribution of the global pattern, we classify GBM profiles of an independent set of 184 TCGA patients, that is mutually exclusive of the initial set of 251 patients. Agilent Human aCGH 244A-measured 280 tumor profiles were selected, corresponding to the independent validation set of 184 patients with available TCGA status annotations (Dataset S5). Each profile lists relative copy numbers in more than 97.5% of the 206,820 autosomal probes among the 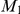 = 212,696 probes that define the second tumor arraylet computed by GSVD for the 251 patients of the initial set. Medians of profiles of samples from the same patient are taken. To classify the 184 patients according to the correlations of their GBM profiles with the second tumor arraylet of the initial set, the classification cutoff of the initial set of 251 patients is scaled by the norm of the correlations calculated for these patients, resulting in the cutoff 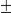
0.15. For the profiles of 162 patients we calculate high (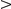
0.15) and for 21, i.e., 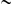
11%, low, approximately zero, correlation with the second tumor arraylet. One patient remains unclassified with a large negative (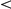
−0.15) correlation.

We find that survival analyses of the independent validation set of 184 patients give qualitatively the same results as these of the initial set of 251 patients and the inclusive confirmation set of 344 patients (Figures 2 *g*–*i* and Figures S10*c*, and S16 *e* and *f* in Appendix S1). These analyses validate the prognostic contribution of the global pattern, which is computed by GSVD of patient-matched tumor and normal aCGH profiles, also for patients with measured GBM aCGH profiles in the absence of matched normal profiles.

## Discussion

Previously, we showed that the GSVD provides a mathematical framework for sequence-independent comparative modeling of DNA microarray data from two organisms, where the mathematical variables and operations represent experimental or biological reality [12], [29]. The variables, subspaces of significant patterns that are common to both or exclusive to either one of the datasets, correlate with cellular programs that are conserved in both or unique to either one of the organisms, respectively. The operation of reconstruction in the subspaces common to both datasets outlines the biological similarity in the regulation of the cellular programs that are conserved across the species. Reconstruction in the common and exclusive subspaces of either dataset outlines the differential regulation of the conserved relative to the unique programs in the corresponding organism. Recent experimental results [40] verify a computationally predicted genome-wide mode of regulation [41], [42], and demonstrate that GSVD modeling of DNA microarray data can be used to correctly predict previously unknown cellular mechanisms.

Recently, we mathematically defined a higher-order GSVD (HO GSVD) for more than two large-scale matrices with different row dimensions and the same column dimension [13]. We proved that this novel HO GSVD extends to higher orders almost all of the mathematical properties of the GSVD. We showed, comparing global mRNA expression from the three disparate organisms *S. pombe*, *S. cerevisiae* and human, that the HO GSVD provides a sequence-independent comparative framework for more than two genomic datasets, where the variables and operations represent experimental or biological reality. The approximately common HO GSVD subspace represents biological similarity among the organisms. Simultaneous reconstruction in the common subspace removes the experimental artifacts, which are dissimilar, from the datasets.

We now show that also in probe-independent comparison of aCGH data from patient-matched tumor and normal samples, the mathematical variables of the GSVD, i.e., shared probelets and the corresponding tumor- and normal-specific arraylets, represent experimental or biological reality. Probelets that are mathematically significant in both datasets, correspond to normal arraylets representing copy-number variations (CNVs) in the normal human genome that are conserved in the tumor genome (e.g., female-specific X chromosome amplification) and are represented by the corresponding tumor arraylets. Probelets that are mathematically significant in the normal but not the tumor dataset represent experimental variations that exclusively affect the normal dataset. Similarly, some probelets that are mathematically significant in the tumor but not the normal dataset represent experimental variations that exclusively affect the tumor dataset.

We find that the mathematically second most tumor-exclusive probelet, which is also the mathematically most significant probelet in the tumor dataset, is statistically correlated, possibly biologically coordinated with GBM patients' survival and response to chemotherapy. The corresponding tumor arraylet describes a global pattern of tumor-exclusive co-occurring CNAs, including most known GBM-associated changes in chromosome numbers and focal CNAs, as well as several previously unreported CNAs, including the biochemically putative drug target-encoding *TLK2* [14]. We find that a negligible weight of the second tumor arraylet in a patient's GBM aCGH profile, mathematically defined by either the corresponding copy number in the second probelet, or by the correlation of the GBM profile with the second arraylet, is indicative of a significantly longer GBM survival time. This GSVD comparative modeling of aCGH data from patient-matched tumor and normal samples, therefore, draws a mathematical analogy between the prediction of cellular modes of regulation and the prognosis of cancers.

We confirm our results with GSVD comparison of matched profiles of a larger set of TCGA patients, inclusive of the initial set. We validate the prognostic contribution of the pattern with GSVD classification of the GBM profiles of a set of patients that is independent of both the initial set and the inclusive confirmation set [15].

Additional possible applications of the GSVD (and also the HO GSVD) in personalized medicine include comparisons of multiple patient-matched datasets, each corresponding to either (*i*) a set of large-scale molecular biological profiles (such as DNA copy numbers) acquired by a high-throughput technology (such as DNA microarrays) from the same tissue type (such as tumor or normal); or (*ii*) a set of biomedical images or signals; or (*iii*) a set of anatomical or clinical pathology test results or phenotypical observations (such as age). GSVD comparisons can uncover the relations and possibly even causal coordinations between these different recorded aspects of the same medical phenomenon. GSVD comparisons can be used to determine a single patient's medical status in relation to all the other patients in the set, and inform the patient's diagnosis, prognosis and treatment.

## Supporting Information

Appendix S1 **Figures S1, S2, S3, S4, S5, S6, S7, S8, S9, S10, S11, S12, S13, S14, S15, S16 and Tables S1 and S2.** A PDF format file, readable by Adobe Acrobat Reader.
(PDF)

Mathematica Notebook S1 **Generalized singular value decomposition (GSVD) of the TCGA patient-matched tumor and normal aCGH profiles.** A Mathematica 8.0.1 code file, executable by Mathematica 8.0.1 and readable by Mathematica Player, freely available at http://www.wolfram.com/products/player/.
(NB) Click here for additional data file.

Mathematica Notebook S2 **Generalized singular value decomposition (GSVD) of the TCGA patient-matched tumor and normal aCGH profiles.** A PDF format file, readable by Adobe Acrobat Reader.
(PDF)

Dataset S1
**Initial set of 251 patients.** A tab-delimited text format file, readable by both Mathematica and Microsoft Excel, reproducing The Cancer Genome Atlas (TCGA) [4] annotations of the initial set of 251 patients and the corresponding normal and tumor samples. The tumor and normal profiles of the initial set of 251 patients, in tab-delimited text format files, tabulating 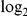 relative copy number variation across 212,696 and 211,227 tumor and normal probes, respectively, are available at http://www.alterlab.org/GBM_prognosis/.(TXT)

Dataset S2 **Segments of the significant tumor and normal arraylets, computed by GSVD for the initial set of 251 patients.** A tab-delimited text format file, readable by both Mathematica and Microsoft Excel, tabulating segments identified by circular binary segmentation (CBS) [20], [21].
(TXT)

Dataset S3 **Segments of the second tumor arraylet, computed by GSVD for the initial set of 251 patients.** A tab-delimited text format file, readable by both Mathematica and Microsoft Excel, tabulating, for each of the 130 CBS segments of the second tumor arraylet, the segment's coordinates, the CBS *P*-value, and the log-rank test *P*-value corresponding to the Kaplan-Meier (KM) survival analysis of the initial set of 251 patients classified by either a gain or a loss of this segment.
(TXT)

Dataset S4
**Inclusive confirmation set of 344 patients.** A tab-delimited text format file, readable by both Mathematica and Microsoft Excel, reproducing the TCGA annotations of the inclusive confirmation set of 344 patients. The tumor and normal profiles of the inclusive confirmation set of 344 patients, in tab-delimited text format files, tabulating 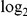 relative copy number variation across 200,139 and 198,342 tumor and normal probes, respectively, are available at http://www.alterlab.org/GBM_prognosis/.(TXT)

Dataset S5
**Independent validation set of 184 patients.** A tab-delimited text format file, readable by both Mathematica and Microsoft Excel, reproducing the TCGA annotations of the independent validation set of 184 patients. The tumor profiles of the independent validation set of 184 patients, in a tab-delimited text format file, tabulating 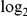 relative copy number variation across 212,696 autosomal and X chromosome probes, are available at http://www.alterlab.org/GBM_prognosis/.(TXT)
