# Supplementary material for: GSVD Comparison of Patient-Matched Normal and Tumor aCGH Profiles Reveals Global Copy-Number Alterations Predicting Glioblastoma Multiforme Survival
Source: PLoS One. 2012 Jan 23;7(1):e30098. doi: 10.1371/journal.pone.0030098 (PMC3264559; doi:10.1371/journal.pone.0030098)
Supplement: Appendix S1 — Figures S1, S2, S3, S4, S5, S6, S7, S8, S9, S10, S11, S12, S13, S14, S15, S16 and Tables S1 and S2. A PDF format file, readable by Adobe Acrobat Reader. (PDF) [file pone.0030098.s001.pdf]

Supplementary Tables and Figures

Cox Proportional Hazard Model	Predictor	Initial Set		Inclusive Confirmation Set		Independent Validation Set	
		Hazard Ratio	P-value	Hazard Ratio	P-value	Hazard Ratio	P-value
Univariate	GSVD	2.3	1.3×10^{-3}	2.4	6.5×10^{-4}	2.9	3.6×10^{-4}
	Age	2.0	7.9×10^{-5}	2.0	4.3×10^{-6}	2.7	1.7×10^{-6}
Multivariate	GSVD	1.8	2.2×10^{-2}	1.9	1.2×10^{-2}	2.0	2.2×10^{-2}
	Age	1.7	2.0×10^{-3}	1.8	1.0×10^{-4}	2.2	2.0×10^{-4}

Table S1. Cox proportional hazard models of the three sets of patients classified by GSVD, age at diagnosis or both. In each set of patients, the multivariate Cox proportional hazard ratios [37] for GSVD and age are similar and do not differ significantly from the corresponding univariate hazard ratios. This means that GSVD and age are independent prognostic predictors.

Cox Proportional Hazard Model	Predictor	Initial Set		Inclusive Confirmation Set		Independent Validation Set	
		Hazard Ratio	P-value	Hazard Ratio	P-value	Hazard Ratio	P-value
Univariate	GSVD	2.4	1.2×10^{-3}	2.4	6.4×10^{-4}	2.8	1.3×10^{-3}
	Chemotherapy	2.6	1.5×10^{-8}	2.7	6.3×10^{-11}	2.2	7.3×10^{-4}
Multivariate	GSVD	3.0	5.2×10^{-5}	3.1	2.5×10^{-5}	3.3	2.3×10^{-4}
	Chemotherapy	3.1	7.9×10^{-11}	3.2	1.9×10^{-13}	2.7	3.0×10^{-5}

Table S2. Cox proportional hazard models of the three sets of patients classified by GSVD, chemotherapy or both. In each set of patients, the multivariate Cox proportional hazard ratios for GSVD and chemotherapy are similar and do not differ significantly from the corresponding univariate hazard ratios. This means that GSVD and chemotherapy are independent prognostic predictors. The P-values are calculated without adjusting for multiple comparisons [38].

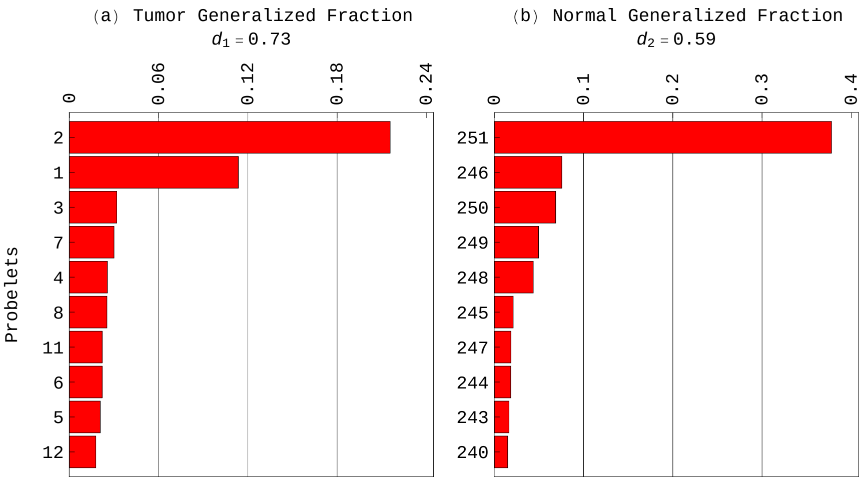

Figure S1. Most significant probelets in the tumor and normal datasets. (a) Bar chart of the ten most significant probelets in the tumor dataset in terms of the generalized fraction that each probelet captures in this dataset (Equation 2), showing that the two most tumor-exclusive probelets, i.e., the first probelet (Figure S2) and the second probelet (Figure 2 a–c), with angular distances $>2\pi/9$, are also the two most significant probelets in the tumor dataset, with ~11% and 22% of the information in this dataset, respectively. The "generalized normalized Shannon entropy" (Equation 3) of the tumor dataset is d_1=0.73. (b) Bar chart of the generalized fractions of the ten most significant probelets in the normal dataset, showing that the five most normal-exclusive probelets, the 247th to 251st probelets (Figures S3–S7), with angular distances $\lesssim -\pi/6$, are among the seven most significant probelets in the normal dataset, capturing together ~56% of the information in this dataset. The 246th probelet (Figure 1 d–f), which is relatively common to the normal and tumor datasets with an angular distance $>-\pi/6$, is the second most significant probelet in the normal dataset with ~8% of the information. The generalized entropy of the normal dataset, d_2=0.59, is smaller than that of the tumor dataset. This means that the normal dataset is more redundant and less complex than the tumor dataset.

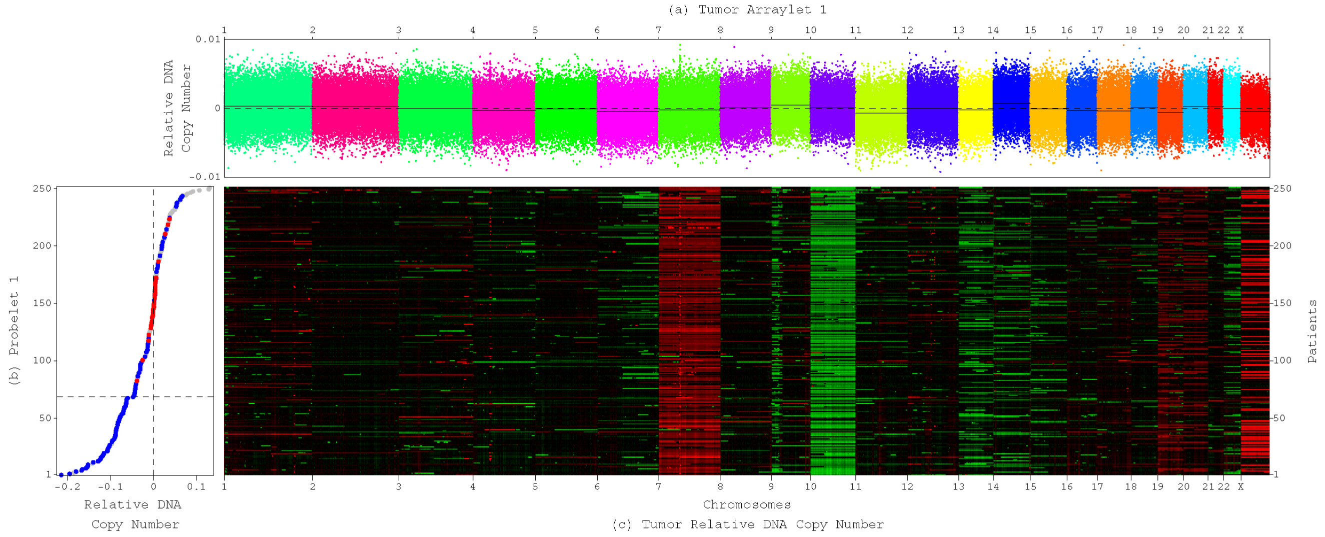

Figure S2. The first most tumor-exclusive probelet and corresponding tumor arraylet uncovered by GSVD of the patient-matched GBM and normal aCGH profiles. (a) Plot of the first tumor arraylet describes unsegmented [20,21] chromosomes (black lines), each with copy-number distributions which are approximately centered at zero with relatively large, chromosome-invariant widths. The probes are ordered, and their copy numbers are colored, according to each probe's chromosomal location. (b) Plot of the first most tumor-exclusive probelet, which is also the second most significant probelet in the tumor dataset (Figure S1a), describes the corresponding variation across the patients. The patients are ordered according to each patient's relative copy number in this probelet. These copy numbers significantly correlate with the genomic center where the GBM samples were hybridized at, HMS (red), MSKCC (blue) or multiple locations (gray), with the P-values $<10^{-5}$ (Table 1 and Figure S8a). (c) Raster display of the tumor dataset, with relative gain (red), no change (black) and loss (green) of DNA copy numbers, shows the correspondence between the GBM profiles and the first probelet and tumor arraylet.

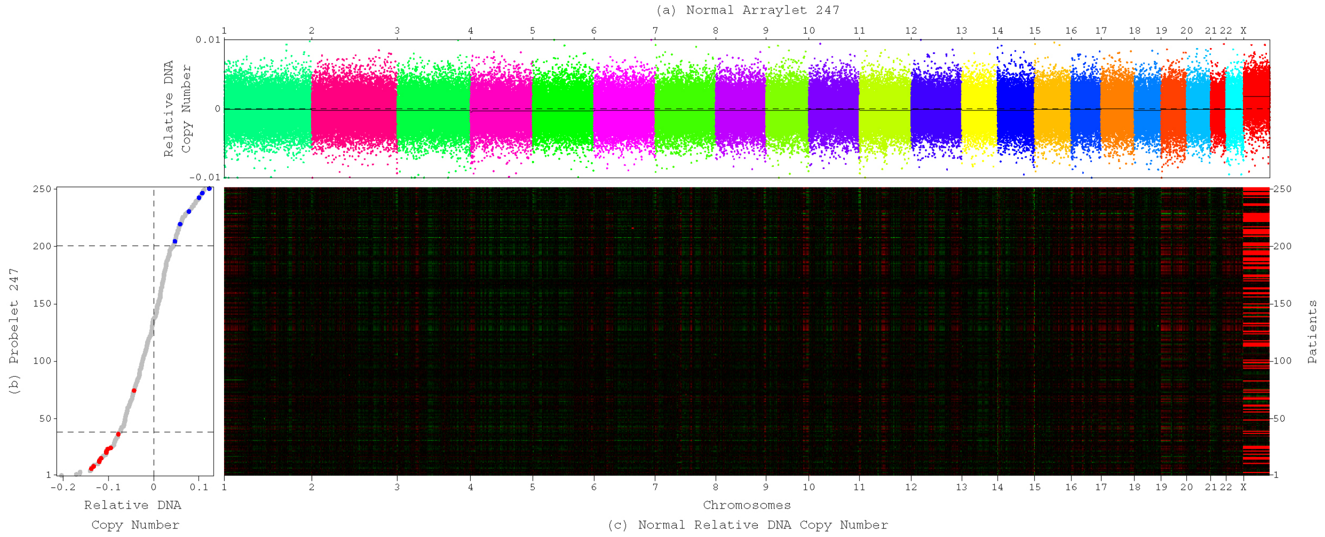

Figure S3. The 247th, normal-exclusive probelet and corresponding normal arraylet uncovered by GSVD. (a) Plot of the 247th normal arraylet describes copy-number distributions which are approximately centered at zero with relatively large, chromosome-invariant widths. The normal probes are ordered, and their copy numbers are colored, according to each probe's chromosomal location. (b) Plot of the 247th probelet describes the corresponding variation across the patients. Copy numbers in this probelet correlate with the date of hybridization of the normal samples, 7.22.2009 (red), 10.8.2009 (blue) or other (gray), with the P-values $<10^{-3}$ (Table 1 and Figure S8b). (c) Raster display of the normal dataset shows the correspondence between the normal profiles and the 247th probelet and normal arraylet.

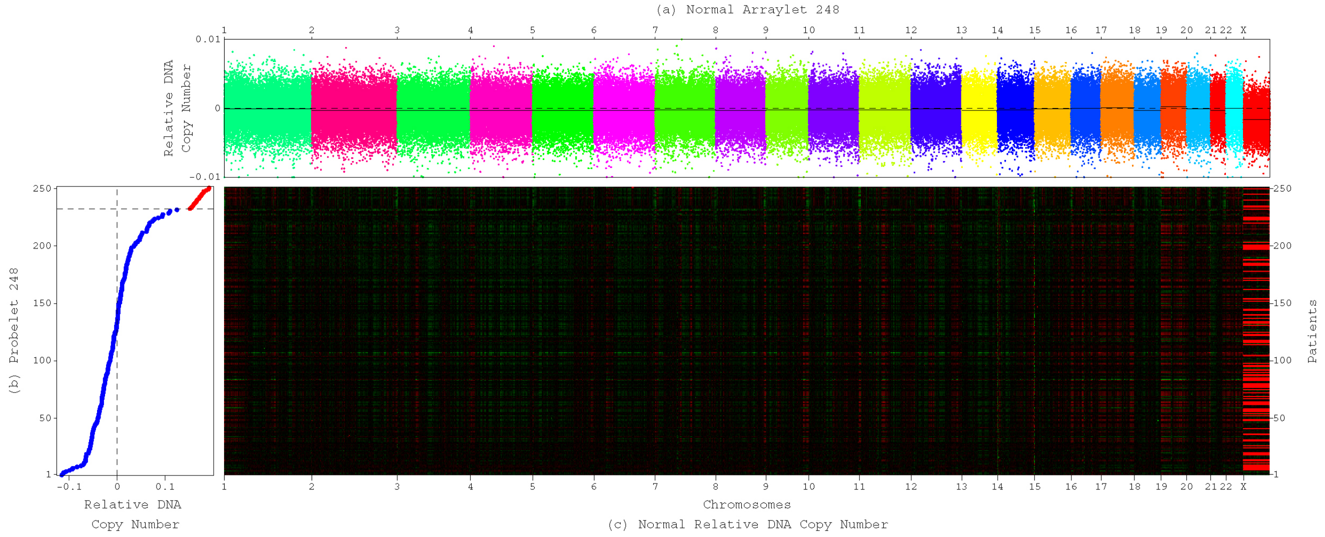

Figure S4. The 248th, normal-exclusive probelet and corresponding normal arraylet uncovered by GSVD. (*a*) Plot of the 248th normal arraylet describes copy-number distributions which are approximately centered at zero with relatively large, chromosome-invariant widths. (*b*) Plot of the 248th probelet describes the corresponding variation across the patients. Copy numbers in this probelet significantly correlate with the tissue batch/hybridization scanner of the normal samples, HMS 8/2331 (red) and other (gray), with the P-values $<10^{-12}$ (Table 1 and Figure S8*c*). (*c*) Raster display of the normal dataset shows the correspondence between the normal profiles and the 248th probelet and normal arraylet.

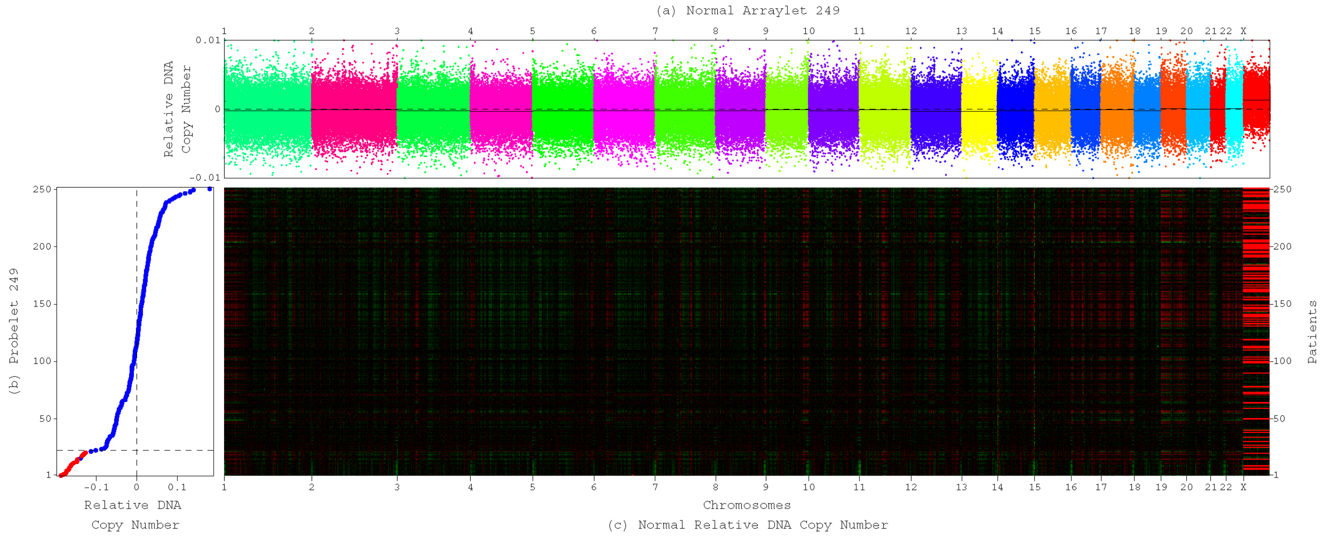

Figure S5. The 249th, normal-exclusive probelet and corresponding normal arraylet uncovered by GSVD. (*a*) Plot of the 249th normal arraylet describes copy-number distributions which are approximately centered at zero with relatively large, chromosome-invariant widths. (*b*) Plot of the 249th probelet describes the corresponding variation across the patients. Copy numbers in this probelet significantly correlate with the tissue batch/hybridization scanner of the normal samples, HMS 8/2331 (red) and other (gray), with the P-values $<10^{-12}$ (Table 1 and Figure S8*d*). (*c*) Raster display of the normal dataset shows the correspondence between the normal profiles and the 249th probelet and normal arraylet.

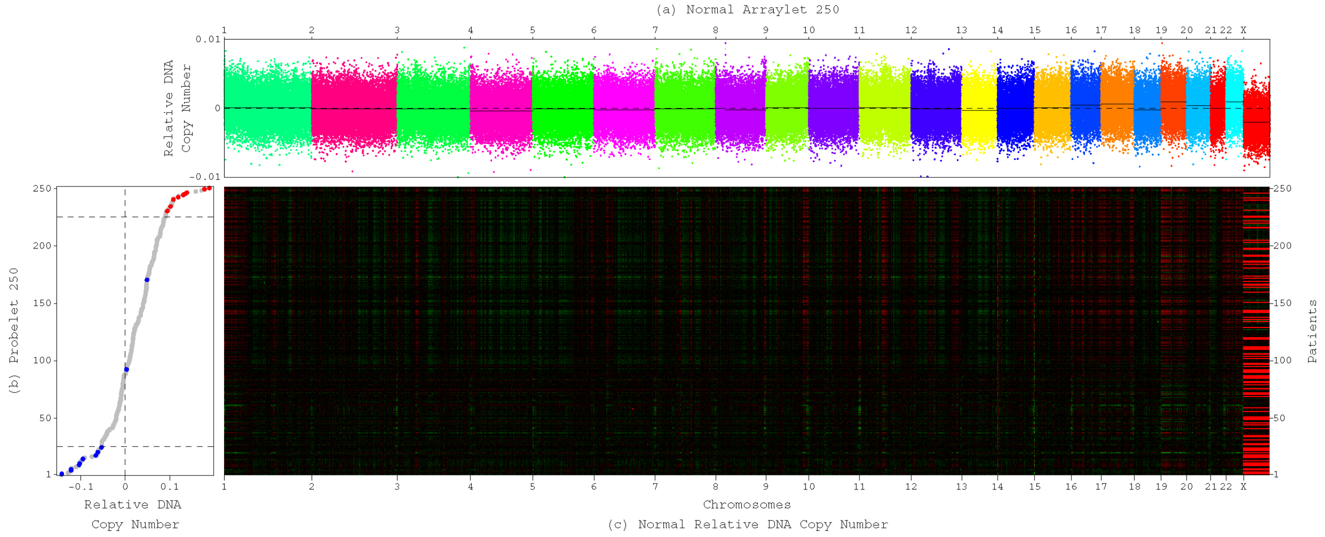

Figure S6. The 250th, normal-exclusive probelet and corresponding normal arraylet uncovered by GSVD. (*a*) Plot of the 250th normal arraylet describes copy-number distributions which are approximately centered at zero with relatively large, chromosome-invariant widths. (*b*) Plot of the 250th probelet describes the corresponding variation across the patients. Copy numbers in this probelet correlate with the date of hybridization of the normal samples, 4.18.2007 (red), 7.22.2009 (blue) or other (gray), with the P-values $<10^{-3}$ (Table 1 and Figure S8*e*). (*c*) Raster display of the normal dataset shows the correspondence between the normal profiles and the 250th probelet and normal arraylet.

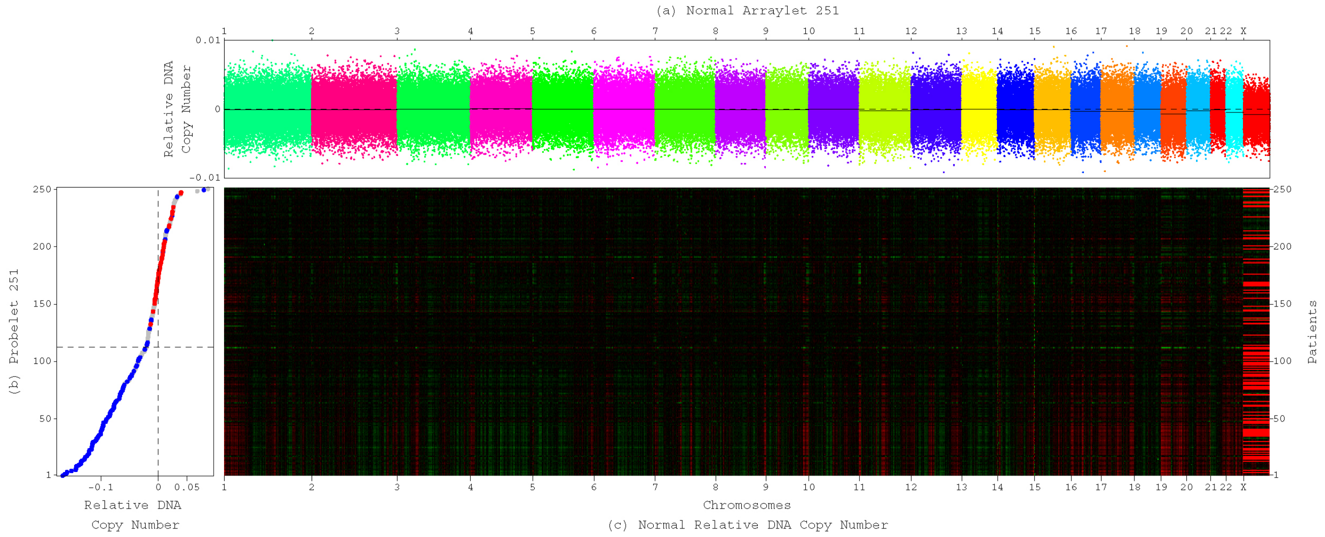

Figure S7. The first most normal-exclusive, i.e., 251st probelet and corresponding normal arraylet uncovered by GSVD. (*a*) Plot of the 251st normal arraylet describes unsegmented [20,21] chromosomes (black lines), each with copy-number distributions which are approximately centered at zero with relatively large, chromosome-invariant widths. (*b*) Plot of the first most normal-exclusive probelet, which is also the most significant probelet in the normal dataset (Figure S1*b*), describes the corresponding variation across the patients. Copy numbers in this probelet significantly correlate with the genomic center where the normal samples were hybridized at, HMS (red), MSKCC (blue) or multiple locations (gray), with the P-values $<10^{-13}$ (Table 1 and Figure S8*f*). (*c*) Raster display of the normal dataset shows the correspondence between the normal profiles and the 251st probelet and normal arraylet.

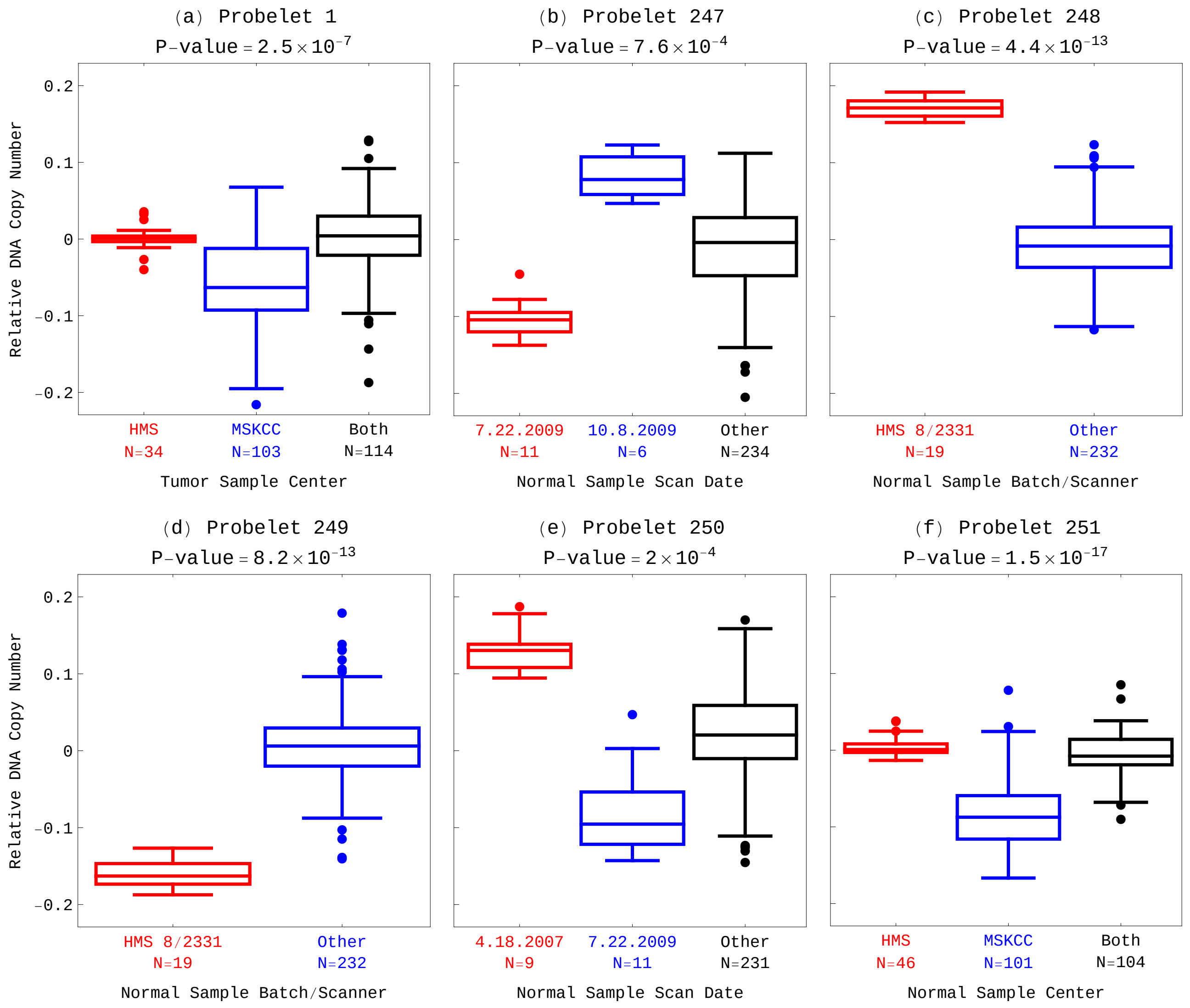

Figure S8 (captions on p. A-6).

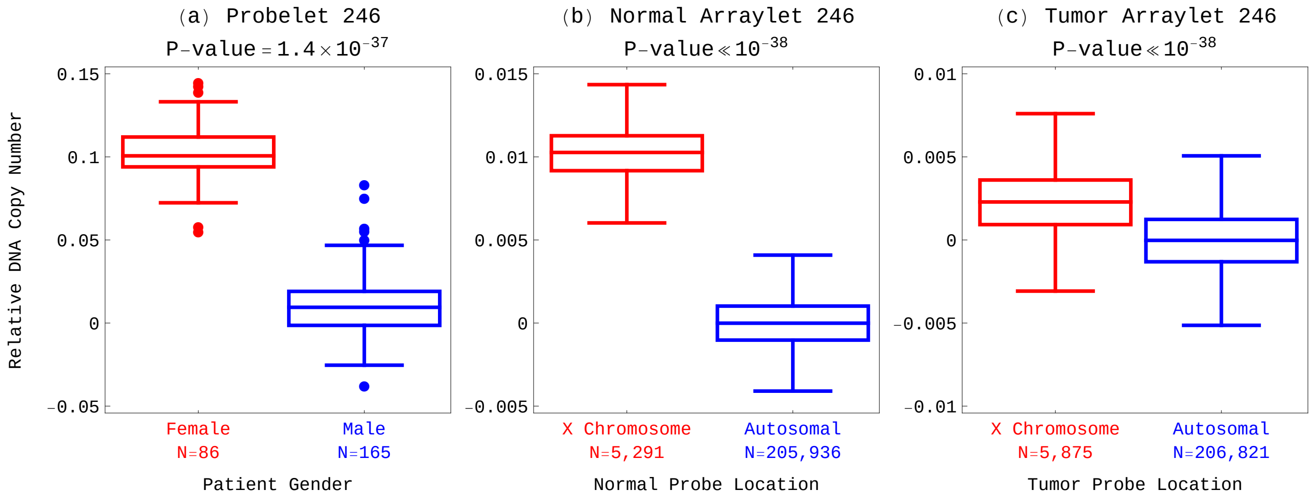

Figure S9 (captions on p. A-6).

Figure S8 (on p. A-5). Differences in copy numbers among the TCGA annotations associated with the significant probelets. Boxplot visualization of the distribution of copy numbers of the (*a*) first, most tumor-exclusive probelet among the associated genomic centers where the GBM samples were hybridized at (Table 1); (*b*) 247th, normal-exclusive probelet among the dates of hybridization of the normal samples; (*c*) 248th, normal-exclusive probelet between the associated tissue batches/hybridization scanners of the normal samples; (*d*) 249th, normal-exclusive probelet between the associated tissue batches/hybridization scanners of the normal samples; (*e*) 250th, normal-exclusive probelet among the dates of hybridization of the normal samples; (*f*) 251st, most normal-exclusive probelet among the associated genomic centers where the normal samples were hybridized at. The Mann-Whitney-Wilcoxon P-values correspond to the two annotations that are associated with largest or smallest relative copy numbers in each probelet.

Figure S9 (on p. A-5). Copy-number distributions of the 246th probelet and the corresponding 246th normal arraylet and 246th tumor arraylet. Boxplot visualization and Mann-Whitney-Wilcoxon P-values of the distribution of copy numbers of the (*a*) 246th probelet, which is approximately common to both the normal and tumor datasets, and is the second most significant in the normal dataset (Figure S1*b*), between the gender annotations (Table 1); (*b*) 246th normal arraylet between the autosomal and X chromosome normal probes; (*c*) 246th tumor arraylet between the autosomal and X chromosome tumor probes.

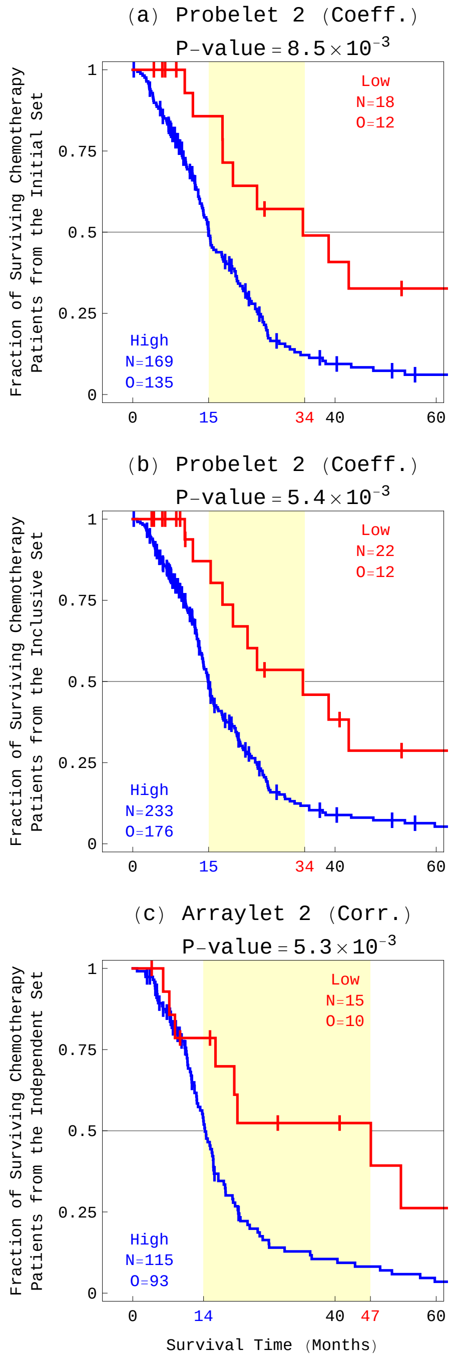

Figure S10. Kaplan-Meier (KM) survival analyses of only the chemotherapy patients from the three sets classified by GSVD.

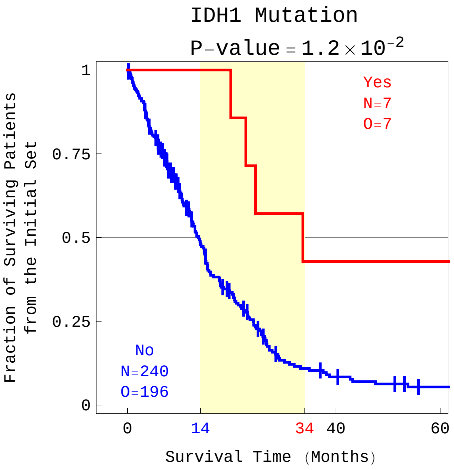

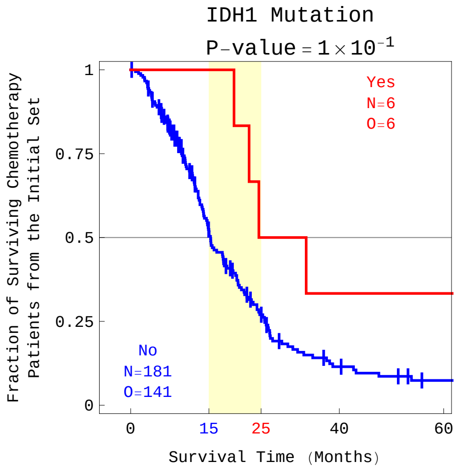

Figure S11 (left). KM survival analysis of the initial set of 251 patients classified by a mutation in the gene *IDH1*.

Figure S12 (right). KM survival analysis of only the chemotherapy patients in the initial set, classified by a mutation in *IDH1*.

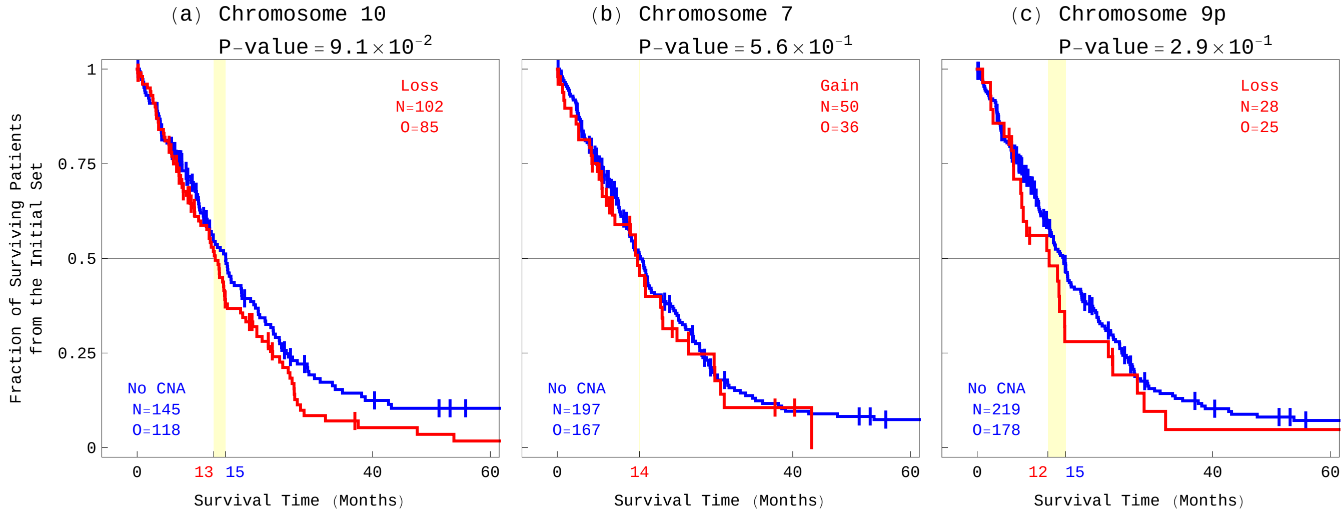

Figure S13. KM survival analyses of the initial set of 251 patients classified by GBM-associated chromosome number changes. (*a*) Analysis of the 247 patients with TCGA annotations in the initial set of 251 patients, classified by number changes in chromosome 10, shows almost overlapping Kaplan-Meier (KM) [36] curves with a KM median survival time difference of ∼2 months, and a corresponding log-rank test *P*-value $\sim 10^{-1}$, meaning that chromosome 10 loss, frequently observed in GBM, is a poor predictor of GBM patients' survival. (*b*) KM survival analysis of the 247 patients classified by number changes in chromosome 7 shows almost overlapping KM curves with a KM median survival time difference of <one month, and a corresponding log-rank test *P*-value $>5\times10^{-1}$, meaning that chromosome 7 gain is a poor predictor of GBM survival. (*c*) KM survival analysis of the 247 patients classified by number changes in chromosome 9p shows a KM median survival time difference of ∼3 months, and a log-rank test *P*-value $>10^{-1}$, meaning that chromosome 9p loss is a poor predictor of GBM survival.

Figure S14 (on p. A-8). KM survival analyses of the initial set of 251 patients classified by copy number changes in selected segments containing GBM-associated genes or genes previously unrecognized in GBM. In the KM survival analyses of the groups of patients with either a CNA or no CNA in either one of the 130 segments identified by the global pattern, i.e., the second tumor-exclusive arraylet (Dataset S3), log-rank test *P*-values $<5\times10^{-2}$ are calculated for only 12 of the classifications. Of these, only six correspond to a KM median survival time difference that is $\gtrsim$5 months, approximately a third of the ∼16 months difference observed for the GSVD classification. One of these segments contains the genes *TLK2* and *METTL2A*, previously unrecognized in GBM. The KM median survival time we calculate for the 56 patients with *TLK2* amplification is ∼5 months longer than that for the remaining patients. This suggests that drug-targeting the kinase and/or the methyltransferase-like protein that *TLK2* and *METTL2A* encode, respectively, may affect not only the pathogenesis but also the prognosis of GBM.

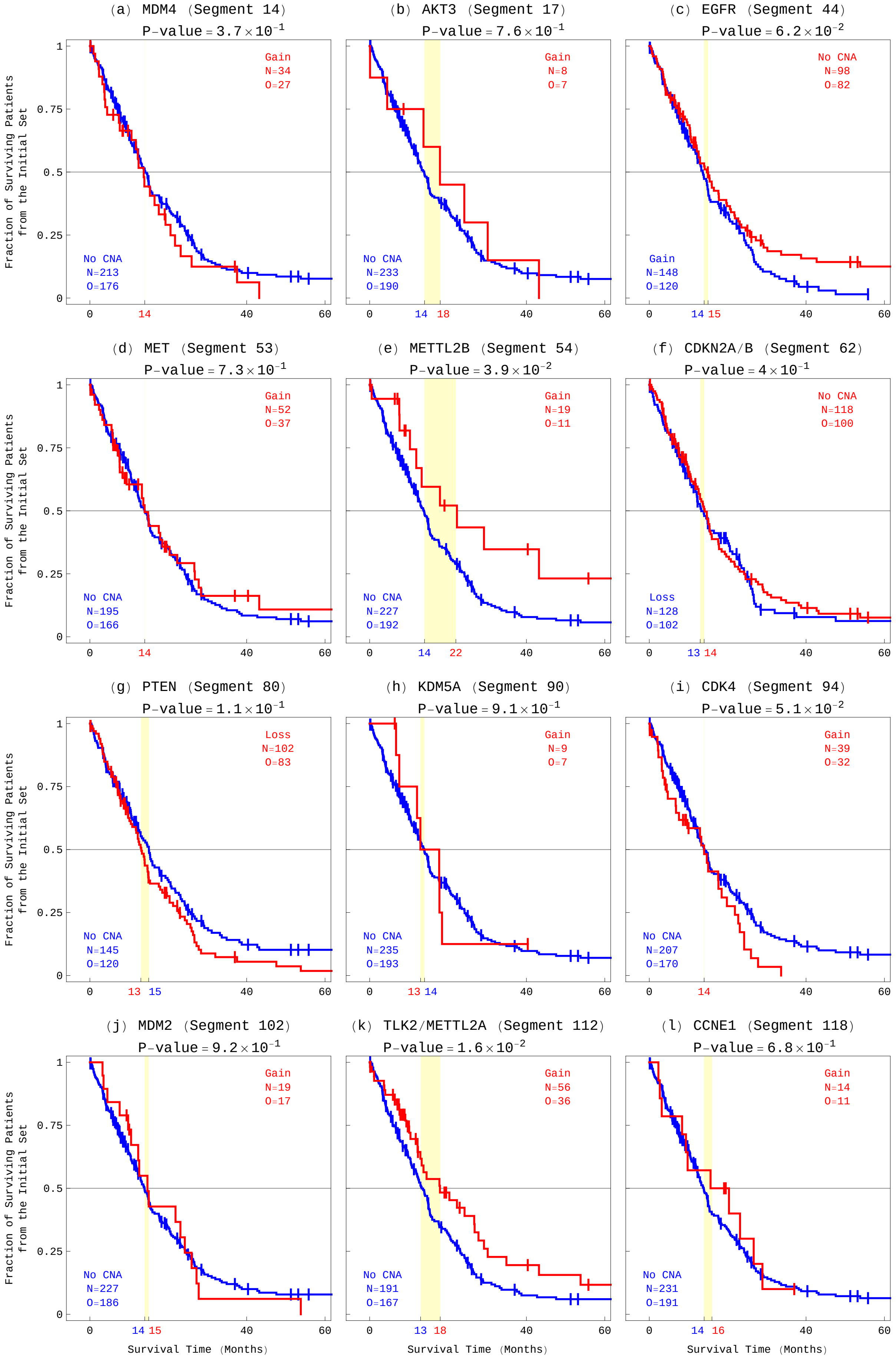

Figure S14 (captions on p. A-7).

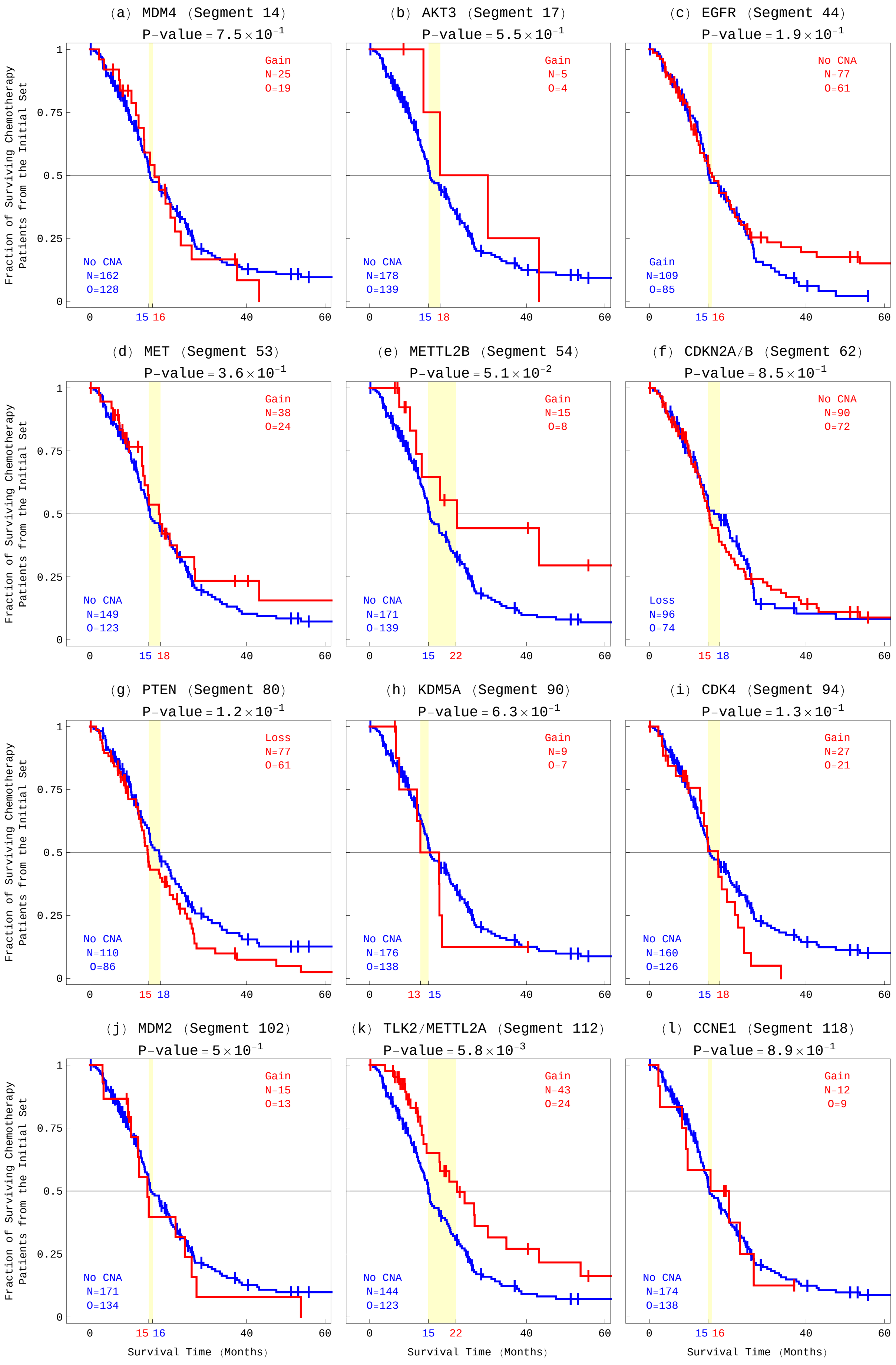

Figure S15. KM survival analyses of only the chemotherapy patients in the initial set of 251 patients classified by copy number changes in selected segments.

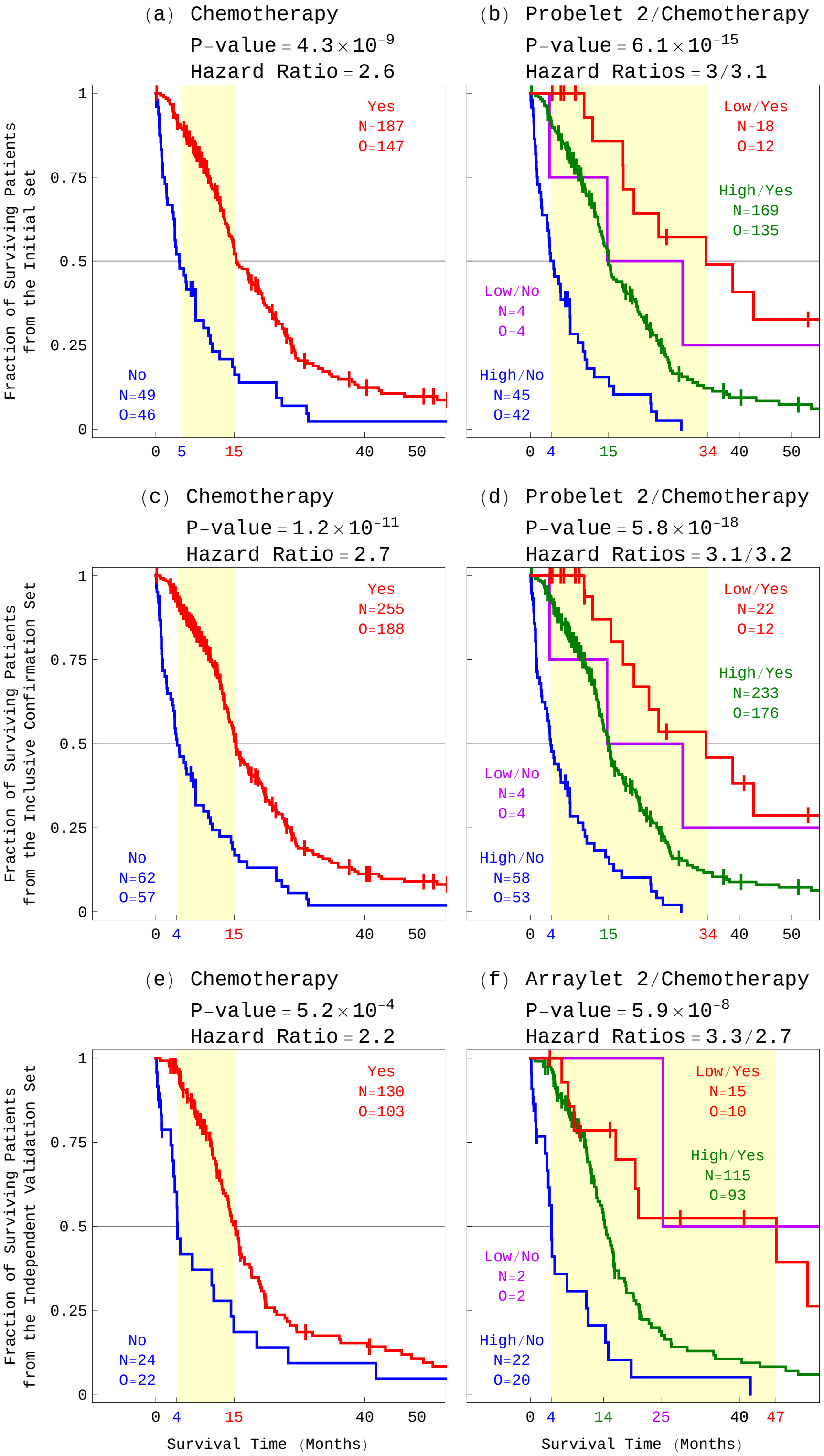

Figure S16. Survival analyses of the patients from the three sets classified by chemotherapy alone or GSVD and chemotherapy both. (*a*) KM and Cox survival analyses of the 236 patients with TCGA chemotherapy annotations in the initial set of 251 patients, classified by chemotherapy, show that lack of chemotherapy, with a KM median survival time difference of ∼10 months and a univariate hazard ratio of 2.6 (Table S2), confers more than twice the hazard of chemotherapy. (*b*) Survival analyses of the 236 patients classified by both GSVD and chemotherapy, show similar multivariate Cox hazard ratios, of 3 and 3.1, respectively. This means that GSVD and chemotherapy are independent prognostic predictors. With a KM median survival time difference of ∼30 months, GSVD and chemotherapy combined make a better predictor than chemotherapy alone. (*c*) Survival analyses of the 317 patients with TCGA chemotherapy annotations in the inclusive confirmation set of 344 patients, classified by chemotherapy, show a KM median survival time difference of ∼11 months and a univariate hazard ratio of 2.7, and confirm the survival analyses of the initial set of 251 patients. (*d*) Survival analyses of the 317 patients classified by both GSVD and chemotherapy show similar multivariate Cox hazard ratios, of 3.1 and 3.2, and a KM median survival time difference of ∼30 months, with the corresponding log-rank test P-value $<10^{-17}$. This confirms that the prognostic contribution of GSVD is independent of chemotherapy, and that combined with chemotherapy, GSVD makes a better predictor than chemotherapy alone. (*e*) Survival analyses of the 154 patients with TCGA chemotherapy annotations in the independent validation set of 184 patients, classified by chemotherapy, show a KM median survival time difference of ∼11 months and a univariate hazard ratio of 2.2, and validate the survival analyses of the initial set of 251 patients. (*f*) Survival analyses of the 154 patients classified by both GSVD and chemotherapy, show similar multivariate Cox hazard ratios, of 3.3 and 2.7, and a KM median survival time difference of ∼43 months. This validates that the prognostic contribution of GSVD is independent of chemotherapy, and that combined with chemotherapy, GSVD makes a better predictor than chemotherapy alone, also for patients with measured GBM aCGH profiles in the absence of matched normal profiles.
