# Supplementary material for: GSVD Comparison of Patient-Matched Normal and Tumor aCGH Profiles Reveals Global Copy-Number Alterations Predicting Glioblastoma Multiforme Survival
Source: PLoS One. 2012 Jan 23;7(1):e30098. doi: 10.1371/journal.pone.0030098 (PMC3264559; doi:10.1371/journal.pone.0030098)
Supplement: Mathematica Notebook S2 — Generalized singular value decomposition (GSVD) of the TCGA patient-matched tumor and normal aCGH profiles. A PDF format file, readable by Adobe Acrobat Reader. (PDF) [file pone.0030098.s003.pdf]

(* © Benjamin O. Alpert and Orly Alter 2012, All Rights Reserved *)

```
(* GSVD of Patient-Matched Tumor and Normal aCGH Datasets *)

(* Initialize *)

Clear["Global`*"]

(* Define Path to Datasets *)

path = "Desktop/TCGA/";

(* Read aCGH Tumor and Normal Datasets *)

stream = path <> "251_Tumor.txt";
tumorData = Import[stream, "Table"];
stream = path <> "251_Normal.txt";
normalData = Import[stream, "Table"];

(* Extract Probe IDs and Array Names *)

tumorArrayNames = Drop[tumorData[[1]], 3];
tumorProbeID = Drop[tumorData[[All, 1]], 1];
tumorProbeLoc = Drop[tumorData[[All, 2 ;; 3]], 1];
tumorData = Drop[tumorData, 1, 3];
normalArrayNames = Drop[normalData[[1]], 3];
normalProbeID = Drop[normalData[[All, 1]], 1];
normalProbeLoc = Drop[normalData[[All, 2 ;; 3]], 1];
normalData = Drop[normalData, 1, 3];

{tumorProbes, arrays} = Dimensions[tumorData]
{normalProbes, arrays} = Dimensions[normalData]

{212 696, 251}

{211 227, 251}

(* Compute GSVD *)

(* Compute the QR Decomposition of the Appended Tumor and Normal Datasets *)

data = Join[tumorData, normalData];
{q, r} = QRDecomposition[data];
q = Transpose[q];

(* Compute the SVD of the Tumor Block of Q *)

q1 = q[[1 ;; tumorProbes]];
{u1, w1, vt1} = SingularValueDecomposition[q1, arrays];
vt1 = Transpose[vt1].r;
d1 = DiagonalMatrix[Diagonal[Sqrt[vt1.Transpose[vt1]]]];
Do[vt1[[a]] = vt1[[a]] / d1[[a, a]], {a, 1, arrays}];
d1 = w1.d1;
probelets = vt1;
tumorArraylets = u1;

(* Compute the SVD of the Normal Block of Q *)

q2 = q[[tumorProbes + 1 ;; normalProbes + tumorProbes]];
{u2, w2, vt2} = SingularValueDecomposition[q2, arrays];
vt2 = Transpose[vt2] . r;
d2 = DiagonalMatrix[Diagonal[Sqrt[vt2 . Transpose[vt2]]]];
Do[vt2[[a]] = vt2[[a]] / d2[[a, a]], {a, 1, arrays}];
```

```
(* Order the SVD of the Normal Block of Q by Ascending Singular Values *)

vt2 = Transpose[Take[
        Transpose[Sort[
          Transpose[
           Join[{Diagonal[w2]}, Transpose[vt2]]
           ]]]][[2 ;; arrays + 1]]];
d2 = DiagonalMatrix[
      Sort[Diagonal[w2]] *
       Transpose[Sort[
          Transpose[
           Join[{Diagonal[w2]}, {Diagonal[d2]}]
           ]]][[2]]];
normalArraylets =
      Take[Transpose[
          Sort[Transpose[
            Join[{Diagonal[w2]}, u2]
            ]]][[2 ;; normalProbes + 1]]];
signs = DiagonalMatrix[Diagonal[vt2.Transpose[vt1]]];
vt2 = signs.vt2;
normalArraylets = normalArraylets.signs;

(* Define the Sign of the Second Probelet and Corresponding Tumor and Normal Arraylets *)

probelets[[2]] = -probelets[[2]];
tumorArraylets[[All, 2]] = -tumorArraylets[[All, 2]];
normalArraylets[[All, 2]] = -normalArraylets[[All, 2]];



(* Display Significant Probelets and Corresponding Tumor and Normal Arraylets *)

rasterContrast = 2;
resolution = 3600;

(* Create Raster Displays of Tumor and Normal Datasets *)

step = 100;

binnedTumorData = Table[
   Mean[tumorData[[a ;; a + step - 1]]], {a, 1, Floor[tumorProbes, step], step}];
{binnedRows, arrays} = Dimensions[binnedTumorData];
raster = Table[
   adjustedValue = binnedTumorData[[a, b]];
   If[adjustedValue > 0,
    {adjustedValue, 0, 0},
    {0, adjustedValue, 0}],
   {a, 1, binnedRows}, {b, 1, arrays}];
raster[[All, All, 2]] = -raster[[All, All, 2]];
rawTumorRaster = raster;

binnedNormalData = Table[
   Mean[normalData[[a ;; a + step - 1]]], {a, 1, Floor[normalProbes, step], step}];
{binnedRows, arrays} = Dimensions[binnedNormalData];
raster = Table[
   adjustedValue = binnedNormalData[[a, b]];
   If[adjustedValue > 0,
    {adjustedValue, 0, 0},
    {0, adjustedValue, 0}],
   {a, 1, binnedRows}, {b, 1, arrays}];
raster[[All, All, 2]] = -raster[[All, All, 2]];
rawNormalRaster = raster;
```

```
(* Sort Selected Probelet and Arraylet *)

sortData[probeletAnnotationColors_, raster_, sample_] := {
  c = .5;
  sortProbelets = Sort[probelets[[pattern]]];
  sortProbeletAnnotationColors = probeletAnnotationColors[[Ordering[probelets[[pattern]]]]];
  sortRaster = Transpose[raster[[All, Ordering[probelets[[pattern]]]]]];
  d = .5;
  If[sample == "Tumor", {
    arraylet = tumorArraylets[[All, pattern]];
    chromosomeDefinitions = tumorChromosomeDefinitions;
    chromosomeBounds = tumorChromosomeBounds},
   {arraylet = normalArraylets[[All, pattern]];
    chromosomeDefinitions = normalChromosomeDefinitions;
    chromosomeBounds = normalChromosomeBounds}];
 }

(* Define Label Coordinates *)

figureVariables[arraylet_, sortProbelets_, arrayletRange_] := {
  genes = Dimensions[arraylet][[1]];
  range =
   {{Min[sortProbelets] - .01, 0}, {Max[sortProbelets] + .01, Dimensions[sortProbelets][[1]]}};
  arrayletsXLabels = {ToString[arrayletRange[[1]]], "0", ToString[arrayletRange[[2]]]};
  xScaleFactor = genes * .15 / (range[[2, 1]] - range[[1, 1]]);
  range[[All, 1]] = range[[All, 1]] * xScaleFactor;
  xOffset = range[[2, 1]] + genes * .01;
  ylabels = {1, 50, 100, 150, 200, 250};
  dotsScaleFactor = 5000 + 1000;
  yOffset = 320;
  dotsOffset = yOffset - Mean[arrayletRange] * dotsScaleFactor;
  numChromosomes = 23;
  labelSize = 10;
  ticksLabelSize = 8;
 }

(* Define Chromosome Colors *)

colorDefinitions = Chop[Drop[Flatten[Table[{ColorConvert[Hue[a / 24], RGBColor],
       ColorConvert[Hue[(a + 12) / 24], RGBColor]}, {a, 10, -1, -1}]], {24}]];

(* Read Segmentation Data *)

stream = path <> "251_Segments.txt";
segments = Drop[Import[stream, "Table"], 1];
segmentsSplit = SplitBy[segments, First];

(* Match Tumor and Normal Probes to Chromosome Location *)

tumorChromosomeDefinitions = Table[Position[tumorProbeLoc, a][[All, 1]], {a, 23}];
tumorChromosomeBounds = Table[{
    Min[tumorChromosomeDefinitions[[a]]], Max[tumorChromosomeDefinitions[[a]]]}, {a, 23}];

normalChromosomeDefinitions = Table[Position[normalProbeLoc, a][[All, 1]], {a, 23}];
normalChromosomeBounds = Table[{
    Min[normalChromosomeDefinitions[[a]]], Max[normalChromosomeDefinitions[[a]]]}, {a, 23}];

(* Define Segments Line Display *)

segmentsLines[segmentsSplit_, arrayletRange_, dotsScaleFactor_, dotsOffset_] := {
  patternPos = Position[segmentsSplit[[All, 1, 1]], pattern][[1]];
  Graphics[Table[
    Line[{{segmentsSplit[[patternPos, a, 6]][[1]],
       Clip[segmentsSplit[[patternPos, a, 9]][[1]], {arrayletRange[[1]], arrayletRange[[2]]}] *
         dotsScaleFactor + dotsOffset}, {segmentsSplit[[patternPos, a, 7]][[1]],
       Clip[segmentsSplit[[patternPos, a, 9]][[1]], {arrayletRange[[1]], arrayletRange[[2]]}] *
         dotsScaleFactor + dotsOffset}}],
    {a, 1, Dimensions[segmentsSplit[[patternPos]][[1]]][[1]]}]]
 }
```

```
(* Define Arraylet Plot Display *)

arrayletsFigure[chromosomes_, dotsScaleFactor_, arraylet_,
  chromosomeDefinitions_, numChromosomes_, arrayletRange_, dotsOffset_,
  genes_, chromosomeBounds_, range_, sample_, arrayletsXLabels_, format_] := {
  Table[
   Graphics[{colorDefinitions[[a]], PointSize[1 / dotsScaleFactor],
     Point[Table[{c++, arraylet[[chromosomeDefinitions[[a, b]]]]},
       {b, Dimensions[chromosomeDefinitions[[a]]][[1]]}]]}], {a, numChromosomes}],
  Graphics[Line[{{0, arrayletRange[[1]] * dotsScaleFactor + dotsOffset},
     {genes, arrayletRange[[1]] * dotsScaleFactor + dotsOffset},
     {genes, arrayletRange[[2]] * dotsScaleFactor + dotsOffset},
     {0, arrayletRange[[2]] * dotsScaleFactor + dotsOffset},
     {0, arrayletRange[[1]] * dotsScaleFactor + dotsOffset}}]],
  Graphics[{Dashed, Line[{{0, dotsOffset}, {genes, dotsOffset}}]}],
  Table[Graphics[{
     Text[Style[chromosomes[[a]], ticksLabelSize], {chromosomeBounds[[a, 1]],
       (arrayletRange[[2]] + .0005) * dotsScaleFactor + dotsOffset}, {0, -1}],
     Line[{{chromosomeBounds[[a, 1]], arrayletRange[[2]] * dotsScaleFactor + dotsOffset},
       {chromosomeBounds[[a, 1]], (arrayletRange[[2]] + .0003) * dotsScaleFactor + dotsOffset}}],
     Text[Style[chromosomes[[a]], ticksLabelSize],
      {chromosomeBounds[[a, 1]], range[[1, 2]] - 5}, {0, 1}],
     Line[{{chromosomeBounds[[a, 1]], range[[1, 2]] + 0},
       {chromosomeBounds[[a, 1]], range[[1, 2]] - 4}}]}], {a, 1, numChromosomes}],
  Graphics[{Text[Style[ColumnForm[{"Relative DNA", "Copy Number"}, Center], labelSize],
     {-genes * .045, yOffset}, {0, 0}, {0, 1}],
    If[Length[format] == 1,
     Text[Style["(d)  " <> sample <> "  Arraylet  " <> ToString[pattern], labelSize],
      {genes / 2, (arrayletRange[[2]] + .0045) * dotsScaleFactor + dotsOffset}, {0, 0}],
     Text[Style["(a)  " <> sample <> "  Arraylet  " <> ToString[pattern], labelSize],
      {genes / 2, (arrayletRange[[2]] + .0045) * dotsScaleFactor + dotsOffset}, {0, 0}]],
    Text[Style["Chromosomes" , labelSize], {genes / 2, -18}, {0, 1}],
    If[Length[format] == 1,
     Text[Style["(f)  " <> sample <> "  Relative DNA Copy Number", labelSize], {genes / 2, -35}, {0, 1}],
     Text[
      Style["(c)  " <> sample <> "  Relative DNA Copy Number", labelSize], {genes / 2, -35}, {0, 1}]]}],
  Table[Graphics[{Text[Style[arrayletsXLabels[[a]], ticksLabelSize],
      {-genes * .003, ToExpression[arrayletsXLabels[[a]]] * dotsScaleFactor + dotsOffset}, {1, 0}]}],
   {a, 1, Dimensions[arrayletsXLabels][[1]]}]
 }

(* Define Genes Arrow Display *)

arrowLabels[genes_, arraylet_, lociList_, labelSize_] := {
  Table[{
    Graphics[{Arrowheads[.006], Arrow[{
        {.02 * genes * Sin[lociList[[a, 4]] Degree] + lociList[[a, 2]],
         17 * Cos[lociList[[a, 4]] Degree] + arraylet[[lociList[[a, 2]]]]},
        {lociList[[a, 2]], arraylet[[lociList[[a, 2]]]]}},
       {0, .002 * genes * Sin[lociList[[a, 4]] Degree] ^2 + 4 * Cos[lociList[[a, 4]] Degree] ^2}]}],
    Graphics[{lociList[[a, 3]], Style[Text[lociList[[a, 1]],
        {.02 * genes * Sin[lociList[[a, 4]] Degree] + lociList[[a, 2]],
         19 * Cos[lociList[[a, 4]] Degree] + arraylet[[lociList[[a, 2]]]]},
        {-Sin[lociList[[a, 4]] Degree], -Cos[lociList[[a, 4]] Degree]}],
       labelSize]}]}, {a, Dimensions[lociList][[1]]}]
 }

(* Define Genes *)

lociList = {{"MDM4", 14 432, Black, 315}, {"AKT3", 17 428, Black, 45}, {"EGFR ", 92 730, Black, 250},
   {"MET", 97 150, Black, 0}, {" METTL2B", 98 060, Red, 75}, {"CDKN2A/B ", 112 713, Black, 270},
   {" PTEN", 125 990, Black, 100}, {"CDK4", 143 785, Black, 320}, {" MDM2", 144 525, Black, 90},
   {"KDM5A", 139 012, Red, 330}, {"TLK2/METTL2A", 182 766, Red, 330}, {"CCNE1", 192 313, Red, 45}};
```

```
(* Define Probelet Plot Display *)

probeletFigure[sortProbeletAnnotationColors_, sortProbelets_, xScaleFactor_,
  range_, cutoff_, probeletXLabels_, ticksLabelSize_, genes_, pattern_, format_] := {
  sortPoints = Table[{sortProbelets[[a]] * xScaleFactor - xOffset, d++},
    {a, Dimensions[sortProbelets][[1]]}];
  redPos = Flatten[Position[sortProbeletAnnotationColors, Red]];
  bluePos = Flatten[Position[sortProbeletAnnotationColors, Blue]];
  grayPos = Flatten[Complement[Range[Length[sortProbelets]], bluePos, redPos]];
  Graphics[{
    Table[{sortProbeletAnnotationColors[[a]], PointSize[.003], Point[sortPoints[[a]]]},
     {a, Join[grayPos, bluePos, redPos]}],
    EdgeForm[Black], Opacity[0], Rectangle[{range[[1, 1]] - xOffset, range[[1, 2]]},
     {range[[2, 1]] - xOffset, range[[2, 2]] + 1}],
    Opacity[1], Dashed, Black,
    Table[Line[{{range[[1, 1]] - xOffset, .5 + cutoff[[1, a]]},
       {range[[2, 1]] - xOffset, .5 + cutoff[[1, a]]}}], {a, Dimensions[cutoff[[1]]][[1]]}],
    Table[Line[{{-xOffset + cutoff[[2, a]] * xScaleFactor, range[[1, 2]]}, {-xOffset +
         cutoff[[2, a]] * xScaleFactor, range[[2, 2]]}}], {a, Dimensions[cutoff[[2]]][[1]]}],
    Table[{
      Text[Style[Chop[probeletXLabels[[a]]], ticksLabelSize],
       {probeletXLabels[[a]] * xScaleFactor - xOffset, range[[1, 2]] - 5}, {0, 1}],
      Line[{{probeletXLabels[[a]] * xScaleFactor - xOffset, range[[1, 2]] - 4},
        {probeletXLabels[[a]] * xScaleFactor - xOffset, range[[1, 2]] + 0}}]},
     {a, 1, Dimensions[probeletXLabels][[1]]}],
    Text[Style["Relative DNA", labelSize], {Mean[range[[All, 1]]] - xOffset - 500, -18}, {0, 1}],
    Text[Style["Copy Number", labelSize], {Mean[range[[All, 1]]] - xOffset, -35}, {0, 1}],
    Table[{
      Text[Style[ylabels[[a]], ticksLabelSize],
       {range[[1, 1]] - genes * .005 - xOffset, ylabels[[a]]}, {1, 0}],
      Line[{{range[[1, 1]] - genes * .003 - xOffset, ylabels[[a]]},
        {range[[1, 1]] - xOffset, ylabels[[a]]}}],
      Text[Style[ylabels[[a]], ticksLabelSize], {genes * 1.003, ylabels[[a]]}, {-1, 0}],
      Line[{{genes, ylabels[[a]]}, {genes * 1.003, ylabels[[a]]}}]}
     , {a, 1, Dimensions[ylabels][[1]]}],
    Text[Style["Patients", labelSize], {genes * 1.04, Mean[range[[All, 2]]]}, {0, 0}, {0, 1}],
    If[Length[format] == 1, Style[Text["(e) Probelet " <> ToString[pattern],
       {range[[1, 1]] - xOffset - genes * .035, Mean[range[[All, 2]]]}, {0, -1}, {0, 1}], labelSize],
     Style[Text["(b) Probelet " <> ToString[pattern], {range[[1, 1]] - xOffset - genes * .035,
        Mean[range[[All, 2]]]}, {0, -1}, {0, 1}], labelSize]]}]
 }

(* Define Probelet and Arraylet Display *)

compositeFigure[pattern_, sample_, probeletAnnotationColors_,
  raster_, lociList_, cutoff_, arrayletRange_, probeletXLabels_, format_] := {
  chromosomes = Join[Range[1, 22], {"X"}];
  sortData[probeletAnnotationColors, raster, sample];
  figureVariables[arraylet, sortProbelets, arrayletRange];
  arraylet = Clip[arraylet, arrayletRange] * dotsScaleFactor + dotsOffset;
  gRaster = Graphics[Raster[sortRaster, {{0, .5}, {genes, 251.5}}, ColorFunction → RGBColor]];
  gArraylet =
   arrayletsFigure[chromosomes, dotsScaleFactor, arraylet, chromosomeDefinitions, numChromosomes,
    arrayletRange, dotsOffset, genes, chromosomeBounds, range, sample, arrayletsXLabels, format];
  gSegments = segmentsLines[segmentsSplit, arrayletRange, dotsScaleFactor, dotsOffset];
  gArrows = arrowLabels[genes, arraylet, lociList, labelSize];
  gProbelets = probeletFigure[sortProbeletAnnotationColors, sortProbelets,
    xScaleFactor, range, cutoff, probeletXLabels, ticksLabelSize, genes, pattern, format];
  Show[{gRaster, gArraylet, gSegments, gArrows, gProbelets},
   AspectRatio → .4,
   ImageSize → 1000,
   BaseStyle → {FontFamily → "Courier"},
   PlotRange → {All, {-50, 420}}]
 }
```

```
(* Read TCGA Patient Annotations *)

stream = path <> "251_Patients.txt";
arrayAnnotations = Import[stream, "Table"];
headers = arrayAnnotations[[1]];
arrayAnnotations = Sort[Drop[Import[stream, "List"], 1]];
arrayAnnotations =
  Table[StringSplit[arrayAnnotations[[a]], "\t"], {a, 1, Dimensions[arrayAnnotations][[1]]}];
arrayAnnotations[[All, 12 ;; 20]] = ToExpression[arrayAnnotations[[All, 12 ;; 20]]];
arrayAnnotations[[All, 2 ;; 4]] = ToExpression[arrayAnnotations[[All, 2 ;; 4]]];
{rows, columns} = Dimensions[arrayAnnotations];

(* Create Second Probelet and Tumor Arraylet Display *)

pattern = 2;
colors = Table[Blue, {arrays}];
colors[[Ordering[probelets[[pattern]]][[2 ;; 24]]]] = Red;
colors[[Ordering[probelets[[pattern]]][[1]]]] = RGBColor[0.75, 0.75, 0.75];
condition = "Tumor";

t2 = compositeFigure[pattern, condition, colors, rawTumorRaster,
     lociList, {{25.5}, {-0.02, 0.02}}, {-0.01, 0.01}, {-0.02, 0.02, 0.1}, {}][[1]];

(* Create 246th Probelet and Normal Arraylet Display *)

genderColumn = Flatten[Position[headers, "copy-number.gender"]];
colors = ReplaceAll[arrayAnnotations[[All, genderColumn[[1]]]], {"MALE" → Blue, "FEMALE" → Red}];
pattern = 246;
condition = "Normal";

n246 = compositeFigure[pattern, condition, colors,
     rasterContrast * rawNormalRaster, {}, {{165.5}, {0}}, {-.005, .015}, {0, 0.05, 0.1}, {1}][[1]];
```

```
(* Create Second and 246th Probelets and Corresponding Arraylets Display *)
```

```
fig2 = GraphicsGrid[{{t2}, {n246}}, Spacings → 10, ImageSize → 1000]
```

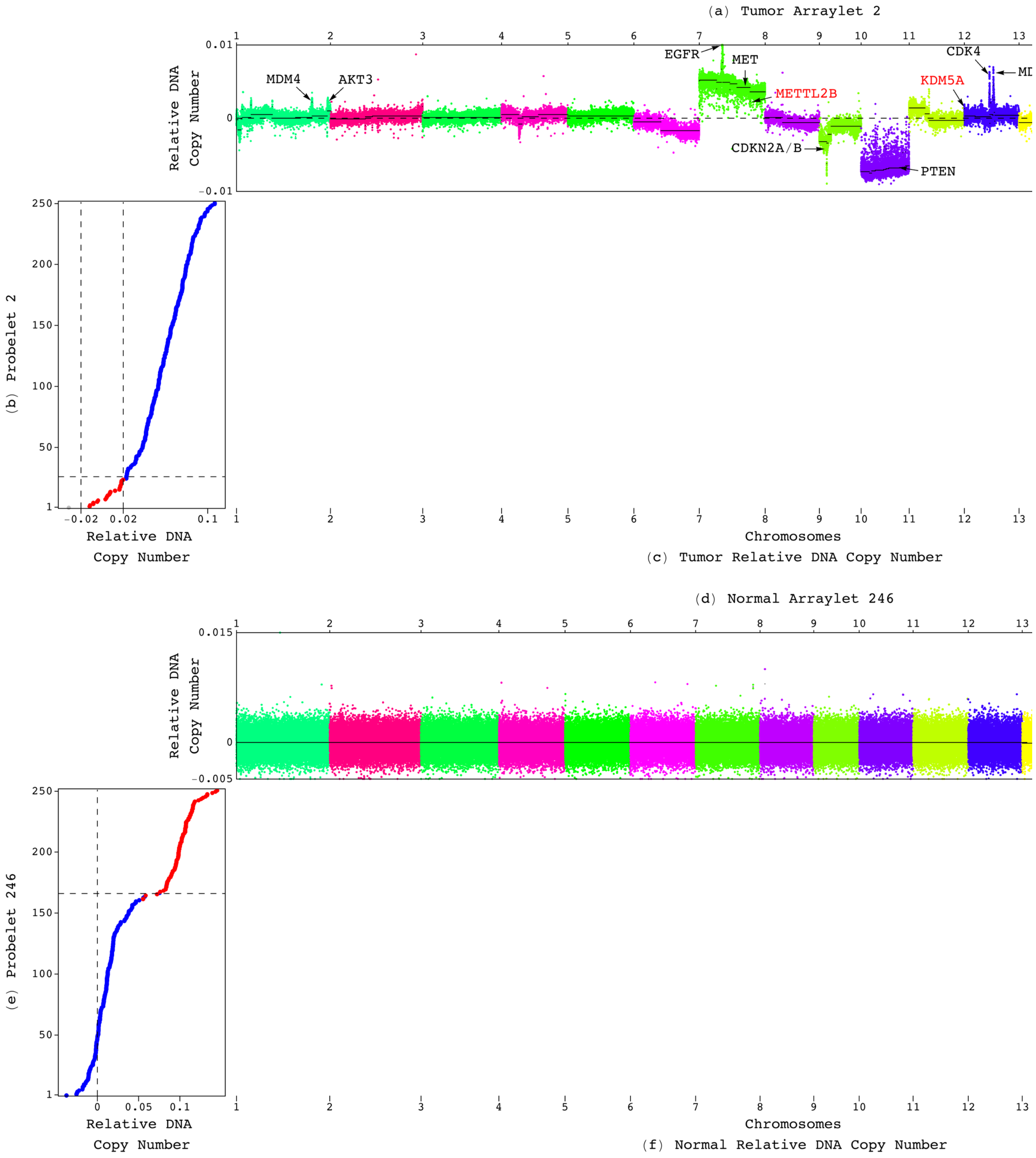


```
Export[path <> "Figure_2.pdf", fig2, "PDF", ImageSize → 1000, ImageResolution → resolution];
```

```
(* Create First Probelet and Tumor Arraylet Display *)

tumorCenterColumn = Flatten[Position[headers, "tumor.center"]];
pattern = 1;
condition = "Tumor";
colors = ReplaceAll[arrayAnnotations[[All, tumorCenterColumn[[1]]]],
   {"HMS" → Red, "Both" → RGBColor[0.75, 0.75, 0.75], "MSKCC" → Blue}];

sFig2 = compositeFigure[pattern, condition, colors,
    rawTumorRaster, {}, {{68}, {0}}, {-.01, .01}, {-0.2, -0.1, 0, 0.1}, {}][[1]];

Export[path <> "Supplementary_Figure_2.pdf",
  sFig2, "PDF", ImageSize → 1000, ImageResolution → resolution];

(* Create 247th Probelet and Normal Arraylet Display *)

normalDateColumn = Flatten[Position[headers, "normal.scan.date"]];
colors = Table[RGBColor[.75, .75, .75], {251}];
bottom = Flatten[Position[arrayAnnotations[[All, normalDateColumn[[1]]]], "7.22.2009"]];
top = Flatten[Position[arrayAnnotations[[All, normalDateColumn[[1]]]], "10.8.2009"]];
colors[[bottom]] = Red;
colors[[top]] = Blue;
pattern = 247;
condition = "Normal";

sFig3 = compositeFigure[pattern, condition, colors, rasterContrast * rawNormalRaster,
    {}, {{38, 200}, {0}}, {-.01, .01}, {-0.2, -0.1, 0, 0.1}, {}][[1]];

Export[path <> "Supplementary_Figure_3.pdf",
  sFig3, "PDF", ImageSize → 1000, ImageResolution → resolution];

(* Create 248th Probelet and Normal Arraylet Display *)

normalBatchScannerColumn = Flatten[Position[headers, "normal.batch/scanner"]];
colors = Table[Blue, {251}];
batchScanner =
  Flatten[Position[arrayAnnotations[[All, normalBatchScannerColumn[[1]]]], "HMS_8/2331"]];
colors[[batchScanner]] = Red;
pattern = 248;
condition = "Normal";

sFig4 = compositeFigure[pattern, condition, colors,
    rasterContrast * rawNormalRaster, {}, {{232}, {0}}, {-.01, .01}, {-0.1, 0, 0.1}, {}][[1]];

Export[path <> "Supplementary_Figure_4.pdf",
  sFig4, "PDF", ImageSize → 1000, ImageResolution → resolution];

(* Create 249th Probelet and Normal Arraylet Display *)

normalBatchScannerColumn = Flatten[Position[headers, "normal.batch/scanner"]];
colors = Table[Blue, {251}];
batchScanner =
  Flatten[Position[arrayAnnotations[[All, normalBatchScannerColumn[[1]]]], "HMS_8/2331"]];
colors[[batchScanner]] = Red;
pattern = 249;
condition = "Normal";

sFig5 = compositeFigure[pattern, condition, colors, rasterContrast * rawNormalRaster, {},
    {{22}, {0}}, {-.01, .01}, {-0.1, 0, 0.1}, {}][[1]];

Export[path <> "Supplementary_Figure_5.pdf", sFig5, "PDF", ImageSize → 1000,
  ImageResolution → resolution];
```

```
(* Create 250th Probelet and Normal Arraylet Display *)

normalDateColumn = Flatten[Position[headers, "normal.scan.date"]];
colors = Table[RGBColor[.75, .75, .75], {251}];
bottom = Flatten[Position[arrayAnnotations[[All, normalDateColumn[[1]]]], "7.22.2009"]];
colors[[bottom]] = Blue;
top = Flatten[Position[arrayAnnotations[[All, normalDateColumn[[1]]]], "4.18.2007"]];
colors[[top]] = Red;
pattern = 250;
condition = "Normal";

sFig6 = compositeFigure[pattern, condition, colors, rasterContrast * rawNormalRaster, {},
     {{25, 225}, {0}}, {-.01, .01}, {-0.1, 0, 0.1}, {}][[1]];

Export[path <> "Supplementary_Figure_6.pdf", sFig6, "PDF", ImageSize → 1000,
   ImageResolution → resolution];

(* Create 251st Probelet and Normal Arraylet Display *)

normalCenterColumn = Flatten[Position[headers, "normal.center"]];
colors = ReplaceAll[arrayAnnotations[[All, normalCenterColumn[[1]]]],
    {"HMS" → Red, "Both" → RGBColor[.75, 0.75, 0.75], "MSKCC" → Blue}];
pattern = 251;
condition = "Normal";

sFig7 = compositeFigure[pattern, condition, colors, rasterContrast * rawNormalRaster, {},
     {{112}, {0}}, {-.01, .01}, {-0.1, 0, 0.05}, {}][[1]];

Export[path <> "Supplementary_Figure_7.pdf",
   sFig7, "PDF", ImageSize → 1000, ImageResolution → resolution];


 (* Create Tumor and Normal Generalized Fractions Bar Chart Displays *)

distances = Table[N[ArcTan[d1[[a, a]] / d2[[a, a]]] - Pi / 4], {a, 1, arrays}];
ylabels = {1, 50, 100, 150, 200, 250};

fractions1 = Table[d1^2[[a, a]], {a, 1, arrays}];
totalFractions1 = Total[fractions1];
fractions1 = fractions1 / totalFractions1;

fractions2 = Table[d2^2[[a, a]], {a, 1, arrays}];
totalFractions2 = Total[fractions2];
fractions2 = fractions2 / totalFractions2;

entropy1 = -N[Sum[fractions1[[a]] * Log[fractions1[[a]]], {a, 1, arrays}] / Log[arrays]];
entropy1 = Round[entropy1, 0.01];

arrayNum = 10;
ylabels = Range[arrayNum];
gridx = {0, 0.06, 0.12, 0.18, 0.24};
framex = {"0", "0.06", "0.12", "0.18", "0.24"};
framex = Table[{gridx[[a]], Style[Rotate[framex[[a]], Pi / 2]]}, {a, 1, 5}];
gridx = Table[{gridx[[a]], RGBColor[0, 0, 0]}, {a, 1, 5}];
framey =
   Table[{arrayNum - ylabels[[a]] + 1, "  " <> ToString[Reverse[Ordering[fractions1, -arrayNum]][[a]]]},
    {a, 1, Dimensions[ylabels][[1]]}];
labelx = ColumnForm[{"(a) Tumor Generalized Fraction", "d_1 = " <> ToString[entropy1]}, Center];
labely = "Probelets";
```

```
barsFractions1 = Show[BarChart[Reverse[Sort[fractions1, Greater][[1 ;; arrayNum]]], BarOrigin → Left,
     ChartStyle → Red, ChartBaseStyle → EdgeForm[Thin], AspectRatio → 1, Ticks → None,
     ImageSize → 250],
    PlotRange → {{0.0047, 0.24}, {0.5, arrayNum + 0.5}},
    Frame → True,
    FrameTicks → {None, framey, framex, None},
    FrameLabel → {None, labely, labelx, None},
    GridLines → {Drop[Drop[gridx, {5}], {1}], None},
    BaseStyle → {FontFamily → "Courier", FontSize → 10}
   ];

entropy2 = -N[Sum[fractions2[[a]] * Log[fractions2[[a]]], {a, 1, arrays}] / Log[arrays]];
entropy2 = Round[entropy2, 0.01];

arrayNum = 10;
ylabels = Range[arrayNum];
gridx = {0, 0.1, 0.2, 0.3, 0.4};
framex = {"0 ", "0.1 ", "0.2 ", "0.3 ", "0.4 "};
framex = Table[{gridx[[a]], Style[Rotate[framex[[a]], Pi / 2]]}, {a, 1, Length[framex]}];
gridx = Table[{gridx[[a]], RGBColor[0, 0, 0]}, {a, 1, Length[gridx]}];
framey = Table[{arrayNum - ylabels[[a]] + 1, Reverse[Ordering[fractions2, -arrayNum]][[a]]},
    {a, 1, Dimensions[ylabels][[1]]}];
labelx = ColumnForm[{"(b)  Normal Generalized Fraction", "d2 = " <> ToString[entropy2]}, Center];
labely = " ";

barsFractions2 = Show[BarChart[Reverse[Sort[fractions2, Greater][[1 ;; arrayNum]]], BarOrigin → Left,
     ChartStyle → Red, ChartBaseStyle → EdgeForm[Thin], AspectRatio → 1, Ticks → None,
     ImageSize → 250],
    PlotRange → {{0.008, 0.4}, {0.5, arrayNum + 0.5}},
    Frame -> True,
    FrameTicks → {None, framey, framex, None},
    FrameLabel → {None, labely, labelx, None},
    GridLines → {Drop[Drop[gridx, -1], 1], None},
    BaseStyle → {FontFamily → "Courier", FontSize → 10}
   ];

sFig1 = GraphicsGrid[{{barsFractions1, barsFractions2}}, ImageSize → 500, Spacings → -25]
```

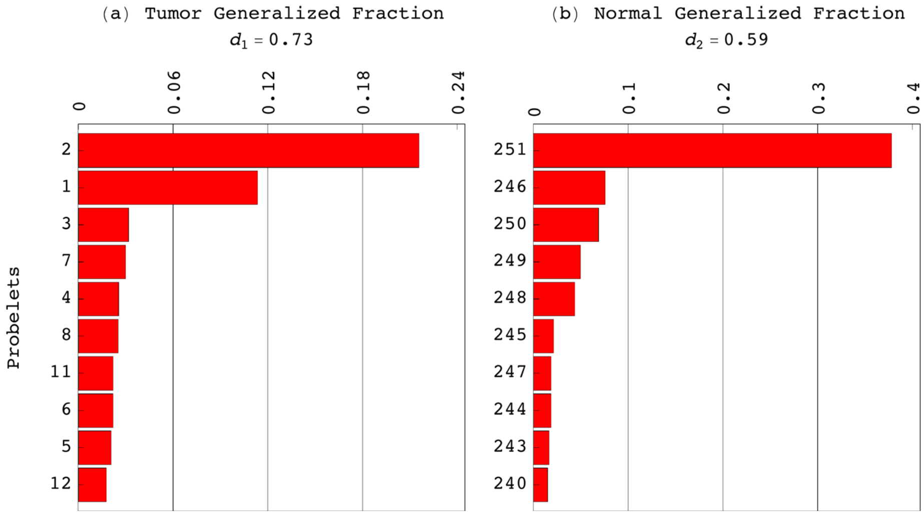


```
Export[path <> "Supplementary_Figure_1.pdf",
  sFig1, "PDF", ImageSize → 500, ImageResolution → resolution];
```

```
(* Create Angular Distances Bar Chart Display *)
```

```
arrayNum = 251;
ylabels = {1, 50, 100, 150, 200, 250};
gridx = {- π / 4, - π / 6, 0, 2 π / 9, π / 4};
framex = {"-π/4", "-π/6", "0", "2π/9", "π/4"};
framex = Table[{gridx[[a]], Style[Rotate[framex[[a]], Pi / 2]]}, {a, 1, 5}];
gridx = Table[{gridx[[a]], RGBColor[0, 0, 0]}, {a, 1, 5}];
framey = Table[{arrayNum - ylabels[[a]], ylabels[[a]]}, {a, 1, Dimensions[ylabels][[1]]}];
labelx = ColumnForm[{"Angular Distance"}, Center];
labely = "Probelets";

barsFull = Show[BarChart[Reverse[distances],
     BarOrigin → Left,
     ChartStyle → Red,
     ChartBaseStyle → EdgeForm[None],
     Axes → False,
     PlotRange → {{- π / 4 * 1.0001, π / 4 * 1.0001}, {0.5, arrayNum + 0.5}},
     AspectRatio → 1, ImageSize → 300],
   Frame -> True,
   FrameTicks → {None, framey, framex, None},
   FrameLabel → {None, labely, labelx, None},
   GridLines → {Drop[Drop[gridx, {5}], {1}], None},
   BaseStyle → {FontFamily → "Courier", FontSize → 10}];
```

```
(* Display GSVD of Patient-Matched Tumor and Normal aCGH Profiles *)
```

```
(* Create Tumor and Normal Arraylets Raster Displays *)

binnedArrayletTumor =
  Table[Mean[tumorArraylets[[a ;; a + step - 1]]], {a, 1, Floor[tumorProbes, step], step}];
{binnedRows, arrays} = Dimensions[binnedArrayletTumor];
raster = Table[adjustedValue = binnedArrayletTumor[[a, b]];
   If[adjustedValue > 0, {adjustedValue, 0, 0}, {0, adjustedValue, 0}],
   {a, 1, binnedRows}, {b, 1, arrays}];
raster[[All, All, 2]] = -raster[[All, All, 2]];
tumorArrayletsRaster = raster;

binnedArrayletNormal =
  Table[Mean[normalArraylets[[a ;; a + step - 1]]], {a, 1, Floor[normalProbes, step], step}];
{binnedRows, arrays} = Dimensions[binnedArrayletNormal];
raster = Table[adjustedValue = binnedArrayletNormal[[a, b]];
   If[adjustedValue > 0, {adjustedValue, 0, 0}, {0, adjustedValue, 0}],
   {a, 1, binnedRows}, {b, 1, arrays}];
raster[[All, All, 2]] = -raster[[All, All, 2]];
normalArrayletsRaster = raster;

(* Create Probelets Raster Display *)

arrayNum = 10;
numFractions = Dimensions[probelets][[1]];
fractList = Join[Range[1, arrayNum], Range[numFractions - arrayNum + 1, numFractions]];
contrast = 2;
fractions = Diagonal[d1] / Max[d1];
numFractionsDisplay = Dimensions[fractList][[1]];
d1Raster = Table[{0, 0, 0}, {a, 1, numFractionsDisplay}, {b, numFractionsDisplay}];
Do[d1Raster[[a, a, 1]] = contrast * fractions[[fractList[[a]]]], {a, 1, numFractionsDisplay}];
fractions = Diagonal[d2] / Max[d2];
numFractionsDisplay = Dimensions[fractList][[1]];
d2Raster = Table[{0, 0, 0}, {a, 1, numFractionsDisplay}, {b, numFractionsDisplay}];
Do[d2Raster[[a, a, 1]] = contrast * fractions[[fractList[[a]]]], {a, 1, numFractionsDisplay}];
probeletsRaster = Table[adjustedValue = probelets[[a, b]];
   If[adjustedValue > 0, {adjustedValue, 0, 0}, {0, adjustedValue, 0}],
   {a, 1, Dimensions[probelets][[1]]}, {b, 1, Dimensions[probelets][[2]]}];
probeletsRaster[[All, All, 2]] = -probeletsRaster[[All, All, 2]];
```

```
(* Define GSVD Display *)

genes1 = 680;
genes2 = 840;
yOffset = 126;
totalArrays = 251;
arrays = yOffset;
space = 0;
xOffset = 0;
labelMargin = 0.1;
labelMarginVertical = 0.45;
labelMarginHorizontal = 0.3;
labelSize = 10;
ticksLabelSize = 8;
chromosomes = Join[Range[1, 22], {"X"}];
arrayNum = 10;
numChromosomes = 23;
ticks = {1, 50, 100, 150, 200, 250};
ticksShort = {{1, 0.5 / (2 * arrayNum)},
   {5, 4.5 / (2 * arrayNum)}, {246, (245.5 - totalArrays + 2 * arrayNum) / (2 * arrayNum)},
   {250, (249.5 - totalArrays + 2 * arrayNum) / (2 * arrayNum)}};

(* Define Tumor and Normal Datasets Display *)

dataTensor = {
   Graphics[{Red, Raster[Reverse[2 * rawNormalRaster[[All, Ordering[probelets[[2]]]]]],
      {{0 + xOffset + space, genes2 / 2 - genes1}, {arrays + xOffset, genes2 / 2}}]}],
   Graphics[{Blue, Raster[Reverse[2 * rawTumorRaster[[All, Ordering[probelets[[2]]]]]],
      {{arrays + xOffset + space, -genes2 / 2 + yOffset},
       {2 * arrays + xOffset + space, genes2 / 2 + yOffset}}]}],
   Graphics[Text[StyleForm["\!\(\*TagBox[SubscriptBox[\"D\", \"normal\"],DisplayForm]\)",
      FontSize → labelSize], {-arrays * .25, genes2 * .5 + arrays * .25}, {0, -1}]],
   Graphics[Text[StyleForm["\!\(\*TagBox[SubscriptBox[\"D\", \"tumor\"],DisplayForm]\)",
      FontSize → labelSize], {arrays * .75, genes2 * .5 + arrays * .25 + yOffset}, {0, -1}]],
   Graphics[Line[{{-arrays * .15, genes2 * .5 + arrays * .15}, {0, genes2 / 2}}]],
   Graphics[Line[{{arrays * .85, genes2 * .5 + arrays * .15 + yOffset}, {arrays, genes2 / 2 + yOffset}}]],
   Graphics[Line[{{0, genes2 / 2}, {arrays, genes2 / 2 + yOffset}}]],
   Graphics[{White, Dashed, Line[{{arrays, genes2 / 2}, {2 * arrays, genes2 / 2 + yOffset}}]}],
   Graphics[
    Style[Text["Tissue Types", {arrays * 0 - 10, genes2 * .5 + arrays}, {0, 0}, {1, 1}], labelSize]],
   Table[Graphics[{Text[Style[ticks[[a]], ticksLabelSize],
       {xOffset + arrays + arrays * ticks[[a]] / 251,
        genes2 / 2 + yOffset + arrays * labelMargin}, {-1, 0}, {0, 1}],
      Line[{{xOffset + arrays + arrays * ticks[[a]] / 251, genes2 / 2 + yOffset},
        {xOffset + arrays + arrays * ticks[[a]] / 251, genes2 / 2 + yOffset + 8}}]
     }], {a, Length[ticks]}],
   Table[Graphics[{Text[Style[chromosomes[[a]], ticksLabelSize],
       {xOffset - arrays * labelMargin,
        genes2 / 2 - normalChromosomeBounds[[a, 1]] * (genes1 / normalProbes)}, {1, 0}, {1, 0}],
      Line[{{xOffset - 8, genes2 / 2 - normalChromosomeBounds[[a, 1]] * (genes1 / normalProbes)},
        {xOffset, genes2 / 2 - normalChromosomeBounds[[a, 1]] * (genes1 / normalProbes)}}],
      Text[Style[chromosomes[[a]], ticksLabelSize], {xOffset + arrays * (2 + labelMargin),
        genes2 / 2 + yOffset - tumorChromosomeBounds[[a, 1]] * (genes2 / tumorProbes)}, {-1, 0}, {1, 0}],
      Line[{{xOffset + 2 * arrays, genes2 / 2 + yOffset - tumorChromosomeBounds[[a, 1]] *
           (genes2 / tumorProbes)},
        {xOffset + 2 * arrays + 8, genes2 / 2 + yOffset -
          tumorChromosomeBounds[[a, 1]] * (genes2 / tumorProbes)}}]}], {a, 1, numChromosomes}],
   Graphics[Text["Arrays", {arrays * 1.5, genes2 * .5 + yOffset + arrays * labelMarginVertical}]],
   Graphics[Text["Normal Probes ",
     {-arrays * labelMarginHorizontal, genes2 / 2 - arrays * 0.7}, {0, 0}, {0, 1}]],
   Graphics[Text["Tumor Probes", {2 * arrays + xOffset + arrays * labelMarginHorizontal,
      genes2 / 2 + yOffset - arrays * 0.7}, {0, 0}, {0, 1}]],
   Graphics[{White, Thin, Line[{{arrays, -genes2 / 2 + yOffset}, {arrays, genes2 / 2 + yOffset}}]}]};
```

```
Dimensions[rawNormalRaster];
normalArrayletsRasterShort = Drop[normalArrayletsRaster, None, {arrayNum + 1, 251 - arrayNum}];
Dimensions[normalArrayletsRasterShort];
tumorArrayletsRasterShort = Drop[tumorArrayletsRaster, None, {arrayNum + 1, 251 - arrayNum}];
Dimensions[tumorArrayletsRasterShort];

(* Define Tumor and Normal Arraylets Display *)

xOffset = 3.5 * arrays;
arrayletTensor = {
   Graphics[{Red, Raster[Reverse[200 * normalArrayletsRasterShort, {1}],
      {{xOffset, genes2 / 2 - genes1}, {arrays + xOffset, genes2 / 2}}]}],
   Graphics[{Blue, Raster[Reverse[200 * tumorArrayletsRasterShort, {1}], {{arrays + xOffset + space,
        -genes2 / 2 + yOffset}, {2 * arrays + xOffset + space, genes2 / 2 + yOffset}}]}],
   Graphics[Text[StyleForm["\!\(\*TagBox[SubscriptBox[\"U\", \"normal\"],DisplayForm]\)",
      FontSize → labelSize], {-arrays * .25 + xOffset, genes2 * .5 + arrays * .25}, {0, -1}]],
   Graphics[Text[StyleForm["\!\(\*TagBox[SubscriptBox[\"U\", \"tumor\"],DisplayForm]\)",
      FontSize → labelSize], {arrays * .75 + xOffset, genes2 * .5 + arrays * .25 + yOffset}, {0, -1}]],
   Graphics[Line[{{-arrays * .15 + xOffset, genes2 * .5 + arrays * .15}, {0 + xOffset, genes2 / 2}}]],
   Graphics[Line[{{arrays * .85 + xOffset, genes2 * .5 + arrays * .15 + yOffset},
      {arrays + xOffset, genes2 / 2 + yOffset}}]],
   Graphics[Line[{{0 + xOffset, genes2 / 2}, {arrays + xOffset, genes2 / 2 + yOffset}}]],
   Graphics[{White, Dashed, Line[{{arrays + xOffset, genes2 / 2},
       {2 * arrays + xOffset, genes2 / 2 + yOffset}}]}],
   Graphics[Style[
     Text["Tissue Types", {arrays * 0 + xOffset - 10, genes2 * .5 + arrays}, {0, 0}, {1, 1}], labelSize]],
   Table[Graphics[{Text[Style[ticksShort[[a, 1]], ticksLabelSize],
       {xOffset + arrays + arrays * ticksShort[[a, 2]],
        genes2 / 2 + yOffset + arrays * labelMargin}, {-1, 0}, {0, 1}],
      Line[{{xOffset + arrays + arrays * ticksShort[[a, 2]], genes2 / 2 + yOffset},
        {xOffset + arrays + arrays * ticksShort[[a, 2]], genes2 / 2 + yOffset + 8}}]
     }], {a, Length[ticksShort]}],
   Graphics[{White, Line[{{arrays / 2 + xOffset, genes2 / 2 - genes1},
       {arrays / 2 + xOffset, genes2 / 2}}]}],
   Graphics[{White, Line[{{arrays * 1.5 + xOffset, -genes2 / 2 + yOffset},
       {arrays * 1.5 + xOffset, genes2 / 2 + yOffset}}]}],
   Table[Graphics[{Text[Style[chromosomes[[a]], ticksLabelSize],
       {xOffset - arrays * labelMargin,
        genes2 / 2 - normalChromosomeBounds[[a, 1]] * (genes1 / normalProbes)}, {1, 0}, {1, 0}],
      Line[{{xOffset - 8, genes2 / 2 - normalChromosomeBounds[[a, 1]] * (genes1 / normalProbes)},
        {xOffset, genes2 / 2 - normalChromosomeBounds[[a, 1]] * (genes1 / normalProbes)}}],
      Text[Style[chromosomes[[a]], ticksLabelSize], {xOffset + arrays * (2 + labelMargin),
        genes2 / 2 + yOffset - tumorChromosomeBounds[[a, 1]] * (genes2 / tumorProbes)}, {-1, 0}, {1, 0}],
      Line[{{xOffset + 2 * arrays, genes2 / 2 + yOffset - tumorChromosomeBounds[[a, 1]] *
           (genes2 / tumorProbes)},
        {xOffset + 2 * arrays + 8, genes2 / 2 + yOffset - tumorChromosomeBounds[[a, 1]] *
           (genes2 / tumorProbes)}}]}], {a, 1, numChromosomes}],
   Graphics[Text["Arraylets",
     {arrays * 1.5 + xOffset, genes2 * .5 + yOffset + arrays * labelMarginVertical}]],
   Graphics[Text["Normal Probes  ",
     {-arrays * labelMarginHorizontal + xOffset, (genes2 / 2) - arrays * 0.7}, {0, 0}, {0, 1}]],
   Graphics[Text["Tumor Probes", {2 * arrays + xOffset + arrays * labelMarginHorizontal,
      genes2 / 2 + yOffset - arrays * 0.7}, {0, 0}, {0, 1}]],
   Graphics[{White, Thin, Line[{{arrays + xOffset, -genes2 / 2 + yOffset},
       {arrays + xOffset, genes2 / 2 + yOffset}}]}]};
```

```
(* Define Tumor and Normal Generalized Singular Values Display *)

xOffset = 6.6 * arrays;
coreTensor = {
   Graphics[{Raster[Reverse[d1Raster], {{arrays + xOffset + space, genes2 / 2 + yOffset - arrays},
        {2 * arrays + xOffset + space, genes2 / 2 + yOffset}}]}],
   Graphics[{Raster[Reverse[d2Raster], {{0 + xOffset, genes2 / 2 - arrays},
        {arrays + xOffset, genes2 / 2}}]}],
   Graphics[Text[StyleForm["\!\(\*TagBox[SubscriptBox[\"Σ\", \"normal\"],DisplayForm]\)",
      FontSize → labelSize], {-arrays * .25 + xOffset, genes2 * .5 + arrays * .25}, {0, -1}]],
   Graphics[Text[StyleForm["\!\(\*TagBox[SubscriptBox[\"Σ\", \"tumor\"],DisplayForm]\)",
      FontSize → labelSize], {arrays * .75 + xOffset, genes2 * .5 + arrays * .25 + yOffset}, {0, -1}]],
   Graphics[Line[{{-arrays * .15 + xOffset, genes2 * .5 + arrays * .15}, {0 + xOffset, genes2 / 2}}]],
   Graphics[Line[{{arrays * .85 + xOffset, genes2 * .5 + arrays * .15 + yOffset},
      {arrays + xOffset, genes2 / 2 + yOffset}}]],
   Graphics[Line[{{0 + xOffset, genes2 / 2}, {arrays + xOffset, genes2 / 2 + yOffset}}]],
   Graphics[{White, Dashed, Line[{{xOffset + arrays, genes2 / 2},
        {2 * arrays + xOffset, genes2 / 2 + yOffset}}]}],
   Graphics[Line[{{arrays + xOffset, genes2 / 2 - arrays}, {2 * arrays + xOffset, genes2 / 2}}]],
   Graphics[Style[
     Text["Tissue Types", {arrays * 0 + xOffset - 10, genes2 * .5 + arrays}, {0, 0}, {1, 1}], labelSize]],
   Table[Graphics[{
      Text[Style[ticksShort[[a, 1]], ticksLabelSize], {xOffset + arrays + arrays * ticksShort[[a, 2]],
        genes2 / 2 + yOffset + arrays * labelMargin}, {-1, 0}, {0, 1}],
      Line[{{xOffset + arrays + arrays * ticksShort[[a, 2]], genes2 / 2 + yOffset},
        {xOffset + arrays + arrays * ticksShort[[a, 2]], genes2 / 2 + yOffset + 8}}]
     }], {a, Length[ticksShort]}],
   Table[Graphics[{
      Text[Style[ticksShort[[a, 1]], ticksLabelSize], {arrays * -labelMargin + xOffset,
        genes2 / 2 + yOffset - arrays - arrays * ticksShort[[a, 2]]}, {1, 0}, {1, 0}],
      Line[{{xOffset - 8, genes2 / 2 + yOffset - arrays - arrays * ticksShort[[a, 2]]},
        {xOffset, genes2 / 2 + yOffset - arrays - arrays * ticksShort[[a, 2]]}}]
     }], {a, Length[ticksShort]}],
   Graphics[{White, Line[{{arrays + xOffset, genes2 / 2 - arrays / 2 + yOffset},
        {arrays * 2 + xOffset, genes2 / 2 - arrays / 2 + yOffset}}]}],
   Graphics[{White, Line[{{arrays * 1.5 + xOffset, genes2 / 2 - arrays + yOffset},
        {arrays * 1.5 + xOffset, genes2 / 2 + yOffset}}]}],
   Graphics[{White, Line[{{xOffset, genes2 / 2 - arrays / 2},
        {arrays + xOffset, genes2 / 2 - arrays / 2}}]}],
   Graphics[{White, Line[{{arrays / 2 + xOffset, genes2 / 2 - arrays},
        {arrays / 2 + xOffset, genes2 / 2}}]}],
   Graphics[Text["Probelets",
     {arrays * 1.5 + xOffset, genes2 * .5 + yOffset + arrays * labelMarginVertical}]],
   Graphics[Text["Arraylets", {-arrays * 0.45 + xOffset, genes2 * .5 - arrays * .5}, {0, 0}, {0, 1}]]
  };
```

```
(* Define Probelets Display *)

probeletsRasterShort =
  Drop[probeletsRaster[[All, Ordering[probelets[[2]]]]], {arrayNum + 1, 251 - arrayNum}];
xOffset = 8.3 * arrays;
probeletsDispley = {
   Graphics[{Raster[10 * Reverse[probeletsRasterShort],
       {{arrays + xOffset, genes2 / 2 - arrays + yOffset}, {2 * arrays + xOffset, genes2 / 2 + yOffset}}]}],
   Table[Graphics[{
      Text[Style[ticks[[a]], ticksLabelSize], {xOffset + arrays + arrays * ticks[[a]] / 251,
        genes2 / 2 + yOffset + arrays * labelMargin}, {-1, 0}, {0, 1}],
      Line[{{xOffset + arrays + arrays * ticks[[a]] / 251, genes2 / 2 + yOffset},
        {xOffset + arrays + arrays * ticks[[a]] / 251, genes2 / 2 + yOffset + 8}}]
     }], {a, Length[ticks]}],
   Table[Graphics[{Text[Style[ticksShort[[a, 1]], ticksLabelSize],
       {arrays * (1 - labelMargin) + xOffset,
        genes2 / 2 + yOffset - arrays * ticksShort[[a, 2]]}, {1, 0}, {1, 0}],
      Line[{{arrays + xOffset - 8, genes2 / 2 + yOffset - arrays * ticksShort[[a, 2]]},
        {arrays + xOffset, genes2 / 2 + yOffset - arrays * ticksShort[[a, 2]]}}]
     }], {a, Length[ticksShort]}],
   Graphics[{White, Line[{{arrays + xOffset, genes2 / 2 - arrays / 2 + yOffset},
       {arrays * 2 + xOffset, genes2 / 2 - arrays / 2 + yOffset}}]}],
   Graphics[Text["Arrays",
     {arrays * 1.5 + xOffset, genes2 * .5 + yOffset + arrays * labelMarginVertical}]],
   Graphics[Text["Probelets",
     {arrays * 0.55 + xOffset, (genes2 / 2) - arrays * .5 + yOffset}, {0, 0}, {0, 1}]],
   Graphics[Text[Style["V^T", labelSize],
     {arrays * .75 + xOffset, genes2 * .5 + arrays * .25 + yOffset}, {0, 0}]],
   Graphics[Line[{{arrays * .85 + xOffset, genes2 * .5 + arrays * .15 + yOffset},
      {arrays + xOffset, genes2 / 2 + yOffset}}]]
  };

equal = Graphics[Text[StyleForm["≃", FontSize → 60], {arrays * 2.65, genes2 / 2 - arrays * .5}, {0, 0}]];

barsInset = Graphics[{Inset[barsFull, {6.15 * arrays, genes2 / 2 - 4 * arrays},
     {-1.02, 113}, {4.25 * arrays, Automatic}]}];
```

```
fig1 = Show[dataTensor, arrayletTensor, coreTensor, probeletsDispley,
  equal, barsInset, ImageSize → 750, PlotRange → {{-70, 1320}, {-310, 620}},
  BaseStyle → {FontFamily → "Courier", FontSize → labelSize}]
```

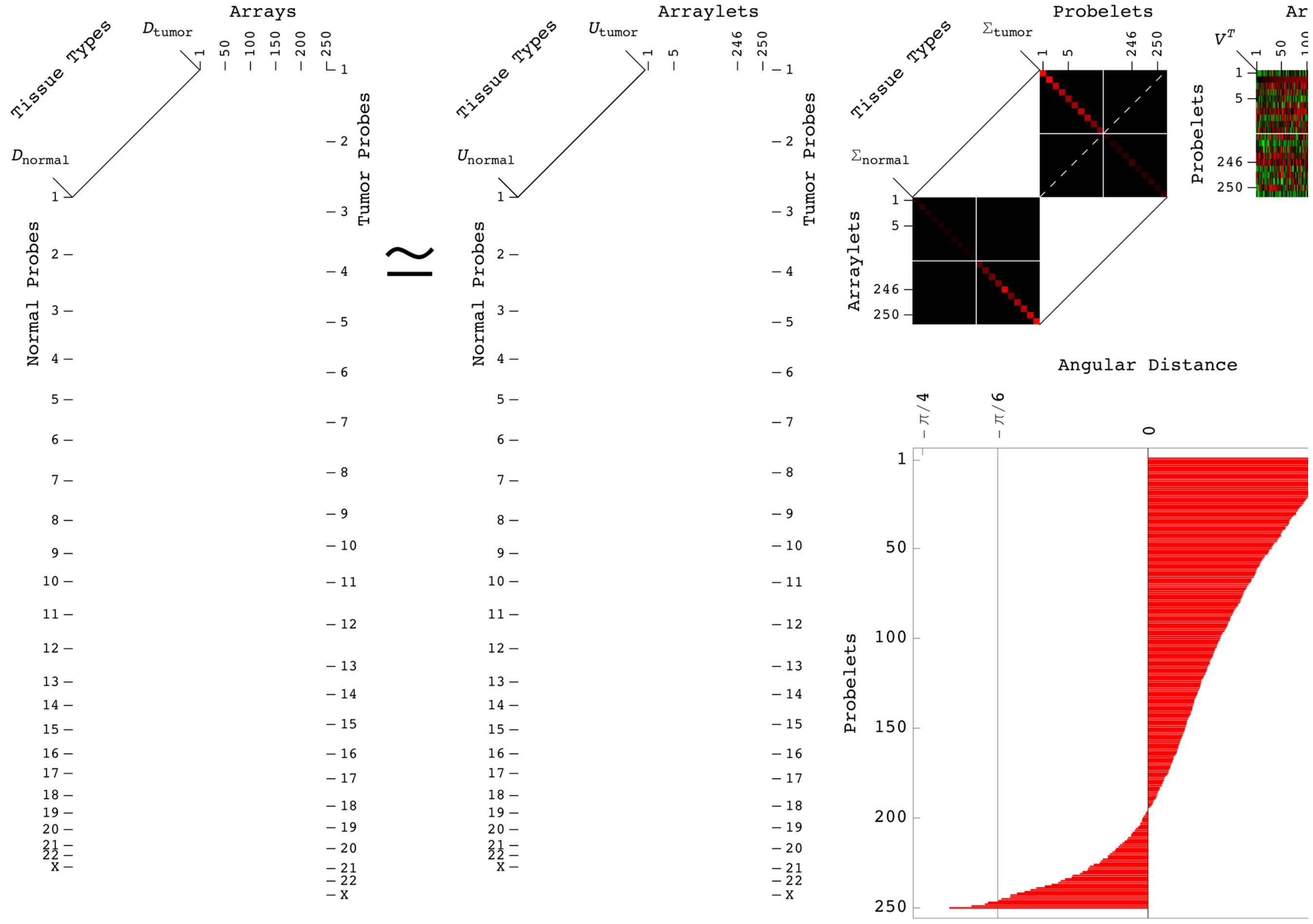


```
Export[path <> "Figure_1.pdf", fig1, "PDF", ImageSize → 750, ImageResolution → resolution];
```

```
(* Display Distributions of Copy Numbers Among Patient Annotations *)
```

```
(* Define Boxplot Display *)

boxPlot[data_, dataLabels_, frameLabels_, frameTicks_, plotLabel_, plotRange_] := {
  nData = Dimensions[data][[1]];
  colors = {Red, Blue, Black};
  pValue = MannWhitneyTest[{data[[1]], data[[2]]}];
  g = Table[{}, {a, 1, nData}];
  bins = Flatten[
    Table[Labeled[{}, Style[ColumnForm[{
         dataLabels[[a]], "N=" <> ToString[Length[data[[a]]]]},
        Center], colors[[a]]]], {a, 2, nData}]];
  Do[g[[a]] = BoxWhiskerChart[
      Flatten[
       If[a == 1, {
         Labeled[data[[a]], Style[ColumnForm[{
             dataLabels[[a]], "N=" <> ToString[Length[data[[a]]]]}, Center], colors[[a]]]],
         If[nData == 3, {bins[[1]], bins[[2]]}, bins[[1]]]}, {
         Table[If[b == a,
           Labeled[data[[b]], ColumnForm[{
              dataLabels[[b]], "N=" <> ToString[Length[data[[b]]]]}, Center]],
           {}], {b, 1, nData}]}], 1],
      {"Median", {"Outliers", "●", colors[[a]]},
       {"MedianMarker", colors[[a]]}, {"Whiskers", colors[[a]]}, {"Fences", colors[[a]]}},
      PlotLabel → ColumnForm[{plotLabel[[1]],
         StringJoin["P-value = ", ToString[TraditionalForm[ScientificForm[pValue, 2,
             NumberPoint →
              If[Dimensions[Characters[ToString[NumberForm[pValue, 2, NumberFormat → (#1 &),
                     ExponentFunction → (# &)]]]][[1]] < 3, "", "."]]
           ]]]}, Center],
      If[Length[plotRange] > 0,
       PlotRange → plotRange, PlotRange → {{0.48, Automatic}, {-0.22, 0.22}}],
      FrameTicks → {{frameTicks, None}, {None, None}},
      FrameLabel → Table[frameLabels[[a]], {a, Length[frameLabels]}],
      ImageSize → 250,
      BarSpacing → Tiny,
      ChartBaseStyle → Directive[EdgeForm[{Thick, colors[[a]]}], White, Thick],
      AspectRatio → 1,
      BaseStyle → {FontFamily → "Courier", FontSize → 10}
     ], {a, 1, nData}];
  Show[Table[g[[a]], {a, 1, nData}]]
 }

(* Create First Probelet Boxplot Display *)

tumorCenterColumn = Flatten[Position[headers, "tumor.center"]];
probelet1Column = Flatten[Position[headers, "probelet.1"]];

bothPos = Flatten[Position[arrayAnnotations[[All, tumorCenterColumn[[1]]]], "Both"]];
hmsPos = Flatten[Position[arrayAnnotations[[All, tumorCenterColumn[[1]]]], "HMS"]];
mskccPos = Flatten[Position[arrayAnnotations[[All, tumorCenterColumn[[1]]]], "MSKCC"]];
data = {Null, Null, Null};
both = arrayAnnotations[[bothPos, probelet1Column[[1]]]];
hms = arrayAnnotations[[hmsPos, probelet1Column[[1]]]];
mskcc = arrayAnnotations[[mskccPos, probelet1Column[[1]]]];

g1 = boxPlot[
    {hms, mskcc, both},
    {"HMS", "MSKCC", "Both"},
    {"Tumor Sample Center", "Relative DNA Copy Number"},
    {{-0.2, "-0.2"}, {-0.1, "-0.1"}, 0, {0.1, "0.1"}, {0.2, "0.2"}},
    {"(a) Probelet 1"}, {}][[1]];
```

```
(* Create 247th Probelet Boxplot Display *)

normalDateColumn = Flatten[Position[headers, "normal.scan.date"]];
probelet247Column = Flatten[Position[headers, "probelet.247"]];

bottomPos = Flatten[Position[arrayAnnotations[[All, normalDateColumn[[1]]]], "7.22.2009"]];
topPos = Flatten[Position[arrayAnnotations[[All, normalDateColumn[[1]]]], "10.8.2009"]];
otherPos = Delete[Table[a, {a, rows}], Partition[Join[topPos, bottomPos], 1]];
bottom = arrayAnnotations[[bottomPos, probelet247Column[[1]]]];
top = arrayAnnotations[[topPos, probelet247Column[[1]]]];
other = arrayAnnotations[[otherPos, probelet247Column[[1]]]];
pValue = MannWhitneyTest[{bottom, top}];

g2 = boxPlot[
     {bottom, top, other},
     {"7.22.2009", "10.8.2009", "Other"},
     {"Normal Sample Scan Date", ""}, {{-0.2, "    "}, {-0.1, "    "},
      {0, "    "}, {0.1, "    "}, {0.2, "    "}}, {"(b) Probelet 247"}, {}][[1]];

(* Create 248th Probelet Boxplot Display *)

normalBatchScannerColumn = Flatten[Position[headers, "normal.batch/scanner"]];
probelet248Column = Flatten[Position[headers, "probelet.248"]];

hmsPos = Flatten[Position[arrayAnnotations[[All, normalBatchScannerColumn[[1]]]], "HMS_8/2331"]];
hms = arrayAnnotations[[hmsPos, probelet248Column[[1]]]];
otherPos = Complement[Table[a, {a, rows}], hmsPos];
other = arrayAnnotations[[otherPos, probelet248Column[[1]]]];

g3 = boxPlot[
     {hms, other},
     {"HMS 8/2331", "Other"},
     {"Normal Sample Batch/Scanner", ""}, {{-0.2, "    "}, {-0.1, "    "},
      {0, "    "}, {0.1, "    "}, {0.2, "    "}}, {"(c) Probelet 248"}, {}][[1]];

(* Create 249th Probelet Boxplot Display *)

normalBatchScannerColumn = Flatten[Position[headers, "normal.batch/scanner"]];
probelet249Column = Flatten[Position[headers, "probelet.249"]];

hmsPos = Flatten[Position[arrayAnnotations[[All, normalBatchScannerColumn[[1]]]], "HMS_8/2331"]];
hms = arrayAnnotations[[hmsPos, probelet249Column[[1]]]];
otherPos = Complement[Table[a, {a, rows}], hmsPos];
other = arrayAnnotations[[otherPos, probelet249Column[[1]]]];

g4 = boxPlot[
     {hms, other},
     {"HMS 8/2331", "Other"},
     {"Normal Sample Batch/Scanner", "Relative DNA Copy Number"},
     {{-0.2, "-0.2"}, {-0.1, "-0.1"}, 0, {0.1, "0.1"}, {0.2, "0.2"}},
     {"(d) Probelet 249"}, {}][[1]];

(* Create 250th Probelet Boxplot Display *)

normalDateColumn = Flatten[Position[headers, "normal.scan.date"]];
probelet250Column = Flatten[Position[headers, "probelet.250"]];

augPos = Flatten[Position[arrayAnnotations[[All, normalDateColumn[[1]]]], "4.18.2007"]];
julyPos = Flatten[Position[arrayAnnotations[[All, normalDateColumn[[1]]]], "7.22.2009"]];
otherPos = Delete[Table[a, {a, 251}], Partition[Join[augPos, julyPos], 1]];
aug = arrayAnnotations[[augPos, probelet250Column[[1]]]];
july = arrayAnnotations[[julyPos, probelet250Column[[1]]]];
other = arrayAnnotations[[otherPos, probelet250Column[[1]]]];

g5 = boxPlot[
     {aug, july, other},
     {"4.18.2007", "7.22.2009", "Other"},
     {"Normal Sample Scan Date", ""}, {{-0.2, "    "}, {-0.1, "    "},
      {0, "    "}, {0.1, "    "}, {0.2, "    "}}, {"(e) Probelet 250"}, {}][[1]];
```

```
(* Create 251st Probelet Boxplot Display *)

normalCenterColumn = Flatten[Position[headers, "normal.center"]];
probelet251Column = Flatten[Position[headers, "probelet.251"]];

hmsPos = Flatten[Position[arrayAnnotations[[All, normalCenterColumn[[1]]]], "HMS"]];
mskccPos = Flatten[Position[arrayAnnotations[[All, normalCenterColumn[[1]]]], "MSKCC"]];
data = {Null, Null, Null};
bothPos = Flatten[Position[arrayAnnotations[[All, normalCenterColumn[[1]]]], "Both"]];
hms = arrayAnnotations[[hmsPos, probelet251Column[[1]]]];
mskcc = arrayAnnotations[[mskccPos, probelet251Column[[1]]]];
both = arrayAnnotations[[bothPos, probelet251Column[[1]]]];

g6 = boxPlot[
    {hms, mskcc, both},
    {"HMS", "MSKCC", "Both"},
    {"Normal Sample Center", ""},
    {{-0.2, "    "}, {-0.1, "     "}, {0, "     "}, {0.1, "    "}, {0.2, "    "}},
    {"(f) Probelet 251"}, {}][[1]];

sFig8 = GraphicsGrid[{{g1, g2, g3}, {g4, g5, g6}}, Spacings → {-55, 15}, ImageSize → 750]
```

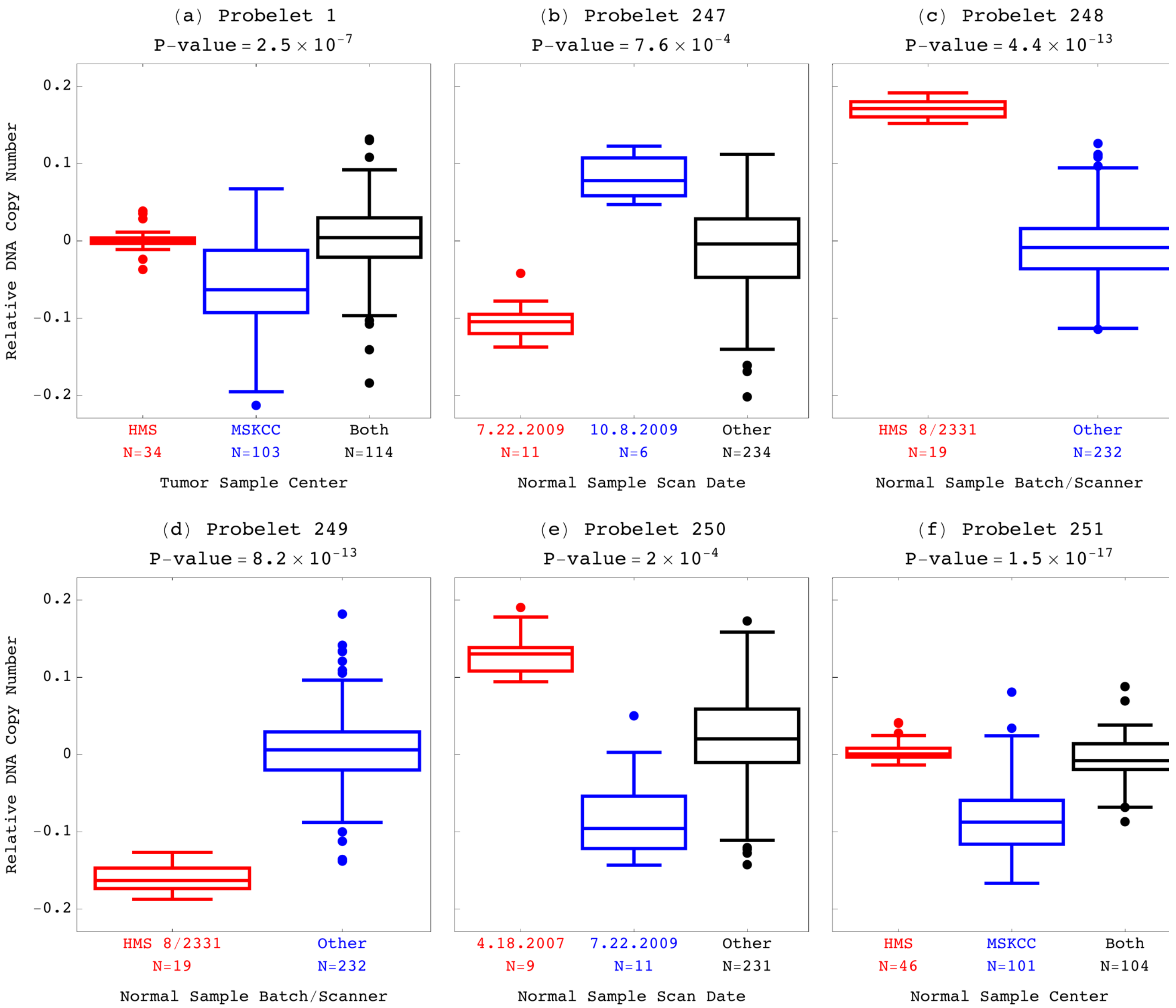


```
Export[path <> "Supplementary_Figure_8.pdf",
  sFig8, "PDF", ImageSize → 750, ImageResolution → resolution];
```

```
(* Redefine Boxplot Display *)

boxPlot[data_, dataLabels_, frameLabels_,
  frameTicks_, plotLabel_, plotRange_, pValueLabel_, outlierStyle_] := {
  nData = Dimensions[data][[1]];
  colors = {Red, Blue, Black};
  pValue = MannWhitneyTest[{data[[1]], data[[2]]}];
  g = Table[{}, {a, 1, nData}];
  bins = Flatten[
    Table[Labeled[{}, Style[ColumnForm[{
          dataLabels[[a]], "N=" <> ToString[NumberForm[Length[data[[a]]], DigitBlock → 3]]},
         Center], colors[[a]]]], {a, 2, nData}]];
  Do[g[[a]] = BoxWhiskerChart[
     Flatten[
      If[a == 1, {
         Labeled[data[[a]], Style[ColumnForm[{
             dataLabels[[a]], "N=" <> ToString[NumberForm[Length[data[[a]]], DigitBlock → 3]]},
            Center], colors[[a]]]],
         If[nData == 3, {bins[[1]], bins[[2]]}, bins[[1]]]}, {
         Table[If[b == a, Labeled[data[[b]], ColumnForm[{
              dataLabels[[b]], "N=" <> ToString[Length[data[[b]]]]}, Center]],
           {}], {b, 1, nData}]}], 1],
     {"Median", {"Outliers", outlierStyle, colors[[a]]},
      {"MedianMarker", colors[[a]]}, {"Whiskers", colors[[a]]}, {"Fences", colors[[a]]}},
     If[Length[pValueLabel] ≠ 0,
      PlotLabel → ColumnForm[{plotLabel[[1]],
         pValueLabel[[1]]}, Center],
      PlotLabel → ColumnForm[{plotLabel[[1]],
         StringJoin["P-value = ", ToString[TraditionalForm[ScientificForm[pValue, 2,
             NumberPoint →
              If[Dimensions[Characters[ToString[NumberForm[pValue, 2, NumberFormat → (#1 &),
                     ExponentFunction → (# &)]]]][[1]] < 3, "", "."]]
           ]]]]}, Center]],
     If[Length[plotRange] > 0,
      PlotRange → plotRange, PlotRange → {{0.48, Automatic}, {-0.22, 0.22}}],
     FrameTicks → {{frameTicks, None}, {None, None}},
     FrameLabel → Table[frameLabels[[a]], {a, Length[frameLabels]}],
     ImageSize → 250,
     BarSpacing → Tiny,
     ChartBaseStyle → Directive[EdgeForm[{Thick, colors[[a]]}], White, Thick],
     AspectRatio → 1,
     BaseStyle → {FontFamily → "Courier", FontSize → 10}
    ], {a, 1, nData}];
  Show[Table[g[[a]], {a, 1, nData}]]
 }
```

```
(* Create 246th Probelet and Corresponding Tumor and Normal Arraylets Boxplot Displays *)

xTumorLoc = tumorChromosomeBounds[[23, 1]];
xNormalLoc = normalChromosomeBounds[[23, 1]];
tumorArraylet246 = tumorArraylets[[All, 246]];
normalArraylet246 = normalArraylets[[All, 246]];

genderColumn = Flatten[Position[headers, "copy-number.gender"]];
probelet246Column = Flatten[Position[headers, "probelet.246"]];

male = arrayAnnotations[[
   Flatten[Position[arrayAnnotations[[All, genderColumn[[1]]]], "MALE"]], probelet246Column[[1]]]];
female = arrayAnnotations[[Flatten[Position[arrayAnnotations[[All, genderColumn[[1]]]], "FEMALE"]],
   probelet246Column[[1]]]];

probelet246 = boxPlot[
     {female, male},
     {"Female", "Male"},
     {"Patient Gender", "Relative DNA Copy Number"},
     {{-0.05, "-0.05"}, 0, {0.05, "0.05"}, {0.1, "    0.1"}, {0.15, "0.15"}},
     {"(a) Probelet 246"}, {{0.48, Automatic}, {-0.05, 0.15}}, {}, "●"][[1]];

normal246 = boxPlot[{normalArraylet246[[xNormalLoc + 1 ;; Length[normalArraylet246]]],
      normalArraylet246[[1 ;; xNormalLoc]]},
     {"X Chromosome", "Autosomal"},
     {"Normal Probe Location", " "},
     {{-0.01, "-0.01"}, {-0.005, "-0.005"}, 0, {0.005, "0.005"},
      {0.01, "0.01"}, {0.015, "0.015"}}, {"(b) Normal Arraylet 246"},
     {{0.48, Automatic}, {-0.005, 0.015}},
     {"P-value≪10^-38"}, ""][[1]];

tumor246 = boxPlot[{tumorArraylet246[[xTumorLoc + 1 ;; Length[tumorArraylet246]]],
      tumorArraylet246[[1 ;; xTumorLoc]]},
     {"X Chromosome", "Autosomal"},
     {"Tumor Probe Location", " "},
     {{-0.01, "-0.01"}, {-0.005, "-0.005"}, 0, {0.005, "0.005"}, {0.01, "0.01"}},
     {"(c) Tumor Arraylet 246"},
     {{0.48, Automatic}, {-0.01, 0.01}},
     {"P-value≪10^-38"}, ""][[1]];

sFig9 = GraphicsGrid[{{probelet246, normal246, tumor246}}, Spacings → -30, ImageSize → 750]
```

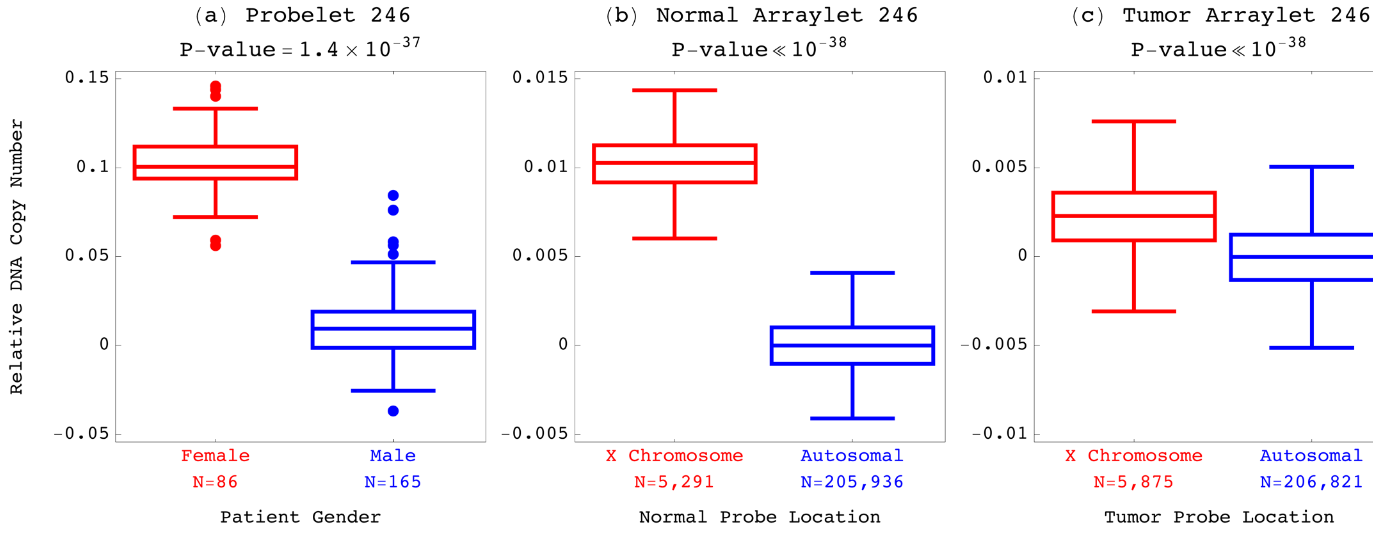


```
Export[path <> "Supplementary_Figure_9.pdf",
  sFig9, "PDF", ImageSize → 750, ImageResolution → resolution];
```

```
(* Compute and Display Kaplan-Meier Survival Curves and Log-Rank Test *)

monthConversion = (365.25 / 12);
Clear[framex, framey, labelx, labely, xlabel, ylabel];

(* Read Survival and Classification Data *)

annotationColumn[annotation_] :=
  Transpose[annotations][[Position[annotationnames, annotation][[1, 1]]]];
options[annotation_] := Intersection[annotationColumn[annotation]];

optionNumbers[annotation_] := Dimensions[options[annotation]][[1]];
optionCounts[annotation_] := Table[Count[annotationColumn[annotation], options[annotation][[a]]],
    {a, 1, optionNumbers[annotation]}];

(* Identify the Two or More Largest Classification Groups *)

order[annotation_] := Sort[optionCounts[annotation], Greater];
first[annotation_] :=
  If[annotation == "Chemotherapy" ||
    annotation == "Probelet_2/Chemotherapy" ||
    annotation == "Arraylet_2/Chemotherapy",
   Position[optionCounts[annotation], order[annotation][[2]]][[1, 1]],
   Position[optionCounts[annotation], order[annotation][[1]]][[1, 1]]];
second[annotation_] :=
  If[annotation == "Chemotherapy",
   Position[optionCounts[annotation], order[annotation][[1]]][[1, 1]],
   If[displayNumber == 2,
    Position[optionCounts[annotation], order[annotation][[2]]][[1, 1]],
    Position[optionCounts[annotation], order[annotation][[3]]][[1, 1]]]];
third[annotation_] :=
  If[annotation == "Probelet_2/Chemotherapy" ||
    annotation == "Arraylet_2/Chemotherapy",
   Position[optionCounts[annotation], order[annotation][[1]]][[1, 1]],
   Position[optionCounts[annotation], order[annotation][[2]]][[1, 1]]];
fourth[annotation_] := Position[optionCounts[annotation], order[annotation][[4]]][[1, 1]];
groups[annotation_] := Sort[Transpose[Join[{annotationColumn[annotation]}, {times}, { status}]]];
optionCountsPosition[annotation_] := Join[{0}, Table[Sum[optionCounts[annotation][[a]], {a, 1, b}],
    {b, 1, Dimensions[optionCounts[annotation]][[1]]}]];
firstGroup[annotation_] := Take[groups[annotation],
   {optionCountsPosition[annotation][[first[annotation]]] + 1,
    optionCountsPosition[annotation][[first[annotation] + 1]]}];
secondGroup[annotation_] := Take[groups[annotation],
   {optionCountsPosition[annotation][[second[annotation]]] + 1,
    optionCountsPosition[annotation][[second[annotation] + 1]]}];
thirdGroup[annotation_] := If[displayNumber ≥ 3,
   Take[groups[annotation],
    {optionCountsPosition[annotation][[third[annotation]]] + 1,
     optionCountsPosition[annotation][[third[annotation] + 1]]}]];
fourthGroup[annotation_] := If[displayNumber ≥ 4,
   Take[groups[annotation],
    {optionCountsPosition[annotation][[fourth[annotation]]] + 1,
     optionCountsPosition[annotation][[fourth[annotation] + 1]]}]];
```

```
(* Evaluate the Kaplan-Meier Survival Curves of the Two or More Largest Groups *)

evaluateFirst[annotation_] := {
  nFirstGroup = optionCounts[annotation][[first[annotation]]];
  oFirstGroup = nFirstGroup;
  group = Transpose[firstGroup[annotation]][[2]];
  Do[If[firstGroup[annotation][[a, 3]] == 0, group = Drop[group, {a}]], {a, nFirstGroup, 1, -1}];
  firstMedian = 0;
  firstLine = {{0, 1}};
  firstLines = {RGBColor[0, 0, 1], Thickness[0.0075]};
  y = 1;
  Do[
   If[firstGroup[annotation][[a, 3]] == 0,
    {oFirstGroup = oFirstGroup - 1;
     firstLines = Join[firstLines, {Line[
         {{firstGroup[annotation][[a, 2]], y + 0.02}, {firstGroup[annotation][[a, 2]], y - 0.02}}]}];
     If[a == optionCounts[annotation][[first[annotation]]],
      firstLine = Join[firstLine, {{firstGroup[annotation][[a, 2]], y}}]]},
    {firstLine = Join[firstLine, {{firstGroup[annotation][[a, 2]], y}}];
     y = y * (nFirstGroup - a) / (nFirstGroup - a + 1);
     If[firstMedian == 0, If[y ≤ 0.5, firstMedian = Round[firstGroup[annotation][[a, 2]]]]];
     firstLine = Join[firstLine, {{firstGroup[annotation][[a, 2]], y}}]}],
   {a, 1, optionCounts[annotation][[first[annotation]]]}];
  firstLine = Graphics[{RGBColor[0, 0, 1], Thickness[0.0075], Line[firstLine]}];
  firstLines = Graphics[firstLines];
  textFirstGroup = Graphics[Text[
     Style[ColumnForm[{
        StringReplace[options[annotation][[first[annotation]]], "_" → " "],
        StringJoin["N=", ToString[nFirstGroup]],
        StringJoin["O=", ToString[oFirstGroup]]},
       Center], {RGBColor[0, 0, 1], FontFamily → "Courier"}],
     If[annotation == "Chemotherapy" ||
       annotation == "Probelet_2/Chemotherapy" ||
       annotation == "Arraylet_2/Chemotherapy",
      {-0.065 * months, 0.1},
      {0.055 * months, 0.1}]]]}

evaluateSecond[annotation_] := {
  nSecondGroup = optionCounts[annotation][[second[annotation]]];
  oSecondGroup = nSecondGroup;
  group = Transpose[secondGroup[annotation]][[2]];
  Do[If[secondGroup[annotation][[a, 3]] == 0, group = Drop[group, {a}]], {a, nSecondGroup, 1, -1}];
  secondMedian = 0;
  secondLine = {{0, 1}};
  secondLines = {RGBColor[1, 0, 0], Thickness[0.0075]};
  y = 1;
  Do[
   If[secondGroup[annotation][[a, 3]] == 0,
    {oSecondGroup = oSecondGroup - 1;
     secondLines = Join[secondLines, {Line[{{secondGroup[annotation][[a, 2]], y + 0.02},
          {secondGroup[annotation][[a, 2]], y - 0.02}}]}];
     If[a == optionCounts[annotation][[second[annotation]]],
      secondLine = Join[secondLine, {{secondGroup[annotation][[a, 2]], y}}]]},
    {secondLine = Join[secondLine, {{secondGroup[annotation][[a, 2]], y}}];
     y = y * (nSecondGroup - a) / (nSecondGroup - a + 1);
     If[secondMedian == 0, If[y ≤ 0.5, secondMedian = Round[secondGroup[annotation][[a, 2]]]]];
     secondLine = Join[secondLine, {{secondGroup[annotation][[a, 2]], y}}]}],
   {a, 1, optionCounts[annotation][[second[annotation]]]}];
  secondLine = Graphics[{RGBColor[1, 0, 0], Thickness[0.0075], Line[secondLine]}];
  secondLines = Graphics[secondLines];
  textSecondGroup = Graphics[
    Text[Style[ColumnForm[{
        StringReplace[options[annotation][[second[annotation]]], "_" → " "],
        StringJoin["N=", ToString[nSecondGroup]],
        StringJoin["O=", ToString[oSecondGroup]]},
       Center], {RGBColor[1, 0, 0], FontFamily → "Courier"}],
     {If[annotation == "Arraylet_2/Chemotherapy" ||
         ylabel == "Patients from the Independent Set",
        0.7, If[secondMedian < 50, 0.8, 0.72]] * months, 0.9}]]}
```

```
evaluateThird[annotation_, displayNumber_] :=
 If[displayNumber ≥ 3, {
   nThirdGroup = optionCounts[annotation][[third[annotation]]];
   oThirdGroup = nThirdGroup;
   group = Transpose[thirdGroup[annotation]][[2]];
   Do[If[thirdGroup[annotation][[a, 3]] == 0, group = Drop[group, {a}]], {a, nThirdGroup, 1, -1}];
   thirdMedian = 0;
   thirdLine = {{0, 1}};
   thirdLines = {RGBColor[0, 0.5, 0], Thickness[0.0075]};
   y = 1;
   Do[
    If[thirdGroup[annotation][[a, 3]] == 0,
     {oThirdGroup = oThirdGroup - 1;
      thirdLines = Join[thirdLines, {Line[{{thirdGroup[annotation][[a, 2]], y + 0.02},
           {thirdGroup[annotation][[a, 2]], y - 0.02}}]}]},
     {thirdLine = Join[thirdLine, {{thirdGroup[annotation][[a, 2]], y}}];
      y = y * (nThirdGroup - a) / (nThirdGroup - a + 1);
      If[thirdMedian == 0, If[y ≤ 0.5, thirdMedian = Round[thirdGroup[annotation][[a, 2]]]]];
      thirdLine = Join[thirdLine, {{thirdGroup[annotation][[a, 2]], y}}]}],
    {a, 1, optionCounts[annotation][[third[annotation]]]}];
   thirdLine = Graphics[{RGBColor[0, 0.5, 0], Thickness[0.0075], Line[thirdLine]}];
   thirdLines = Graphics[thirdLines];
   textThirdGroup = Graphics[
     Text[Style[ColumnForm[{
         StringReplace[options[annotation][[third[annotation]]], "_" → " "],
         StringJoin["N=", ToString[nThirdGroup]],
         StringJoin["O=", ToString[oThirdGroup]]},
        Center], {RGBColor[0, 0.5, 0], FontFamily → "Courier"}],
      {If[annotation == "Arraylet_2/Chemotherapy", 0.7, If[secondMedian < 50, 0.8, 0.72]] * months,
       If[secondMedian < 50, 0.65, 0.35]}]]},
  {thirdLine = Graphics[];
   thirdLines = Graphics[];
   textThirdGroup = Graphics[]}]

evaluateFourth[annotation_, displayNumber_] :=
 If[displayNumber ≥ 4, {
   nFourthGroup = optionCounts[annotation][[fourth[annotation]]];
   oFourthGroup = nFourthGroup;
   group = Transpose[fourthGroup[annotation]][[2]];
   Do[If[fourthGroup[annotation][[a, 3]] == 0, group = Drop[group, {a}]], {a, nFourthGroup, 1, -1}];
   fourthMedian = 0;
   fourthLine = {{0, 1}};
   fourthLines = {RGBColor[0.75, 0, 1], Thickness[0.0075]};
   y = 1;
   Do[
    If[fourthGroup[annotation][[a, 3]] == 0,
     {oFourthGroup = oFourthGroup - 1;
      fourthLines = Join[fourthLines, {Line[{{fourthGroup[annotation][[a, 2]], y + 0.02},
           {fourthGroup[annotation][[a, 2]], y - 0.02}}]}]},
     {fourthLine = Join[fourthLine, {{fourthGroup[annotation][[a, 2]], y}}];
      y = y * (nFourthGroup - a) / (nFourthGroup - a + 1);
      If[fourthMedian == 0, If[y ≤ 0.5, fourthMedian = Round[fourthGroup[annotation][[a, 2]]]]];
      fourthLine = Join[fourthLine, {{fourthGroup[annotation][[a, 2]], y}}]}],
    {a, 1, optionCounts[annotation][[fourth[annotation]]]}];
   fourthLine = Graphics[{RGBColor[0.75, 0, 1], Thickness[0.0075], Line[fourthLine]}];
   fourthLines = Graphics[fourthLines];
   textFourthGroup = Graphics[Text[
      Style[ColumnForm[{
         StringReplace[options[annotation][[fourth[annotation]]], "_" → " "],
         StringJoin["N=", ToString[nFourthGroup]],
         StringJoin["O=", ToString[oFourthGroup]]},
        Center], {RGBColor[0.75, 0, 1], FontFamily → "Courier"}],
      If[annotation == "Probelet_2/Chemotherapy" ||
        annotation == "Arraylet_2/Chemotherapy",
       {-0.065 * months, 0.35},
       {0.055 * months, 0.35}]]]},
  {fourthLine = Graphics[];
   fourthLines = Graphics[];
   textFourthGroup = Graphics[]}]
```

```
(* Evaluate the Log-Rank Test P-value *)

evaluatePValue[annotation_] := {
   statistics = Sort[Transpose[Join[{times}, {annotationColumn[annotation]}, {status}]]];
   firstObservations =
    Table[If[statistics[[a, 2]] == options[annotation][[first[annotation]]], statistics[[a, 3]], 0],
     {a, 1, patients}];
   firstEvents = Table[If[statistics[[a, 2]] == options[annotation][[first[annotation]]],
      ReplaceAll[statistics[[a, 3]], 0 → 1], 0],
     {a, 1, patients}];
   secondObservations = Table[
     If[statistics[[a, 2]] == options[annotation][[second[annotation]]], statistics[[a, 3]], 0],
     {a, 1, patients}];
   secondEvents = Table[If[statistics[[a, 2]] == options[annotation][[second[annotation]]],
      ReplaceAll[statistics[[a, 3]], 0 → 1], 0],
     {a, 1, patients}];
   Do[If[statistics[[a, 1]] == statistics[[a - 1, 1]], {
      firstObservations[[a - 1]] = firstObservations[[a - 1]] + firstObservations[[a]];
      firstObservations = Drop[firstObservations, {a}];
      firstEvents[[a - 1]] = firstEvents[[a - 1]] + firstEvents[[a]];
      firstEvents = Drop[firstEvents, {a}];
      secondObservations[[a - 1]] = secondObservations[[a - 1]] + secondObservations[[a]];
      secondObservations = Drop[secondObservations, {a}];
      secondEvents[[a - 1]] = secondEvents[[a - 1]] + secondEvents[[a]];
      secondEvents = Drop[secondEvents, {a}]
     }], {a, patients, 2, -1}];
   timesNumbers = Dimensions[firstObservations][[1]];
   firstNumbers =
    Table[Total[firstEvents] - Total[Take[firstEvents, 1 ;; a - 1]], {a, 1, timesNumbers}];
   secondNumbers = Table[Total[secondEvents] - Total[Take[secondEvents, 1 ;; a - 1]],
     {a, 1, timesNumbers}];
   observations = firstObservations + secondObservations;
   numbers = firstNumbers + secondNumbers;
   firstExpectations = Table[If[numbers[[a]] == 0, 0,
      N[observations[[a]] * firstNumbers[[a]] / numbers[[a]]]],
     {a, 1, timesNumbers}];
   secondExpectations = Table[If[numbers[[a]] == 0, 0,
      N[observations[[a]] * secondNumbers[[a]] / numbers[[a]]]],
     {a, 1, timesNumbers}];
   variances = Table[If[numbers[[a]] ≤ 1, 0,
      N[firstNumbers[[a]] * secondNumbers[[a]] * observations[[a]] *
        (numbers[[a]] - observations[[a]]) / numbers[[a]] ^2 / (numbers[[a]] - 1)]],
     {a, 1, timesNumbers}];
   z = (N[Total[firstObservations - firstExpectations]]) ^2 / N[Total[variances]];
   pValue = 1 - CDF[ChiSquareDistribution[1], {z}][[1]]};

(* Create the Kaplan-Meier Survival Curves of the Two or More Largest Groups *)

w = 0.0075;
months = If[annotation == "Chemotherapy" ||
     annotation == "Probelet_2/Chemotherapy" ||
     annotation == "Arraylet_2/Chemotherapy", 54, 60];
framey[ylabel_] := ReplaceAll[ReplaceAll[
     Table[{a, Style[If[ylabel == "False", "     ", ToString[a]], FontFamily → "Courier"]},
      {a, 0, 1, 0.25}], "1." → "1"], "0." → "0"];
labelx[xlabel_] := If[xlabel == True, Style["Survival Time (Months)", FontFamily → "Courier"], ""];
plotlabelx[annotation_, xlabel_] :=
  Style[ColumnForm[
    If[hazardRatio == "", {StringJoin[xlabel, " ", StringReplace[annotation, "_" → " "]],
      StringJoin["    P-value = ", ToString[TraditionalForm[
         ScientificForm[pValue, 2, NumberPoint →
           If[Dimensions[Characters[ToString[NumberForm[pValue, 2, NumberFormat → (#1 &),
                  ExponentFunction → (# &)]]]][[1]] < 3, "", "."]]]]]}, {
      StringJoin[xlabel, " ", StringReplace[annotation, "_" → " "]],
      StringJoin["    P-value = ", ToString[TraditionalForm[
         ScientificForm[pValue, 2, NumberPoint →
           If[Dimensions[Characters[ToString[NumberForm[pValue, 2, NumberFormat → (#1 &),
                  ExponentFunction → (# &)]]]][[1]] < 3, "", "."]]
        ]]], StringJoin["    Hazard Ratio", hazardRatio]}], Left], FontFamily → "Courier"];
```

```
labely[ylabel_] :=
  If[ylabel == "False", Style[ColumnForm[{" ", " "}, Center], FontFamily → "Courier"],
   Style[ColumnForm[{title, ylabel}, Center], FontFamily → "Courier"]];
display[annotation_, displayNumber_, xplotlabel_, xlabel_, ylabel_, medianTicks_, highlight_] := {
   evaluateFirst[annotation];
   evaluateSecond[annotation];
   evaluateThird[annotation, displayNumber];
   evaluateFourth[annotation, displayNumber];
   framex = If[displayNumber == 2,
     {{0, Style["0", FontFamily → "Courier"]},
      {firstMedian, If[firstMedian == secondMedian, "", Style[StringJoin[medianTicks[[1]],
          ToString[firstMedian], medianTicks[[2]]], {RGBColor[0, 0, 1], FontFamily → "Courier"}]]},
      {secondMedian, Style[StringJoin[medianTicks[[3]], ToString[secondMedian], medianTicks[[4]]],
        {RGBColor[1, 0, 0], FontFamily → "Courier"}]},
      {40, Style["40", FontFamily → "Courier"]},
      If[annotation == "Chemotherapy",
       {50, Style["50", FontFamily → "Courier"]},
       {60, Style["60", FontFamily → "Courier"]}]},
     {{0, Style["0", FontFamily → "Courier"]},
      {fourthMedian, Style[StringJoin[medianTicks[[7]], If[fourthMedian == firstMedian,
          "", If[fourthMedian == thirdMedian, "", ToString[fourthMedian]]],
         medianTicks[[8]]], {RGBColor[0.75, 0, 1], FontFamily → "Courier"}]},
      {thirdMedian, Style[StringJoin[medianTicks[[5]], ToString[thirdMedian], medianTicks[[6]]],
        {RGBColor[0, 0.5, 0], FontFamily → "Courier"}]},
      {firstMedian, Style[StringJoin[medianTicks[[1]], ToString[firstMedian], medianTicks[[2]]],
        {RGBColor[0, 0, 1], FontFamily → "Courier"}]},
      {secondMedian, Style[StringJoin[medianTicks[[3]], ToString[secondMedian], medianTicks[[4]]],
        {RGBColor[1, 0, 0], FontFamily → "Courier"}]},
      {40, Style["40", FontFamily → "Courier"]},
      If[annotation == "Arraylet_2/Chemotherapy",
       {40, Style["40", FontFamily → "Courier"]},
       If[annotation == "Probelet_2/Chemotherapy",
        {50, Style["50", FontFamily → "Courier"]},
        {60, Style["60", FontFamily → "Courier"]}]]}];
   If[displayNumber == 2, evaluatePValue[annotation]];
   Show[{Graphics[If[highlight == True, {RGBColor[1, 1, 0.8], Rectangle[{
          firstMedian, -0.025}, {secondMedian, 1.025}]}, {}]],
     fourthLine, fourthLines, textFourthGroup,
     thirdLine, thirdLines, textThirdGroup,
     firstLine, firstLines, textFirstGroup,
     secondLine, secondLines, textSecondGroup},
    GridLines → {None, {{0.5, Thickness[0.0025]}}},
    Frame → True,
    FrameTicks → {framex, framey[ylabel], None, None},
    FrameLabel → {labelx[xlabel], labely[ylabel], None, None},
    PlotLabel → plotlabelx[annotation, xplotlabel],
    AspectRatio → If[displayNumber == 2, 1, 1],
    PlotRange → If[annotation == "Chemotherapy" ||
       annotation == "Probelet_2/Chemotherapy" ||
       annotation == "Arraylet_2/Chemotherapy",
      {{-0.225 * months, 1.025 * months}, {-0.025, 1.025}},
      {{-0.1 * months, 1.025 * months}, {-0.025, 1.025}}],
    ImageSize → 250]}
```

```
(* Read TCGA Annotations of the Initial Set of 251 Patients *)

title = "Fraction of Surviving Patients";

stream = path <> "251_Patients.txt";
annotations = Import[stream, "Table"];
{patients, annotationsNumber} = Dimensions[annotations] - {1, 1};
annotationnames = Take[annotations, 1, {2, annotationsNumber + 1}][[1]];
annotations = Drop[Import[stream, "Table"], 1, 1];
Clear[stream];

times = Table[
   If[annotationColumn["Days_Death"][[a]] == "Null",
    If[annotationColumn["Days_Followup"][[a]] == "Null",
     "Null",
     annotationColumn["Days_Followup"][[a]] / monthConversion],
    annotationColumn["Days_Death"][[a]] / monthConversion],
   {a, 1, patients}];
status = Table[If[annotationColumn["Days_Death"][[a]] == "Null",
    If[annotationColumn["Days_Followup"][[a]] == "Null", 2, 0], 1],
   {a, 1, patients}];
positionNull = Position[status, 2];
Do[{
  annotations = Drop[annotations, positionNull[[a]]],
  times = Drop[times, positionNull[[a]]],
  status = Drop[status, positionNull[[a]]]},
 {a, Dimensions[positionNull][[1]], 1, -1}]
patients = patients - Dimensions[positionNull][[1]];

(* Create Survival Analyses of Classification by Chromosome Number Changes *)

displayNumber = 2;

annotation = "Chromosome_10";
highlight = True;
xplotlabel = "(a)";
xlabel = True;
ylabel = "from the Initial Set";
medianTicks = Table["", {a, 1, 8}];
medianTicks[[1]] = "   ";
medianTicks[[4]] = "   ";
hazardRatio = "";
g1 = Show[display[annotation, displayNumber,
    xplotlabel, xlabel, ylabel, medianTicks, highlight], ImageSize → 250];

annotation = "Chromosome_7";
highlight = True;
xplotlabel = "(b)";
xlabel = True;
ylabel = "False";
medianTicks = Table["", {a, 1, 8}];
hazardRatio = "";
g2 = Show[display[annotation, displayNumber,
    xplotlabel, xlabel, ylabel, medianTicks, highlight], ImageSize → 250];

annotation = "Chromosome_9p";
highlight = True;
xplotlabel = "(c)";
xlabel = True;
ylabel = "False";
medianTicks = Table["", {a, 1, 8}];
medianTicks[[1]] = "   ";
medianTicks[[4]] = "   ";
hazardRatio = "";
g3 = Show[display[annotation, displayNumber,
    xplotlabel, xlabel, ylabel, medianTicks, highlight], ImageSize → 250];
```

```
sFig13 = GraphicsGrid[{{g1, g2, g3}}, ImageSize → 750, Spacings → {-42, 2}]
```

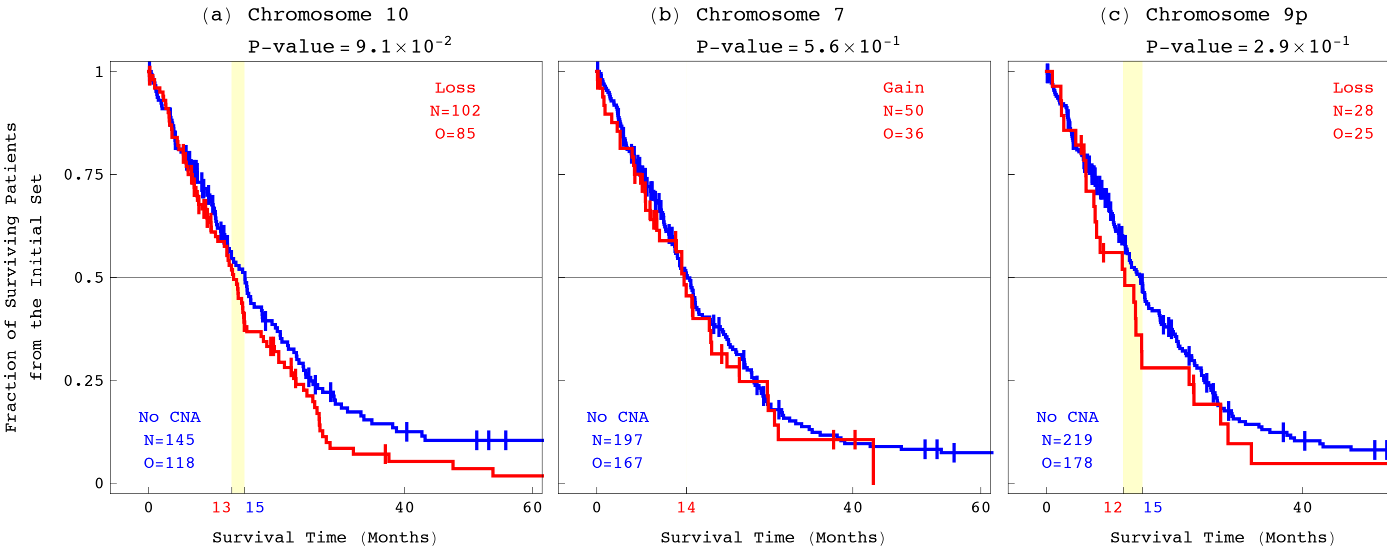


```
Export[path <> "Supplementary_Figure_13.pdf",
  sFig13, "PDF", ImageSize → 750, ImageResolution → resolution];

(* Create Survival Analyses of Classification by Focal CNAs *)

annotation = "MDM4_(Segment_14)";
highlight = True;
xplotlabel = "(a)";
xlabel = False;
ylabel = "from the Initial Set";
medianTicks = Table["", {a, 1, 8}];
hazardRatio = "";
g1 = Show[display[annotation, displayNumber,
     xplotlabel, xlabel, ylabel, medianTicks, highlight], ImageSize → 250];

annotation = "AKT3_(Segment_17)";
highlight = True;
xplotlabel = "(b)";
xlabel = False;
ylabel = "False";
medianTicks = Table["", {a, 1, 8}];
medianTicks[[2]] = " ";
medianTicks[[3]] = " ";
hazardRatio = "";
g2 = Show[display[annotation, displayNumber,
     xplotlabel, xlabel, ylabel, medianTicks, highlight], ImageSize → 250];
```

```
annotation = "EGFR_(Segment_44)";
highlight = True;
xplotlabel = "(c)";
xlabel = False;
ylabel = "False";
medianTicks = Table["", {a, 1, 8}];
medianTicks[[2]] = "  ";
medianTicks[[3]] = "  ";
hazardRatio = "";
g3 = Show[display[annotation, displayNumber,
    xplotlabel, xlabel, ylabel, medianTicks, highlight], ImageSize → 250];

annotation = "MET_(Segment_53)";
highlight = True;
xplotlabel = "(d)";
xlabel = False;
ylabel = "from the Initial Set";
medianTicks = Table["", {a, 1, 8}];
hazardRatio = "";
g4 = Show[display[annotation, displayNumber,
    xplotlabel, xlabel, ylabel, medianTicks, highlight], ImageSize → 250];

annotation = "METTL2B_(Segment_54)";
highlight = True;
xplotlabel = "(e)";
xlabel = False;
ylabel = "False";
medianTicks = Table["", {a, 1, 8}];
hazardRatio = "";
g5 = Show[display[annotation, displayNumber,
    xplotlabel, xlabel, ylabel, medianTicks, highlight], ImageSize → 250];

annotation = "CDKN2A/B_(Segment_62)";
highlight = True;
xplotlabel = "(f)";
xlabel = False;
ylabel = "False";
medianTicks = Table["", {a, 1, 8}];
medianTicks[[2]] = "  ";
medianTicks[[3]] = "  ";
hazardRatio = "";
g6 = Show[display[annotation, displayNumber,
    xplotlabel, xlabel, ylabel, medianTicks, highlight], ImageSize → 250];

annotation = "PTEN_(Segment_80)";
highlight = True;
xplotlabel = "(g)";
xlabel = False;
ylabel = "from the Initial Set";
medianTicks = Table["", {a, 1, 8}];
medianTicks[[1]] = "  ";
medianTicks[[4]] = "  ";
hazardRatio = "";
g7 = Show[display[annotation, displayNumber,
    xplotlabel, xlabel, ylabel, medianTicks, highlight], ImageSize → 250];

annotation = "KDM5A_(Segment_90)";
highlight = True;
xplotlabel = "(h)";
xlabel = False;
ylabel = "False";
medianTicks = Table["", {a, 1, 8}];
medianTicks[[1]] = "  ";
medianTicks[[4]] = "  ";
hazardRatio = "";
g8 = Show[display[annotation, displayNumber,
    xplotlabel, xlabel, ylabel, medianTicks, highlight], ImageSize → 250];
```

```
annotation = "CDK4_(Segment_94)";
highlight = True;
xplotlabel = "(i)";
xlabel = False;
ylabel = "False";
medianTicks = Table["", {a, 1, 8}];
hazardRatio = "";
g9 = Show[display[annotation, displayNumber,
     xplotlabel, xlabel, ylabel, medianTicks, highlight], ImageSize → 250];

annotation = "MDM2_(Segment_102)";
highlight = True;
xplotlabel = "(j)";
xlabel = True;
ylabel = "from the Initial Set";
medianTicks = Table["", {a, 1, 8}];
medianTicks[[2]] = "  ";
medianTicks[[3]] = "  ";
hazardRatio = "";
g10 = Show[display[annotation, displayNumber,
     xplotlabel, xlabel, ylabel, medianTicks, highlight], ImageSize → 250];

annotation = "TLK2/METTL2A_(Segment_112)";
highlight = True;
xplotlabel = "(k)";
xlabel = True;
ylabel = "False";
medianTicks = Table["", {a, 1, 8}];
hazardRatio = "";
g11 = Show[display[annotation, displayNumber,
     xplotlabel, xlabel, ylabel, medianTicks, highlight], ImageSize → 250];

annotation = "CCNE1_(Segment_118)";
highlight = True;
xplotlabel = "(l)";
xlabel = True;
ylabel = "False";
medianTicks = Table["", {a, 1, 8}];
medianTicks[[2]] = "  ";
medianTicks[[3]] = "  ";
hazardRatio = "";
g12 = Show[display[annotation, displayNumber,
     xplotlabel, xlabel, ylabel, medianTicks, highlight], ImageSize → 250];
```

```
sFig14 = GraphicsGrid[
  {{g1, g2, g3}, {g4, g5, g6}, {g7, g8, g9}, {g10, g11, g12}}, ImageSize → 750, Spacings → {-42, 2}]
```

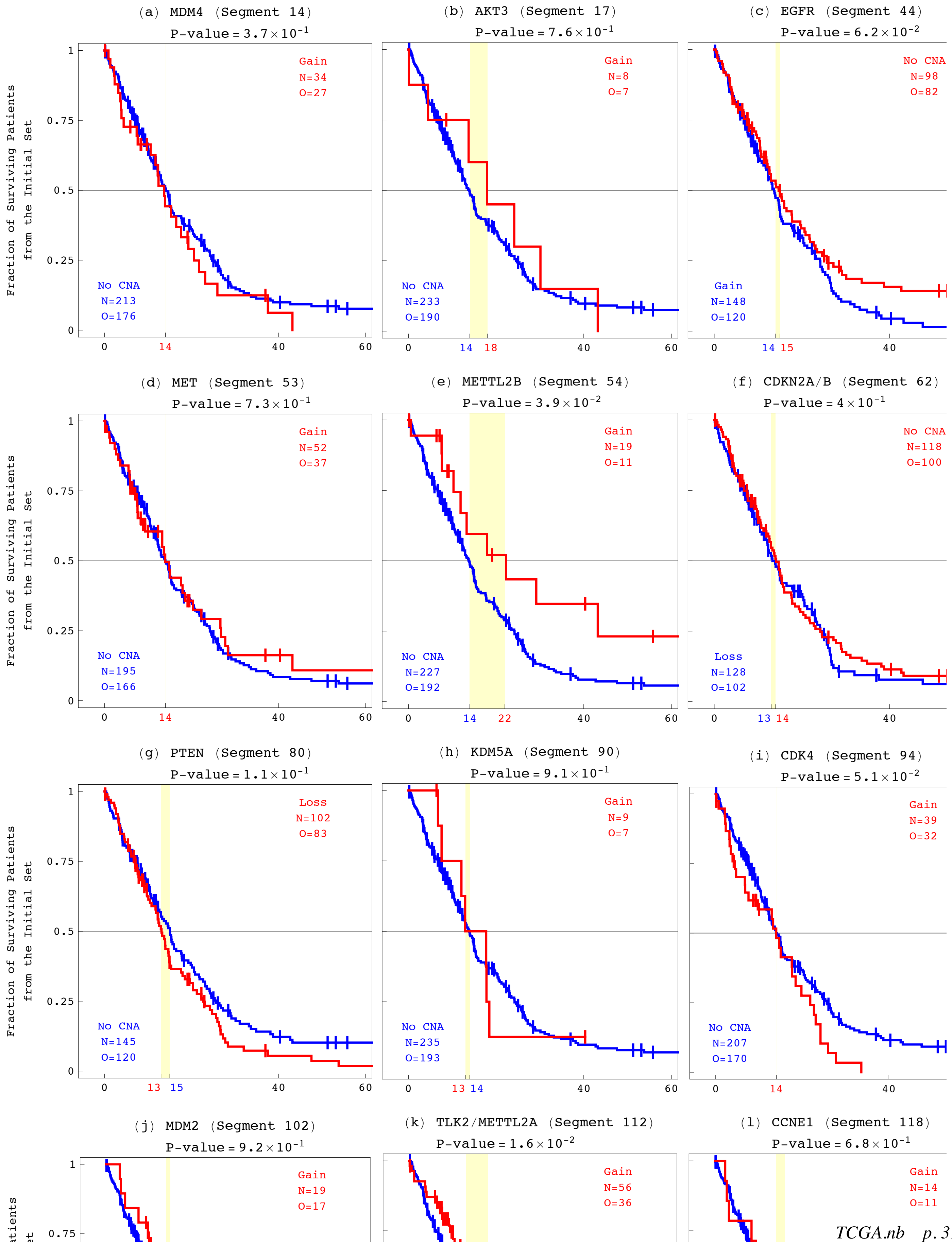

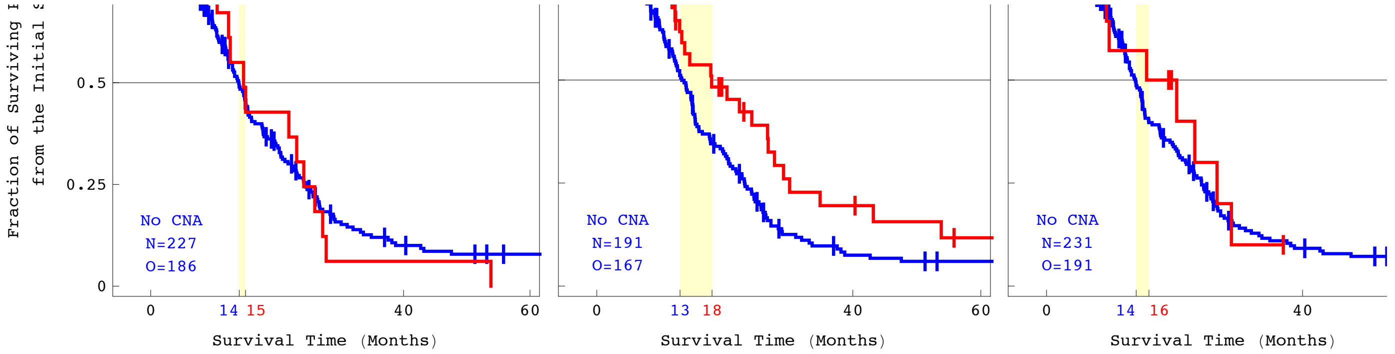


```
Export[path <> "Supplementary_Figure_14.pdf",
  sFig14, "PDF", ImageSize → 750, ImageResolution → resolution];
```

```
(* Create Survival Analysis of Classification by IDH1 Mutation *)
```

```
annotation = "IDH1_Mutation";
highlight = True;
xplotlabel = "    ";
xlabel = True;
ylabel = "from the Initial Set";
medianTicks = Table["", {a, 1, 8}];
hazardRatio = "";
sFig11 = Show[display[annotation, displayNumber,
    xplotlabel, xlabel, ylabel, medianTicks, highlight], ImageSize → 250]
```

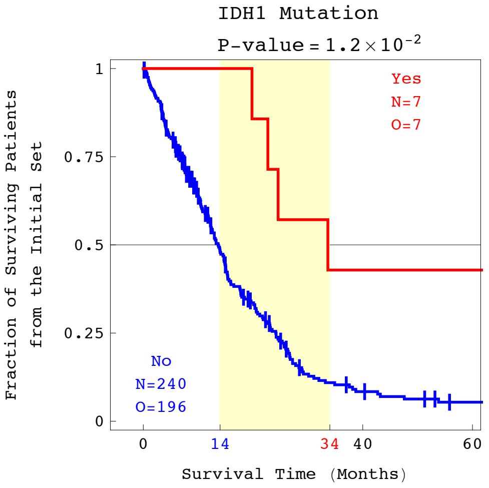


```
Export[path <> "Supplementary_Figure_11.pdf",
  sFig11, "PDF", ImageSize → 250, ImageResolution → resolution];
```

```
(* Create Survival Analyses of Classification by GSVD, Age or Both *)

annotation = "Probelet_2_(Coeff.)";
highlight = True;
xplotlabel = "(a)";
xlabel = False;
ylabel = "from the Initial Set";
medianTicks = Table["", {a, 1, 8}];
hazardRatio = " = 2.3";
g1 = Show[display[annotation, displayNumber,
     xplotlabel, xlabel, ylabel, medianTicks, highlight], ImageSize → 250];

annotation = "Age_(Years)";
highlight = True;
xplotlabel = "(b)";
xlabel = False;
ylabel = "False";
medianTicks = Table["", {a, 1, 8}];
hazardRatio = " = 2";
g2 = Show[display[annotation, displayNumber,
     xplotlabel, xlabel, ylabel, medianTicks, highlight], ImageSize → 250];

displayNumber = 4;

annotation = "Probelet_2/Age";
highlight = True;
xplotlabel = "(c)";
xlabel = False;
ylabel = "False";
medianTicks = Table["", {a, 1, 8}];
medianTicks[[5]] = " ";
pValue = 4.6 * 10^-5;
hazardRatio = "s = 1.8/1.7";
g3 = Show[display[annotation, displayNumber,
     xplotlabel, xlabel, ylabel, medianTicks, highlight], ImageSize → 250];
```

```
(* Read TCGA Annotations of the Inclusive Set of 344 Patients *)

stream = path <> "344_Patients.txt";
annotations = Import[stream, "Table"];
{patients, annotationsNumber} = Dimensions[annotations] - {1, 1};
annotationnames = Take[annotations, 1, {2, annotationsNumber + 1}][[1]];
annotations = Drop[Import[stream, "Table"], 1, 1];
Clear[stream];

times = Table[
   If[annotationColumn["Days_Death"][[a]] == "Null",
    If[annotationColumn["Days_Followup"][[a]] == "Null",
     "Null",
     annotationColumn["Days_Followup"][[a]] / monthConversion],
    annotationColumn["Days_Death"][[a]] / monthConversion],
   {a, 1, patients}];
status = Table[If[annotationColumn["Days_Death"][[a]] == "Null",
    If[annotationColumn["Days_Followup"][[a]] == "Null", 2, 0], 1],
   {a, 1, patients}];
positionNull = Position[status, 2];
Do[{
  annotations = Drop[annotations, positionNull[[a]]],
  times = Drop[times, positionNull[[a]]],
  status = Drop[status, positionNull[[a]]]},
 {a, Dimensions[positionNull][[1]], 1, -1}]
patients = patients - Dimensions[positionNull][[1]];

displayNumber = 2;

annotation = "Probelet_2_(Coeff.)";
highlight = True;
xplotlabel = "(d)";
xlabel = False;
ylabel = "from the Inclusive Confirmation Set";
medianTicks = Table["", {a, 1, 8}];
hazardRatio = "=2.4";
g4 = Show[display[annotation, displayNumber,
    xplotlabel, xlabel, ylabel, medianTicks, highlight], ImageSize → 250];

annotation = "Age_(Years)";
highlight = True;
xplotlabel = "(e)";
xlabel = False;
ylabel = "False";
medianTicks = Table["", {a, 1, 8}];
hazardRatio = "=2";
g5 = Show[display[annotation, displayNumber,
    xplotlabel, xlabel, ylabel, medianTicks, highlight], ImageSize → 250];

displayNumber = 4;

annotation = "Probelet_2/Age";
highlight = True;
xplotlabel = "(f)";
xlabel = False;
ylabel = "False";
medianTicks = Table["", {a, 1, 8}];
medianTicks[[2]] = " ";
medianTicks[[5]] = " ";
medianTicks[[7]] = " ";
pValue = 2. * 10^-6;
hazardRatio = "s=1.9/1.8";
g6 = Show[display[annotation, displayNumber,
    xplotlabel, xlabel, ylabel, medianTicks, highlight], ImageSize → 250];
```

```
(* Read TCGA Annotations of the Independent Set of 184 Patients *)

stream = path <> "184_Patients.txt";
annotations = Import[stream, "Table"];
{patients, annotationsNumber} = Dimensions[annotations] - {1, 1};
annotationnames = Take[annotations, 1, {2, annotationsNumber + 1}][[1]];
annotations = Drop[Import[stream, "Table"], 1, 1];
Clear[stream];

times = Table[
   If[annotationColumn["Days_Death"][[a]] == "Null",
    If[annotationColumn["Days_Followup"][[a]] == "Null",
     "Null",
     annotationColumn["Days_Followup"][[a]] / monthConversion],
    annotationColumn["Days_Death"][[a]] / monthConversion],
   {a, 1, patients}];
status = Table[If[annotationColumn["Days_Death"][[a]] == "Null",
    If[annotationColumn["Days_Followup"][[a]] == "Null", 2, 0], 1],
   {a, 1, patients}];
positionNull = Position[status, 2];
Do[{
  annotations = Drop[annotations, positionNull[[a]]],
  times = Drop[times, positionNull[[a]]],
  status = Drop[status, positionNull[[a]]]},
 {a, Dimensions[positionNull][[1]], 1, -1}]
patients = patients - Dimensions[positionNull][[1]];

displayNumber = 2;

annotation = "Arraylet_2_(Corr.)";
highlight = True;
xplotlabel = "(g)";
xlabel = True;
ylabel = "from the Independent Validation Set";
medianTicks = Table["", {a, 1, 8}];
hazardRatio = "=2.9";
g7 = Show[display[annotation, displayNumber,
    xplotlabel, xlabel, ylabel, medianTicks, highlight], ImageSize → 250];

annotation = "Age_(Years)";
highlight = True;
xplotlabel = "(h)";
xlabel = True;
ylabel = "False";
medianTicks = Table["", {a, 1, 8}];
hazardRatio = "=2.7";
g8 = Show[display[annotation, displayNumber,
    xplotlabel, xlabel, ylabel, medianTicks, highlight], ImageSize → 250];

displayNumber = 4;

annotation = "Arraylet_2/Age";
highlight = True;
xplotlabel = "(i)";
xlabel = True;
ylabel = "False";
medianTicks = Table["", {a, 1, 8}];
medianTicks[[5]] = "   ";
pValue = 2.6 * 10^-6;
hazardRatio = "s=2/2.2";
g9 = Show[display[annotation, displayNumber,
    xplotlabel, xlabel, ylabel, medianTicks, highlight], ImageSize → 250];
```

```
fig3 = GraphicsGrid[{{g1, g2, g3}, {g4, g5, g6}, {g7, g8, g9}}, ImageSize → 750, Spacings → {-56, 0}]
```

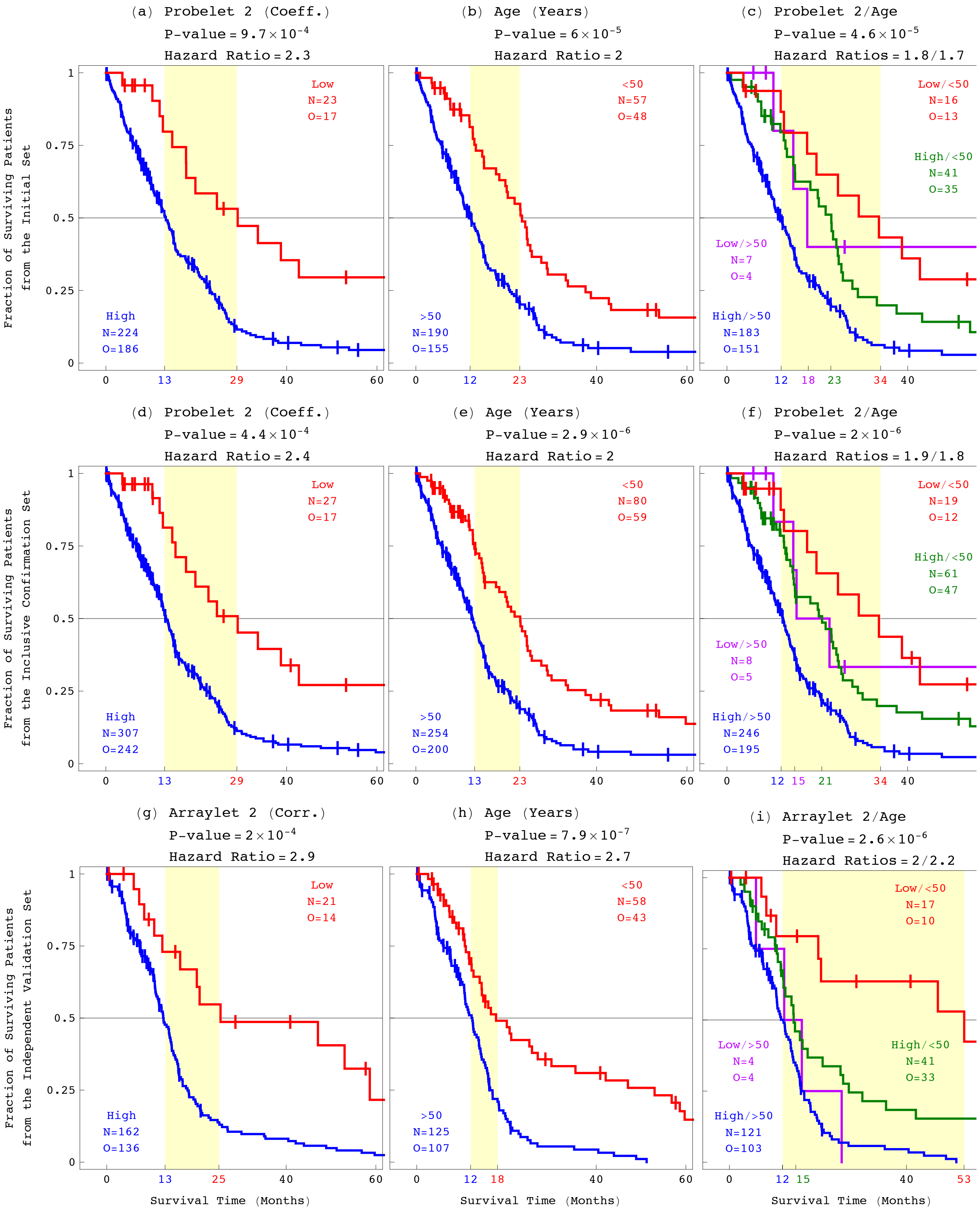


```
Export[path <> "Figure_3.pdf", fig3, "PDF", ImageSize → 750, ImageResolution → resolution];
```

```
(* Create Survival Analyses of Classification by Chemotherapy or GSVD/Chemotherapy *)

(* Read TCGA Annotations of the Initial Set of 251 Patients *)

stream = path <> "251_Patients.txt";
annotations = Import[stream, "Table"];
{patients, annotationsNumber} = Dimensions[annotations] - {1, 1};
annotationnames = Take[annotations, 1, {2, annotationsNumber + 1}][[1]];
annotations = Drop[Import[stream, "Table"], 1, 1];
Clear[stream];

times = Table[
   If[annotationColumn["Days_Death"][[a]] == "Null",
    If[annotationColumn["Days_Followup"][[a]] == "Null",
     "Null",
     annotationColumn["Days_Followup"][[a]] / monthConversion],
    annotationColumn["Days_Death"][[a]] / monthConversion],
   {a, 1, patients}];
status = Table[
   If[annotationColumn["Probelet_2/Chemotherapy"][[a]] == "Null", 2,
    If[annotationColumn["Days_Death"][[a]] == "Null",
     If[annotationColumn["Days_Followup"][[a]] == "Null", 2, 0], 1]],
   {a, 1, patients}];
positionNull = Position[status, 2];
Do[{
  annotations = Drop[annotations, positionNull[[a]]],
  times = Drop[times, positionNull[[a]]],
  status = Drop[status, positionNull[[a]]]},
 {a, Dimensions[positionNull][[1]], 1, -1}]
patients = patients - Dimensions[positionNull][[1]];

displayNumber = 2;

annotation = "Chemotherapy";
highlight = True;
xplotlabel = "(a)";
xlabel = False;
ylabel = "from the Initial Set";
medianTicks = Table["", {a, 1, 8}];
hazardRatio = "=2.6";
g1 = Show[display[annotation, displayNumber,
    xplotlabel, xlabel, ylabel, medianTicks, highlight], ImageSize → 250];

displayNumber = 4;

annotation = "Probelet_2/Chemotherapy";
highlight = True;
xplotlabel = "(b)";
xlabel = False;
ylabel = "False";
medianTicks = Table["", {a, 1, 8}];
pValue = 6.1 * 10^-15;
hazardRatio = "s=3/3.1";
g2 = Show[display[annotation, displayNumber,
    xplotlabel, xlabel, ylabel, medianTicks, highlight], ImageSize → 250];
```

```
(* Read TCGA Annotations of the Inclusive Set of 344 Patients *)

stream = path <> "344_Patients.txt";
annotations = Import[stream, "Table"];
{patients, annotationsNumber} = Dimensions[annotations] - {1, 1};
annotationnames = Take[annotations, 1, {2, annotationsNumber + 1}][[1]];
annotations = Drop[Import[stream, "Table"], 1, 1];
Clear[stream];

times = Table[
   If[annotationColumn["Days_Death"][[a]] == "Null",
    If[annotationColumn["Days_Followup"][[a]] == "Null",
     "Null",
     annotationColumn["Days_Followup"][[a]] / monthConversion],
    annotationColumn["Days_Death"][[a]] / monthConversion],
   {a, 1, patients}];
status = Table[
   If[annotationColumn["Probelet_2/Chemotherapy"][[a]] == "Null", 2,
    If[annotationColumn["Days_Death"][[a]] == "Null",
     If[annotationColumn["Days_Followup"][[a]] == "Null", 2, 0], 1]],
   {a, 1, patients}];
positionNull = Position[status, 2];
Do[{
  annotations = Drop[annotations, positionNull[[a]]],
  times = Drop[times, positionNull[[a]]],
  status = Drop[status, positionNull[[a]]]},
 {a, Dimensions[positionNull][[1]], 1, -1}]
patients = patients - Dimensions[positionNull][[1]];

displayNumber = 2;

annotation = "Chemotherapy";
highlight = True;
xplotlabel = "(c)";
xlabel = False;
ylabel = "from the Inclusive Confirmation Set";
medianTicks = Table["", {a, 1, 8}];
hazardRatio = " = 2.7";
g3 = Show[display[annotation, displayNumber,
    xplotlabel, xlabel, ylabel, medianTicks, highlight], ImageSize → 250];

displayNumber = 4;

annotation = "Probelet_2/Chemotherapy";
highlight = True;
xplotlabel = "(d)";
xlabel = False;
ylabel = "False";
medianTicks = Table["", {a, 1, 8}];
pValue = 5.8 * 10^-18;
hazardRatio = "s = 3.1/3.2";
g4 = Show[display[annotation, displayNumber,
    xplotlabel, xlabel, ylabel, medianTicks, highlight], ImageSize → 250];
```

```
(* Read TCGA Annotations of the Independent Set of 184 Patients *)

stream = path <> "184_Patients.txt";
annotations = Import[stream, "Table"];
{patients, annotationsNumber} = Dimensions[annotations] - {1, 1};
annotationnames = Take[annotations, 1, {2, annotationsNumber + 1}][[1]];
annotations = Drop[Import[stream, "Table"], 1, 1];
Clear[stream];

times = Table[
   If[annotationColumn["Days_Death"][[a]] == "Null",
    If[annotationColumn["Days_Followup"][[a]] == "Null",
     "Null",
     annotationColumn["Days_Followup"][[a]] / monthConversion],
    annotationColumn["Days_Death"][[a]] / monthConversion],
   {a, 1, patients}];
status = Table[
   If[annotationColumn["Arraylet_2/Chemotherapy"][[a]] == "Null", 2,
    If[annotationColumn["Days_Death"][[a]] == "Null",
     If[annotationColumn["Days_Followup"][[a]] == "Null", 2, 0], 1]],
   {a, 1, patients}];
positionNull = Position[status, 2];
Do[{
  annotations = Drop[annotations, positionNull[[a]]],
  times = Drop[times, positionNull[[a]]],
  status = Drop[status, positionNull[[a]]]},
 {a, Dimensions[positionNull][[1]], 1, -1}]
patients = patients - Dimensions[positionNull][[1]];

displayNumber = 2;

annotation = "Chemotherapy";
highlight = True;
xplotlabel = "(e)";
xlabel = True;
ylabel = "from the Independent Validation Set";
medianTicks = Table["", {a, 1, 8}];
hazardRatio = "=2.2";
g5 = Show[display[annotation, displayNumber,
    xplotlabel, xlabel, ylabel, medianTicks, highlight], ImageSize → 250];

displayNumber = 4;

annotation = "Arraylet_2/Chemotherapy";
highlight = True;
xplotlabel = "(f)";
xlabel = True;
ylabel = "False";
medianTicks = Table["", {a, 1, 8}];
pValue = 5.9 * 10^-8;
hazardRatio = "s=3.3/2.7";
g6 = Show[display[annotation, displayNumber,
    xplotlabel, xlabel, ylabel, medianTicks, highlight], ImageSize → 250];
```

```
sFig16 = GraphicsGrid[{{g1, g2}, {g3, g4}, {g5, g6}}, ImageSize → 500, Spacings → {-56, 0}]
```

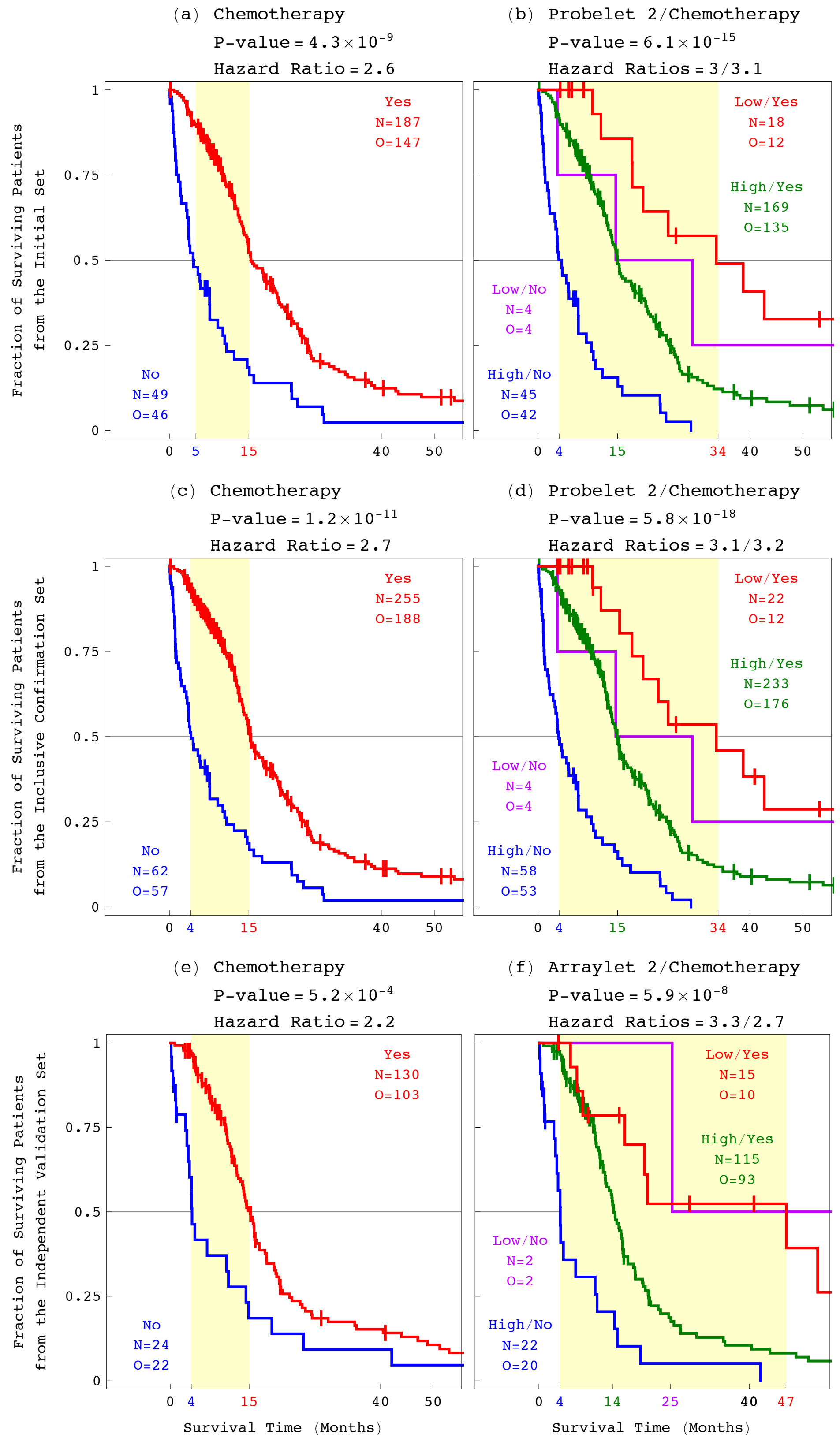


```
Export[path <> "Supplementary_Figure_16.pdf",
  sFig16, "PDF", ImageSize → 500, ImageResolution → resolution];
```

```
(* Create Survival Analyses of Classification of Chemotherapy Patients by Focal CNAs *)

title = "Fraction of Surviving Chemotherapy";

stream = path <> "251_Patients.txt";
annotations = Import[stream, "Table"];
{patients, annotationsNumber} = Dimensions[annotations] - {1, 1};
annotationnames = Take[annotations, 1, {2, annotationsNumber + 1}][[1]];
annotations = Drop[Import[stream, "Table"], 1, 1];
Clear[stream];

times = Table[
   If[annotationColumn["Days_Death"][[a]] == "Null",
    If[annotationColumn["Days_Followup"][[a]] == "Null",
     "Null",
     annotationColumn["Days_Followup"][[a]] / monthConversion],
    annotationColumn["Days_Death"][[a]] / monthConversion],
   {a, 1, patients}];
status = Table[
   If[annotationColumn["Chemotherapy"][[a]] == "Null", 2,
    If[annotationColumn["Chemotherapy"][[a]] == "No", 2,
     If[annotationColumn["Days_Death"][[a]] == "Null",
      If[annotationColumn["Days_Followup"][[a]] == "Null", 2, 0], 1]]],
   {a, 1, patients}];
positionNull = Position[status, 2];
Do[{
  annotations = Drop[annotations, positionNull[[a]]],
  times = Drop[times, positionNull[[a]]],
  status = Drop[status, positionNull[[a]]]},
 {a, Dimensions[positionNull][[1]], 1, -1}]
patients = patients - Dimensions[positionNull][[1]];

displayNumber = 2;

annotation = "MDM4_(Segment_14)";
highlight = True;
xplotlabel = "(a)";
xlabel = False;
ylabel = "Patients from the Initial Set";
medianTicks = Table["", {a, 1, 8}];
medianTicks[[2]] = "   ";
medianTicks[[3]] = "   ";
hazardRatio = "";
g1 = Show[display[annotation, displayNumber,
    xplotlabel, xlabel, ylabel, medianTicks, highlight], ImageSize → 250];

annotation = "AKT3_(Segment_17)";
highlight = True;
xplotlabel = "(b)";
xlabel = False;
ylabel = "False";
medianTicks = Table["", {a, 1, 8}];
medianTicks[[2]] = " ";
medianTicks[[3]] = " ";
hazardRatio = "";
g2 = Show[display[annotation, displayNumber,
    xplotlabel, xlabel, ylabel, medianTicks, highlight], ImageSize → 250];
```

```
annotation = "EGFR_(Segment_44)";
highlight = True;
xplotlabel = "(c)";
xlabel = False;
ylabel = "False";
medianTicks = Table["", {a, 1, 8}];
medianTicks[[2]] = "  ";
medianTicks[[3]] = "  ";
hazardRatio = "";
g3 = Show[display[annotation, displayNumber,
     xplotlabel, xlabel, ylabel, medianTicks, highlight], ImageSize → 250];

annotation = "MET_(Segment_53)";
highlight = True;
xplotlabel = "(d)";
xlabel = False;
ylabel = "Patients from the Initial Set";
medianTicks = Table["", {a, 1, 8}];
medianTicks[[2]] = " ";
medianTicks[[3]] = " ";
hazardRatio = "";
g4 = Show[display[annotation, displayNumber,
     xplotlabel, xlabel, ylabel, medianTicks, highlight], ImageSize → 250];

annotation = "METTL2B_(Segment_54)";
highlight = True;
xplotlabel = "(e)";
xlabel = False;
ylabel = "False";
medianTicks = Table["", {a, 1, 8}];
hazardRatio = "";
g5 = Show[display[annotation, displayNumber,
     xplotlabel, xlabel, ylabel, medianTicks, highlight], ImageSize → 250];

annotation = "CDKN2A/B_(Segment_62)";
highlight = True;
xplotlabel = "(f)";
xlabel = False;
ylabel = "False";
medianTicks = Table["", {a, 1, 8}];
medianTicks[[1]] = " ";
medianTicks[[4]] = " ";
hazardRatio = "";
g6 = Show[display[annotation, displayNumber,
     xplotlabel, xlabel, ylabel, medianTicks, highlight], ImageSize → 250];

annotation = "PTEN_(Segment_80)";
highlight = True;
xplotlabel = "(g)";
xlabel = False;
ylabel = "Patients from the Initial Set";
medianTicks = Table["", {a, 1, 8}];
medianTicks[[1]] = " ";
medianTicks[[4]] = " ";
hazardRatio = "";
g7 = Show[display[annotation, displayNumber,
     xplotlabel, xlabel, ylabel, medianTicks, highlight], ImageSize → 250];

annotation = "KDM5A_(Segment_90)";
highlight = True;
xplotlabel = "(h)";
xlabel = False;
ylabel = "False";
medianTicks = Table["", {a, 1, 8}];
medianTicks[[1]] = "  ";
medianTicks[[4]] = "  ";
hazardRatio = "";
g8 = Show[display[annotation, displayNumber,
     xplotlabel, xlabel, ylabel, medianTicks, highlight], ImageSize → 250];
```

```
annotation = "CDK4_(Segment_94)";
highlight = True;
xplotlabel = "(i)";
xlabel = False;
ylabel = "False";
medianTicks = Table["", {a, 1, 8}];
medianTicks[[2]] = " ";
medianTicks[[3]] = " ";
hazardRatio = "";
g9 = Show[display[annotation, displayNumber,
     xplotlabel, xlabel, ylabel, medianTicks, highlight], ImageSize → 250];

annotation = "MDM2_(Segment_102)";
highlight = True;
xplotlabel = "(j)";
xlabel = True;
ylabel = "Patients from the Initial Set";
medianTicks = Table["", {a, 1, 8}];
medianTicks[[1]] = "  ";
medianTicks[[4]] = "  ";
hazardRatio = "";
g10 = Show[display[annotation, displayNumber,
     xplotlabel, xlabel, ylabel, medianTicks, highlight], ImageSize → 250];

annotation = "TLK2/METTL2A_(Segment_112)";
highlight = True;
xplotlabel = "(k)";
xlabel = True;
ylabel = "False";
medianTicks = Table["", {a, 1, 8}];
hazardRatio = "";
g11 = Show[display[annotation, displayNumber,
     xplotlabel, xlabel, ylabel, medianTicks, highlight], ImageSize → 250];

annotation = "CCNE1_(Segment_118)";
highlight = True;
xplotlabel = "(l)";
xlabel = True;
ylabel = "False";
medianTicks = Table["", {a, 1, 8}];
medianTicks[[2]] = "  ";
medianTicks[[3]] = "  ";
hazardRatio = "";
g12 = Show[display[annotation, displayNumber,
     xplotlabel, xlabel, ylabel, medianTicks, highlight], ImageSize → 250];
```

```
sFig15 = GraphicsGrid[{{g1, g2, g3}, {g4, g5, g6}, {g7, g8, g9}, {g10, g11, g12}},
  ImageSize → 750, Spacings → {-42, 2}]
```

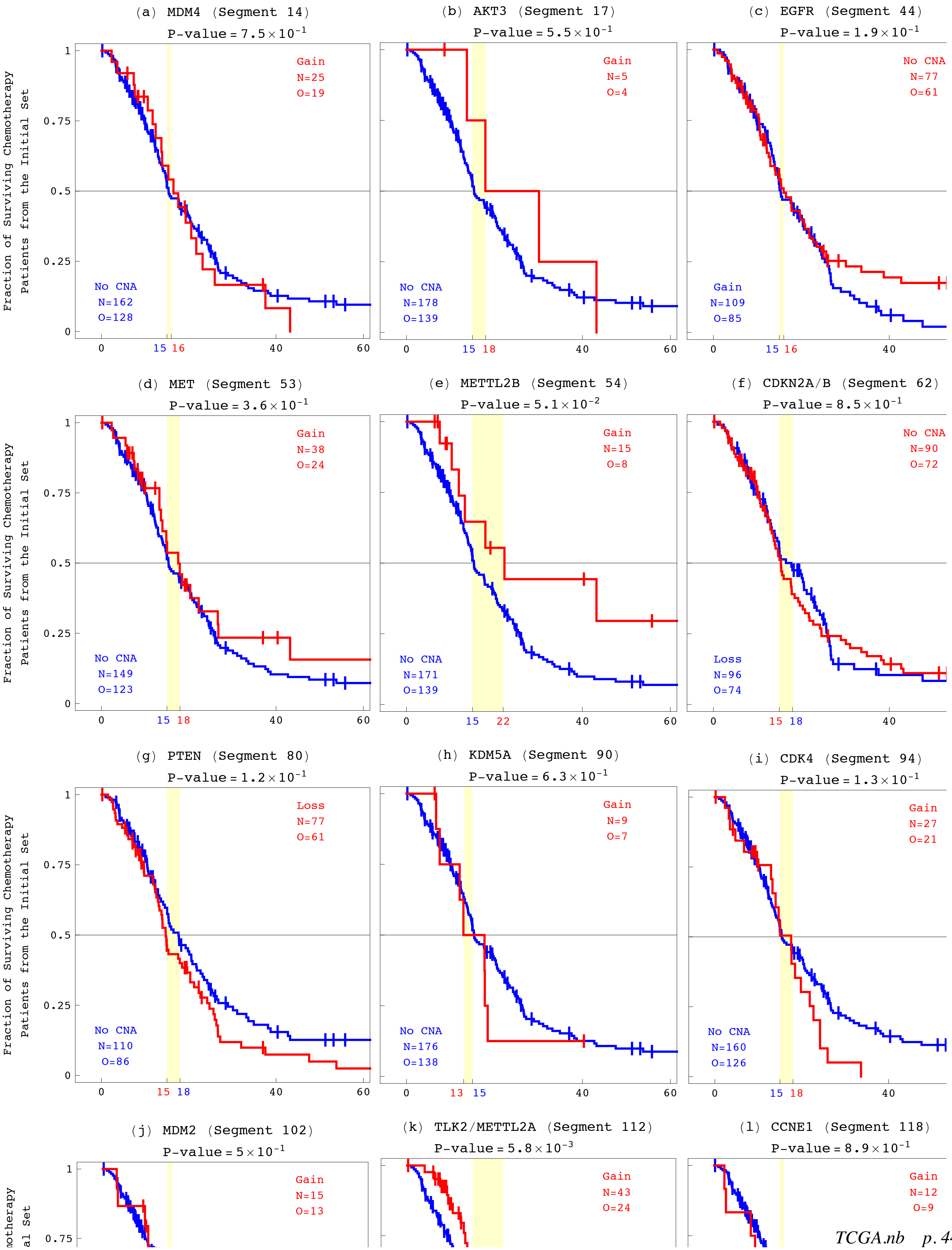

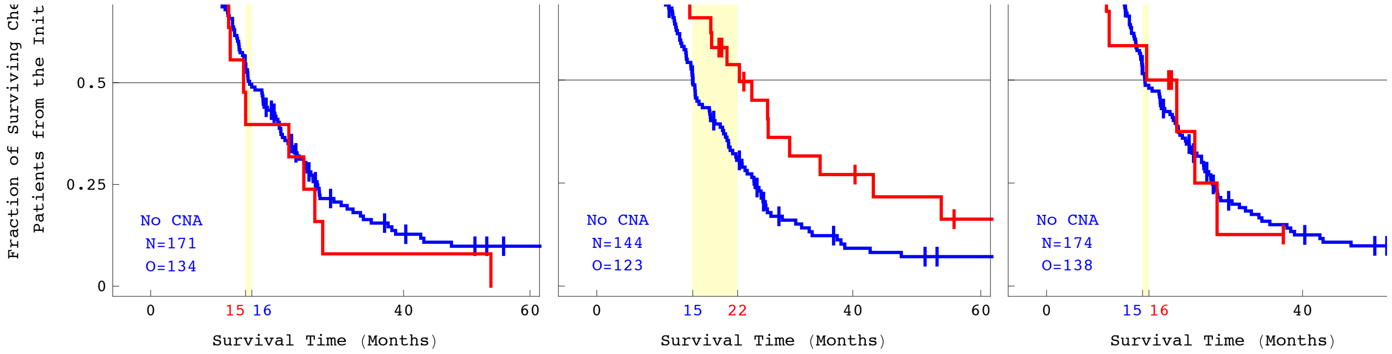


```
Export[path <> "Supplementary_Figure_15.pdf",
  sFig15, "PDF", ImageSize → 750, ImageResolution → resolution];

(* Create Survival Analysis of Classification of Chemotherapy Patients by IDH1 Mutation *)

annotation = "IDH1_Mutation";
highlight = True;
xplotlabel = "    ";
xlabel = True;
ylabel = "Patients from the Initial Set";
medianTicks = Table["", {a, 1, 8}];
hazardRatio = "";
sFig12 = Show[display[annotation, displayNumber,
    xplotlabel, xlabel, ylabel, medianTicks, highlight], ImageSize → 250]
```

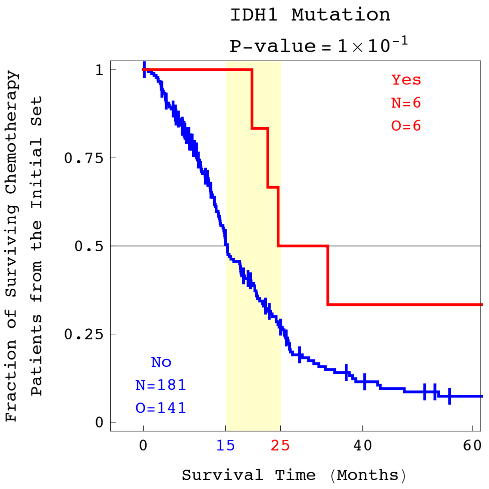


```
Export[path <> "Supplementary_Figure_12.pdf",
  sFig12, "PDF", ImageSize → 250, ImageResolution → resolution];

(* Create Survival Analysis of GSVD Classification of Chemotherapy Patients *)

annotation = "Probelet_2_(Coeff.)";
highlight = True;
xplotlabel = "(a)";
xlabel = False;
ylabel = "Patients from the Initial Set";
medianTicks = Table["", {a, 1, 8}];
hazardRatio = "";
g1 = Show[display[annotation, displayNumber,
     xplotlabel, xlabel, ylabel, medianTicks, highlight], ImageSize → 250];
```

```
(* Read TCGA Annotations of the Inclusive Set of 344 Patients *)

stream = path <> "344_Patients.txt";
annotations = Import[stream, "Table"];
{patients, annotationsNumber} = Dimensions[annotations] - {1, 1};
annotationnames = Take[annotations, 1, {2, annotationsNumber + 1}][[1]];
annotations = Drop[Import[stream, "Table"], 1, 1];
Clear[stream];

times = Table[
   If[annotationColumn["Days_Death"][[a]] == "Null",
    If[annotationColumn["Days_Followup"][[a]] == "Null",
     "Null",
     annotationColumn["Days_Followup"][[a]] / monthConversion],
    annotationColumn["Days_Death"][[a]] / monthConversion],
   {a, 1, patients}];
status = Table[
   If[annotationColumn["Chemotherapy"][[a]] == "Null", 2,
    If[annotationColumn["Chemotherapy"][[a]] == "No", 2,
     If[annotationColumn["Days_Death"][[a]] == "Null",
      If[annotationColumn["Days_Followup"][[a]] == "Null", 2, 0], 1]]],
   {a, 1, patients}];
positionNull = Position[status, 2];
Do[{
  annotations = Drop[annotations, positionNull[[a]]],
  times = Drop[times, positionNull[[a]]],
  status = Drop[status, positionNull[[a]]]},
 {a, Dimensions[positionNull][[1]], 1, -1}]
patients = patients - Dimensions[positionNull][[1]];

annotation = "Probelet_2_(Coeff.)";
highlight = True;
xplotlabel = "(b)";
xlabel = False;
ylabel = "Patients from the Inclusive Set";
medianTicks = Table["", {a, 1, 8}];
hazardRatio = "";
g2 = Show[display[annotation, displayNumber,
    xplotlabel, xlabel, ylabel, medianTicks, highlight], ImageSize → 250];
```

```
(* Read TCGA Annotations of the Independent Set of 184 Patients *)

stream = path <> "184_Patients.txt";
annotations = Import[stream, "Table"];
{patients, annotationsNumber} = Dimensions[annotations] - {1, 1};
annotationnames = Take[annotations, 1, {2, annotationsNumber + 1}][[1]];
annotations = Drop[Import[stream, "Table"], 1, 1];
Clear[stream];

times = Table[
   If[annotationColumn["Days_Death"][[a]] == "Null",
    If[annotationColumn["Days_Followup"][[a]] == "Null",
     "Null",
     annotationColumn["Days_Followup"][[a]] / monthConversion],
    annotationColumn["Days_Death"][[a]] / monthConversion],
   {a, 1, patients}];
status = Table[
   If[annotationColumn["Chemotherapy"][[a]] == "Null", 2,
    If[annotationColumn["Chemotherapy"][[a]] == "No", 2,
     If[annotationColumn["Days_Death"][[a]] == "Null",
      If[annotationColumn["Days_Followup"][[a]] == "Null", 2, 0], 1]]],
   {a, 1, patients}];
positionNull = Position[status, 2];
Do[{
  annotations = Drop[annotations, positionNull[[a]]],
  times = Drop[times, positionNull[[a]]],
  status = Drop[status, positionNull[[a]]]},
 {a, Dimensions[positionNull][[1]], 1, -1}]
patients = patients - Dimensions[positionNull][[1]];

annotation = "Arraylet_2_(Corr.)";
highlight = True;
xplotlabel = "(c)";
xlabel = True;
ylabel = "Patients from the Independent Set";
medianTicks = Table["", {a, 1, 8}];
hazardRatio = "";
g3 = Show[display[annotation, displayNumber,
    xplotlabel, xlabel, ylabel, medianTicks, highlight], ImageSize → 250];
```

```
sFig10 = GraphicsGrid[{{g1}, {g2}, {g3}}, ImageSize → 250, Spacings → {-56, 0}]
```

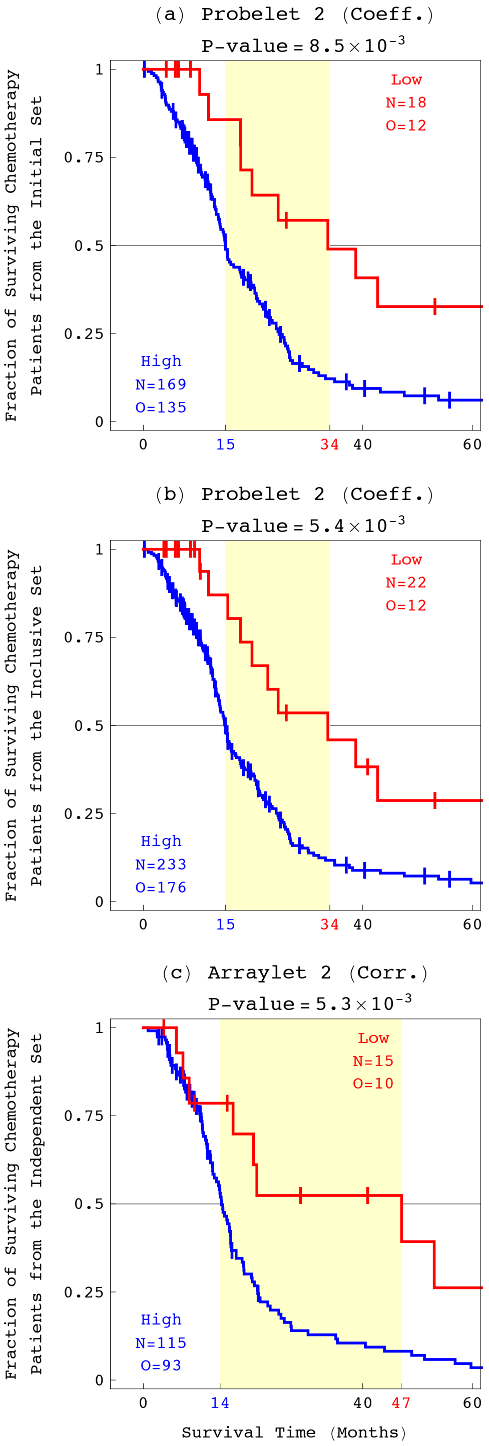


```
Export[path <> "Supplementary_Figure_10.pdf",
  sFig10, "PDF", ImageSize → 250, ImageResolution → resolution];
```
